# Quantitative assessment of the determinant structural differences between redox-active and inactive glutaredoxins

**DOI:** 10.1038/s41467-020-15441-3

**Published:** 2020-04-07

**Authors:** Linda Liedgens, Jannik Zimmermann, Lucas Wäschenbach, Fabian Geissel, Hugo Laporte, Holger Gohlke, Bruce Morgan, Marcel Deponte

**Affiliations:** 1 Fachbereich Chemie, Abteilung Biochemie, Technische Universität Kaiserslautern, D-67663 Kaiserslautern, Germany; 2 Institut für Biochemie, Zentrum für Human- und Molekularbiologie (ZHMB), Universität des Saarlandes, D-66123 Saarbrücken, Germany; 3 Mathematisch-Naturwissenschaftliche Fakultät, Institut für Pharmazeutische und Medizinische Chemie, Heinrich-Heine-Universität Düsseldorf, D-40225 Düsseldorf, Germany; 4 John von Neumann Institute for Computing (NIC), Jülich Supercomputing Centre (JSC) & Institute of Complex Systems, ICS-6: Structural Biochemistry, Forschungszentrum Jülich GmbH, D-52425 Jülich, Germany

**Keywords:** Biocatalysis, Enzymes, Thioredoxins

## Abstract

Class I glutaredoxins are enzymatically active, glutathione-dependent oxidoreductases, whilst class II glutaredoxins are typically enzymatically inactive, Fe-S cluster-binding proteins. Enzymatically active glutaredoxins harbor both a glutathione-scaffold site for reacting with glutathionylated disulfide substrates and a glutathione-activator site for reacting with reduced glutathione. Here, using yeast ScGrx7 as a model protein, we comprehensively identified and characterized key residues from four distinct protein regions, as well as the covalently bound glutathione moiety, and quantified their contribution to both interaction sites. Additionally, we developed a redox-sensitive GFP2-based assay, which allowed the real-time assessment of glutaredoxin structure-function relationships inside living cells. Finally, we employed this assay to rapidly screen multiple glutaredoxin mutants, ultimately enabling us to convert enzymatically active and inactive glutaredoxins into each other. In summary, we have gained a comprehensive understanding of the mechanistic underpinnings of glutaredoxin catalysis and have elucidated the determinant structural differences between the two main classes of glutaredoxins.

## Abstract (web summary)

Glutaredoxins play a central role in numerous biological processes including cellular redox homeostasis and Fe-S cluster biogenesis. Here the authors establish the molecular basis for glutaredoxin redox catalysis through comprehensive biochemical and structural analyses.

## Introduction

Canonical glutaredoxins (Grx), also referred to as class I Grx, are enzymatically active in standard in vitro oxidoreductase assays. On the contrary, Grx-like proteins, also referred to as class II Grx, bind iron–sulfur clusters and have very little or no oxidoreductase activity (reviewed in refs. ^1–7^). For the sake of simplicity, hereinafter we classify the two protein subfamilies as “enzymatically active Grx” or “inactive Grx”, respectively. However, we do not exclude the possibility that inactive Grx might, in some cases, catalyze redox reactions with specialized substrates in vivo.

**Fig. 1:**
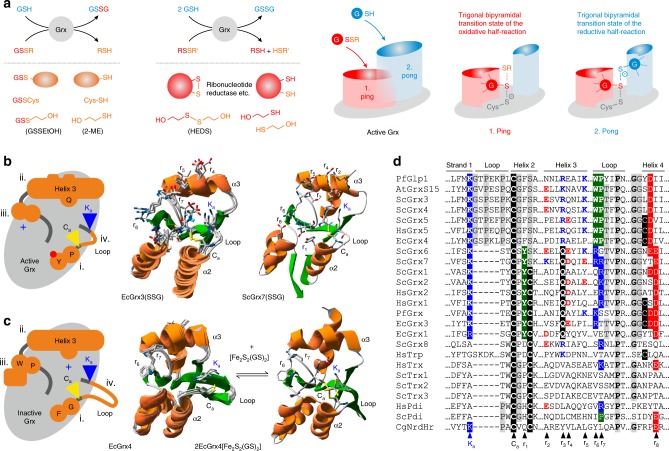
Structural differences between enzymatically active and inactive Grx. **a** Enzymatically active glutaredoxins (Grx) use reduced glutathione (GSH) as an electron donor for the reduction of high- and low-molecular weight glutathione disulfide substrates (GSSR) or non-glutathione disulfide substrates (RSSR’) as highlighted in the left half of the panel. The Grx-catalyzed reduction of GSSR by GSH is separated into an oxidative and reductive half-reaction as highlighted by the two predicted transition states and glutathione interaction sites in the schematic representations in the right half of the panel. **b** Structure of enzymatically active Grx. A schematic representation of four specialized protein areas, NMR solution structures of the glutathionylated C14S mutant of enzymatically active EcGrx3 (PDB entry 3GRX), and a model of glutathionylated ScGrx7 are shown from left to right. Please note that the glutathione moiety as well as the conserved active-site cysteine (C_a_) and lysine (K_a_) residue adopt several alternative positions in the NMR structures. **c** Structure of enzymatically inactive Grx. A schematic representation of four specialized protein areas, NMR solution structures of enzymatically inactive EcGrx4 (PDB entry 1YKA), and the crystal structure of EcGrx4 in complex with an iron–sulfur cluster (PDB entry 2WCI, one EcGrx4 subunit and one GS^−^ ligand were omitted for clarity) are shown from left to right. Please note the conformational change of the elongated loop and the repositioning of the active-site cysteine and lysine residue upon iron–sulfur cluster binding. **d** Sequence alignment of glutaredoxin isoforms and comparison with other proteins of the thioredoxin superfamily from *A. thaliana* (At), *S. cerevisiae* (Sc), *Homo sapiens* (Hs), *E. coli* (Ec), *P. falciparum* (Pf), and *C. glutamicum* (Cg). Established as well as potential glutathione-interacting residues r_1–8_ are highlighted.

Enzymatically active Grx use glutathione as a substrate^3,4,7–10^ (Fig. 1a, b), whereas inactive Grx use glutathione as a ligand for their iron–sulfur cluster^1,2,5,6^ (Fig. 1c). How both Grx subfamilies exert their nonredundant physiological functions in redox catalysis and iron metabolism remains puzzling, and the underlying structure–function relationships are only partially understood. We recently confirmed that enzymatically active Grx have two distinct glutathione-interaction sites, one glutathione-scaffold site that interacts with glutathionylated disulfide substrates (GSSR) during the oxidative half-reaction, and one glutathione-activator site that interacts with reduced glutathione (GSH) during the reductive half-reaction^7,11,12^ (Fig. 1a). Based on these results, we hypothesized that modified glutathione interactions underlie the enzymatic inactivity of class II Grx and kinetically uncouple these proteins from the glutathione pool^7,12,13^. Kinetic studies revealed that the glutathione-scaffold site reflects the glutathione interaction that was previously identified in numerous X-ray and nuclear magnetic resonance (NMR) structures of both Grx subfamilies^4,12^. In contrast, the glutathione-activator site remains predominantly uncharacterized except for a highly conserved lysine residue^12^. The relevance of this residue as an activator was recently corroborated by a study on the lysine-deficient *Trypanosoma brucei* homolog TbGrx1 which reacts with GSSG but does not accept GSH as a reducing agent^14^.

The aim of this work therefore was, first, to identify protein areas that are relevant for redox catalysis and that are modified in enzymatically inactive Grx and, second, to quantitatively assess the contribution of these protein areas toward the oxidoreductase activity. At least four protein areas differ between enzymatically active and inactive Grx (Fig. 1b–d): (i) The active site of all Grx contains an essential cysteine residue for catalysis or iron–sulfur cluster binding at the N-terminus of helix 2. This residue is followed in most enzymatically active Grx by a proline, a tyrosine and a second cysteine residue in a CPYC-motif, whereas a glycine, a phenylalanine and a serine residue are usually found in a CGFS-motif in inactive Grx. With the exception of the poorly active hybrid protein ScGrx8 from yeast^11^, the second cysteine residue of enzymatically active Grx is dispensable for the reduction of glutathionylated substrates and the low-molecular weight model substrate bis(2-hydroxyethyl)disulfide (HEDS)^15–25^. Accordingly, attempts to activate the Grx-like protein 1 from *Plasmodium falciparum* by simply introducing a second cysteine residue failed^26^. In contrast to glutathionylated substrates, the reduction of specific, non-glutathionylated protein disulfides such as *Escherichia coli* ribonucleotide reductase requires the second cysteine residue of active Grx^10,17,27,28^. The second cysteine might also help to resolve kinetically trapped enzyme conformations^4,11^. The proline residue in the CPYC-motif prevents iron–sulfur cluster binding. It is replaced in a few enzymatically active Grx that can also bind [Fe_2_S_2_] clusters^19,21,29–32^. The tyrosine hydroxyl group of the CPYC-motif, which is absent in the CGFS-motif of inactive Grx, protrudes from the protein surface and was hypothesized to contribute to the glutathione-activator site^12^. The residue is replaced by aspartate in poorly active ScGrx8, by histidine in protein disulfide isomerases and by proline in thioredoxins^11,12,33^. (ii) Helix 3 is part of the glutathione-scaffold site and differs significantly between both Grx subfamilies^4,12,34,35^. It harbors a conserved glutamine residue and also comprises residues that protrude from the protein surface and that might contribute to the glutathione-activator site^12^. (iii) A conserved WP-motif in the loop between helix 3 and strand 3 is characteristic of inactive Grx and is usually replaced by one or two basic residues in enzymatically active Grx^4,19,26,34–37^. (iv) The most striking feature of enzymatically inactive Grx is an elongated loop between the highly conserved lysine residue and the active-site cysteine residue^4,34,36^. Comparisons between the structures of monomeric apoprotein and homodimeric holoprotein of EcGrx4 from *E. coli* as well as HsGrx5 from human revealed significant rearrangements of this insertion and a repositioning of the cysteine and lysine residue upon [Fe_2_S_2_] cluster binding^34,36,38^. The insertion was hypothesized to be the major cause for the enzymatic inactivity of class II Grx^19^.

Here, we systematically analyzed protein areas (i)–(iv) for recombinant mutant proteins in steady-state kinetic assays in vitro, for redox reporter-tagged constructs in yeast, and for molecular dynamics simulations in silico. Using the enzymatically active CPYS-type model protein ScGrx7 from yeast as well as the inactive CGFS-type homolog HsGrx5, we further characterized the glutathione-scaffold and glutathione-activator site. We show that protein areas (i)–(iv) all contribute to Grx catalysis and demonstrate that the flanking lysine and tyrosine residue do not affect the thiol p*K*_a_ value of the active-site cysteine residue but rather stabilize the transition states. Furthermore, our data suggests that the elongated active-site loop acts as an off-switch in enzymatically inactive Grx. Finally, as a proof-of-principle, we show that by replacing key structural motifs we could interconvert enzymatically active and inactive glutaredoxins, respectively.

## Results

### The hydroxyl group of Tyr110 is dispensable for catalysis

**Fig. 2:**
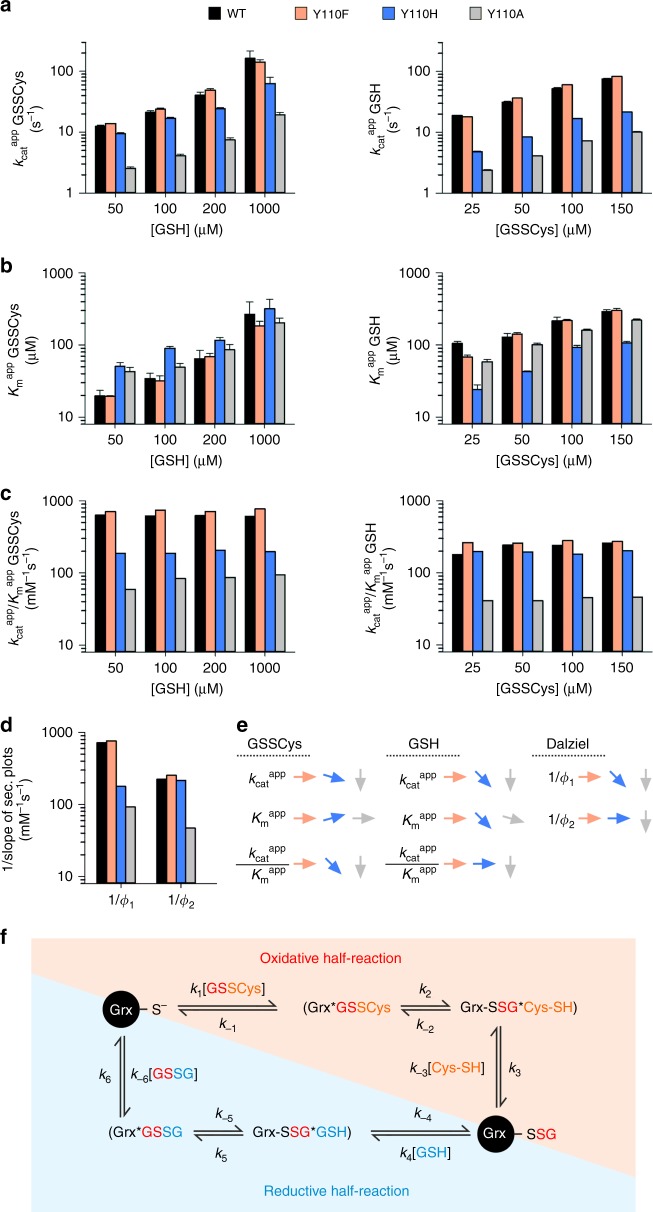
Tyr110 of ScGrx7 is part of the glutathione-scaffold site in the GSSCys assay. **a**, **b** Selected $k_{{\mathrm{cat}}}^{{\mathrm{app}}}$ and $K_{\mathrm{m}}^{{\mathrm{app}}}$ values of ScGrx7 wild-type enzyme and Y110F/H/A mutants for GSSCys and GSH. **c** Calculated catalytic efficiencies from panels (**a**) and (**b**). **d** Reciprocal Dalziel coefficients, which probably reflect the second order rate constants of the oxidative and reductive half-reaction with GSSCys and GSH, respectively. **e** Summary of the altered kinetic parameters for Y110F/H/A. **f** Reaction sequence for the GSSCys assay in accordance with the observed ping-pong kinetics. Please note that the $K_{\mathrm{m}}^{{\mathrm{app}}}$ values are not solely defined by the ratios *k*_−1_/*k*_1_ and *k*_−4_/*k*_4_ but are also affected by other rate constants. Hence, the $K_{\mathrm{m}}^{{\mathrm{app}}}$ values do not reflect true substrate affinities as shown previously^12^. Source data are provided in the Supplementary Information: original plots and kinetic parameters for panels **a**–**c** are shown in Supplementary Fig. 2 and Supplementary Table 2. Statistical analyses and *P* values for the $k_{{\mathrm{cat}}}^{{\mathrm{app}}}$ and $K_{\mathrm{m}}^{{\mathrm{app}}}$ values from panels **a** and **b** are listed in Supplementary Table 11. Error bars are the calculated standard error from the curve fits in SigmaPlot 13. Reciprocal Dalziel coefficients for panel **d** were obtained from Supplementary Fig. 3 and are listed together with the true *k*_cat_ values in Supplementary Table 1.

Most active Grxs harbor a conserved tyrosine residue in their catalytic CPY(C/S)-motif. This residue is typically replaced by phenylalanine in most inactive Grxs (r_1_ in Fig. 1). We first therefore purified recombinant wild-type ScGrx7 as well as the ScGrx7 mutant Y110F. In addition, we prepared the ScGrx7 mutant Y110H to investigate the requirement for an aromatic side chain and the mutant Y110A, which lacks any aromatic side chain or possibility for hydrogen bonding, as a control (Fig. 2, Supplementary Figs. 1–6, Supplementary Tables 1–3). Replacement of Tyr110 by phenylalanine had rather minor effects on the $k_{{\mathrm{cat}}}^{{\mathrm{app}}}$ and $K_{\mathrm{m}}^{{\mathrm{app}}}$ values in the GSSCys assay (Fig. 2a, b). The catalytic efficiencies $\left( {k_{{\mathrm{cat}}}^{{\mathrm{app}}}/K_{\mathrm{m}}^{{\mathrm{app}}}} \right)$ and reciprocal Dalziel coefficients (1/Φ) of the Y110F mutant were almost identical or even slightly increased as compared with the wild-type enzyme (Fig. 2c–e). These parameters can be interpreted as the second order rate constant for the oxidative half-reaction with GSSCys, yielding glutathionylated enzyme and cysteine, and the reductive half-reaction with GSH, yielding reduced enzyme and glutathione disulfide (GSSG)^12,39^ (Fig. 2f). Thus, both half-reactions appeared to be unaffected by the removal of the hydroxyl group. In contrast to the GSSCys assay, catalytic efficiencies and reciprocal Dalziel coefficients of Y110F in the HEDS assay were decreased by 20–56% (Supplementary Fig. 4). In this assay, ScGrx7 can directly react with HEDS, yielding 2-mercaptoethanol (2-ME) and a mixed disulfide between GSH and 2-mercaptoethanol (GSSEtOH). GSSEtOH must change its orientation at the active site before it can be reduced by a second GSH molecule, yielding 2-ME and GSSG^12,25^. In summary, the hydroxyl group of the conserved active-site tyrosine residue is dispensable for the GSH-dependent reduction of GSSCys and does not play a general role as a GSH activator. However, the hydroxyl group can affect the turnover of non-glutathione disulfide substrates.

### Tyr110 forms part of the glutathione-scaffold site

In accordance with preliminary results^12^, replacement of Tyr110 of ScGrx7 by alanine decreased the catalytic efficiency and reciprocal Dalziel coefficient for GSSCys to 18–23% and for GSH to 9–15% of the wild-type enzyme (Fig. 2). Catalytic efficiencies and reciprocal Dalziel coefficients of Y110A in the HEDS assay were decreased to 3–6% of the wild-type enzyme (Supplementary Fig. 4). Removal of the bulky aromatic side chain therefore affected both the oxidative and the reductive half-reaction of the enzyme. An intermediate effect was observed for the mutant Y110H, which had an approximately three to four times lower catalytic efficiency and reciprocal Dalziel coefficient for GSSCys, but an almost unchanged catalytic efficiency and reciprocal Dalziel coefficient for GSH (Fig. 2). Thus, replacement of the phenyl moiety by the basic imidazole side chain in Y110H impaired the interaction with GSSCys during the oxidative half-reaction but not with GSH during the reductive half-reaction. As a result, the oxidative half-reaction became rate-limiting for Y110H. In summary, the side chain of Tyr110 plays an important structural role and contributes to the glutathione-scaffold site as revealed for mutants Y110H and Y110A. The unaffected reaction rate between Y110F/H and GSH appears to be based on the bulky aromatic side chain that keeps the glutathione moiety of the glutathionylated enzyme and its transition state in a correct orientation. Removal of the aromatic side chain in Y110A presumably alters this orientation and, therefore, indirectly decreases the rate constant with GSH during the reductive half-reaction.

### Lys105 and Tyr110 do not affect the thiol p*K*_a_ value of Cys108

**Fig. 3:**
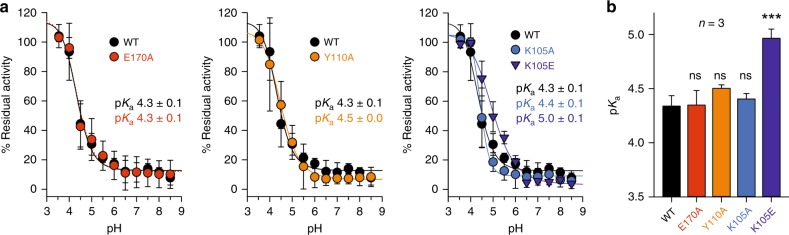
The side chains of Tyr110 and Lys105 do not affect the p*K*_a_-value of the ScGrx7 active site thiol group. Freshly reduced wild-type ScGrx7 (black symbols) and selected mutants (colored symbols) were incubated with 150 µM iodoacetamide at 23 °C for 180 s in a three-buffer system at pH 3.5–8.5. Residual activities were measured in a standard HEDS assay and normalized against mock controls that were incubated in parallel without iodoacetamide. **a** Data for wild-type ScGrx7 (WT) and the mutants E170A, Y110A, K105A, and K105E. Samples containing iodoacetamide were apparently more stable at lower pH values than the mock controls resulting in residual activities slightly above 100%. The p*K*_a_ values from the sigmoidal fits are indicated. Data points and error bars represent the mean ± s.d. of three independent experiments. **b** Summary and statistical analyses of the p*K*_a_ values from panel (**a**). Sigmoidal fits using the four parameter Hill function and *P* values from one way ANOVA analyses followed by a Holm–Sidak test were calculated in SigmaPlot 13 (*P* > 0.05: ns; *P* ≤ 0.001: ***). Source data are provided as a Source Data file.

Based on studies on human Grx1 and NrdH from *Corynebacterium glutamicum*^40,41^, we previously suggested that Lys105 (residue *K*_a_ in Fig. 1) might also stabilize the thiolate of the active-site cysteine residue of ScGrx7 (ref. ^12^). We now tested this hypothesis for wild-type ScGrx7 as well as the mutants K105A/E and Y110A. Following incubation of the recombinant ScGrx7 mutants with iodoacetamide at a range of different pH values, the residual enzymatic activity was determined in a standard HEDS assay (Fig. 3). Residue Glu170 (r_8_ in Fig. 1), which contributes to the glutathione-scaffold site and is far away from Cys108 (ref. ^12^), was replaced by alanine and served as a negative control. Replacement of Lys105 or Tyr110 by alanine had no significant effect on the p*K*_a_ value of the thiol group of Cys108, whereas the replacement of Lys105 by glutamate increased the p*K*_a_ value from 4.3 ± 0.1 to 5.0 ± 0.1. In summary, the thiol p*K*_a_ value of the free enzyme is unaffected by the positive charge of Lys105 or the side chain of Tyr110, and even the introduction of an additional negative charge in the proximity of the active-site cysteine residue has only a moderate effect on its protonation state. We therefore suggest that both residues rather stabilize the conformation of the free and glutathionylated enzyme as well as its negatively charged transition states.

### Helix 3 contributes to the scaffold site and GSH recruitment

**Fig. 4:**
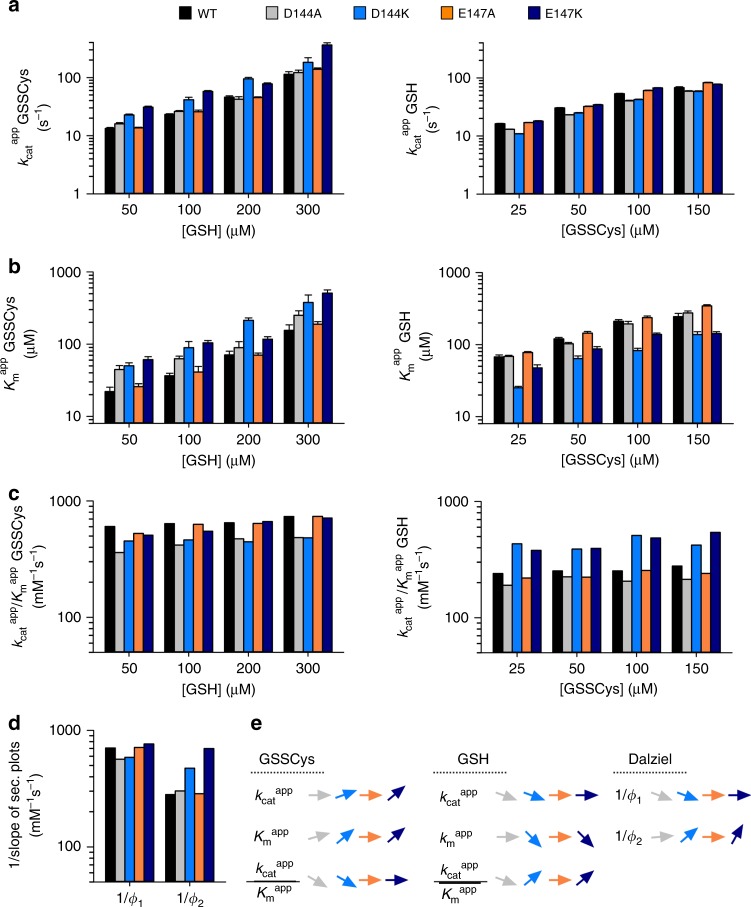
Lysine replacements of Asp144 or Glu147 in helix 3 of ScGrx7 accelerate the reductive half-reaction in the GSSCys assay. **a**, **b** Selected $k_{{\mathrm{cat}}}^{{\mathrm{app}}}$ and $K_{\mathrm{m}}^{{\mathrm{app}}}$ values of ScGrx7 wild-type enzyme as well as D144A/K and E147A/K mutants for GSSCys and GSH. **c** Calculated catalytic efficiencies from panels (**a**) and (**b**). **d** Reciprocal Dalziel coefficients, which probably reflect the rate constants of the oxidative and reductive half-reaction with GSSCys and GSH, respectively. **e** Summary of the altered kinetic parameters for D144A/K and E147A/K. Source data are provided in the Supplementary Information: original plots and kinetic parameters for panels **a**–**c** are shown in Supplementary Figs. 7 and 9 and Supplementary Table 5. Statistical analyses and *P* values for the $k_{{\mathrm{cat}}}^{{\mathrm{app}}}$ and $K_{\mathrm{m}}^{{\mathrm{app}}}$ values from panels **a** and **b** are listed in Supplementary Table 11. Error bars are the calculated standard error from the curve fits in SigmaPlot 13. Reciprocal Dalziel coefficients for panel **d** were obtained from Supplementary Figs. 8 and 10 and are listed together with the true *k*_cat_ values in Supplementary Table 4.

Residues Asp144 and Glu147 (r_4_ and r_5_ in Fig. 1) in helix 3 of ScGrx7 were suggested to contribute to the glutathione-activator site because they protrude from the protein surface on top of the glutathione-scaffold site^12^. We addressed this hypothesis for the mutants D144A/K and E147A/K in the GSSCys assay in vitro (Fig. 4, Supplementary Figs. 1 and 7–10, Supplementary Tables 4 and 5). Wild-type ScGrx7 was studied in parallel and served as a control for systematic variations. Replacement of Asp144 by alanine and lysine slightly decreased the reciprocal Dalziel coefficient for GSSCys by 17–20%, whereas replacement of Glu147 by alanine and lysine had no effect on the catalytic efficiency and reciprocal Dalziel coefficient for GSSCys (Fig. 4). The alanine replacement of Asp144 and Glu147 in D144A and E147A also had no effect on the catalytic efficiency and reciprocal Dalziel coefficient for GSH. In contrast, lysine replacements in D144K and E147K yielded gain-of-function mutants with 1.7- and 2.5-fold increased reciprocal Dalziel coefficients for GSH. In summary, the charge-inversion mutants D144K and E147K have an accelerated reductive-half-reaction with GSH in accordance with the hypothesis that helix 3 not only contributes to the glutathione-scaffold site but also plays a role for the recruitment of GSH by surface-exposed residues.

### The basic loop following helix 3 is part of the scaffold site

**Fig. 5:**
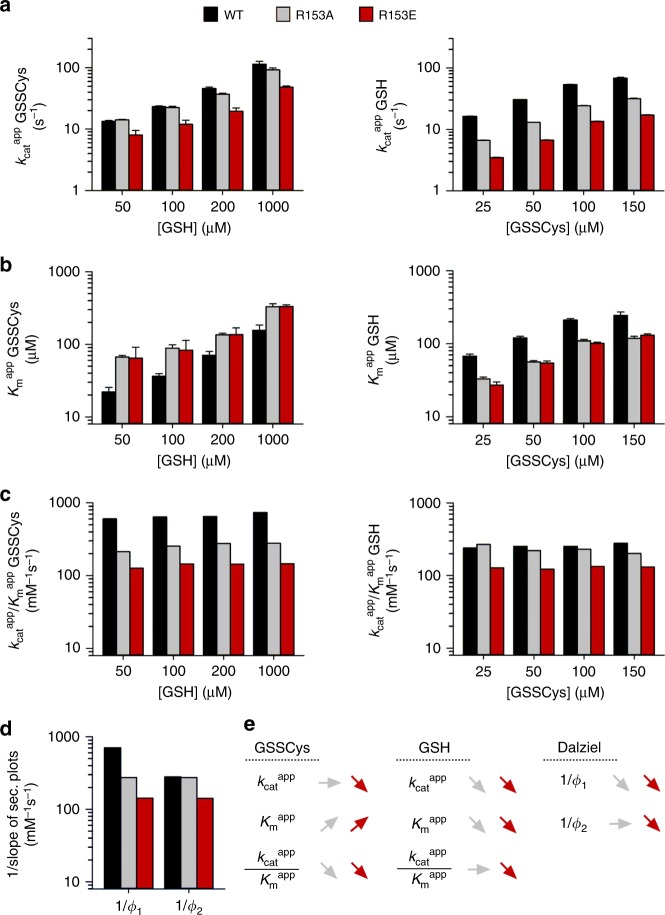
Arg153 of ScGrx7 is part of the glutathione-scaffold site in the GSSCys assay. **a**, **b** Selected $k_{{\mathrm{cat}}}^{{\mathrm{app}}}$ and $K_{\mathrm{m}}^{{\mathrm{app}}}$ values of ScGrx7 wild-type enzyme and R153A/E mutants for GSSCys and GSH. **c** Calculated catalytic efficiencies from panels (**a**) and (**b**). **d** Reciprocal Dalziel coefficients, which probably reflect the rate constants of the oxidative and reductive half-reaction with GSSCys and GSH, respectively. **e** Summary of the altered kinetic parameters for R153A/E. Source data are provided in the Supplementary Information: original plots and kinetic parameters for panels **a**–**c** are shown in Supplementary Fig. 11 and Supplementary Table 6. Statistical analyses and *P* values for the $k_{{\mathrm{cat}}}^{{\mathrm{app}}}$ and $K_{\mathrm{m}}^{{\mathrm{app}}}$ values from panels **a** and **b** are listed in Supplementary Table 11. Error bars are the calculated standard error from the curve fits in SigmaPlot 13. Reciprocal Dalziel coefficients for panel **d** were obtained from Supplementary Fig. 12 and are listed together with the true *k*_cat_ values in Supplementary Table 4.

Residue Arg153 (r_7_ in Fig. 1) in the loop between helix 3 and strand 3 of ScGrx7 also protrudes from the protein surface and is therefore a candidate for the recruitment of GSH. We therefore analyzed the ScGrx7 mutants R153A/E in the GSSCys assay in vitro (Fig. 5, Supplementary Figs. 1, 11, and 12, Supplementary Tables 4 and 6). Wild-type ScGrx7 was studied in parallel and served as a control for systematic variations. The alanine replacement of Arg153 reduced the catalytic efficiency and reciprocal Dalziel coefficient for GSSCys by 60% but had only a minor effect on the catalytic efficiency and reciprocal Dalziel coefficient for GSH. In contrast, the charge inversion in R153E also affected the reductive half-reaction with GSH. The reductive half-reactions of both mutants were as fast as their oxidative half-rections, suggesting that the glutathionylation of the mutants became the rate-limiting step for catalysis. In summary, residue Arg153 plays a crucial role for the glutathione-scaffold site and its replacement can result in a rate-limiting oxidative half-reaction.

### Interconversion studies of active and inactive Grx

**Fig. 6:**
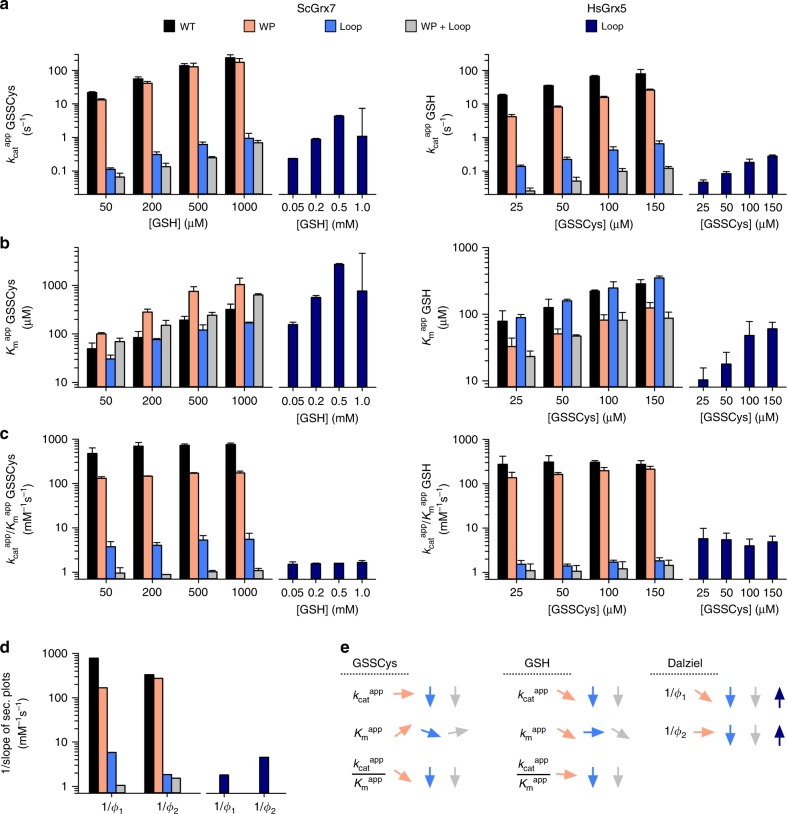
The active-site loop is a determinant structural difference between enzymatically active and inactive Grx. **a**, **b** Selected $k_{{\mathrm{cat}}}^{{\mathrm{app}}}$ and $K_{\mathrm{m}}^{{\mathrm{app}}}$ values of ScGrx7 wild-type enzyme, ScGrx7^WP^, ScGrx7^loop^, ScGrx7^WP+loop^, and HsGrx5^loop^. **c** Calculated catalytic efficiencies from panels (**a**) and (**b**). **d** Reciprocal Dalziel coefficients, which probably reflect the rate constants of the oxidative and reductive half-reaction with GSSCys and GSH, respectively. **e** Summary of the altered kinetic parameters. Source data are provided in the Supplementary Information: original plots and kinetic parameters for panels **a**–**c** are shown in Supplementary Figs. 13 and 15 and Supplementary Tables 8 and 9. Statistical analyses and *P* values for the $k_{{\mathrm{cat}}}^{{\mathrm{app}}}$ and $K_{\mathrm{m}}^{{\mathrm{app}}}$ values from panels **a** and **b** are listed in Supplementary Table 11. Error bars are the calculated standard error from the curve fits in SigmaPlot 13. Reciprocal Dalziel coefficients for panel **d** were obtained from Supplementary Figs. 14 and 16 and are listed together with the true *k*_cat_ values in Supplementary Table 7.

Next, we analyzed the predicted relevance of the WP-motif and the elongated active-site loop for enzyme inactivation^19^ with the intention to convert enzymatically inactive and active Grx into each other. We therefore replaced either residues Arg152/Arg153 (r_6_ and r_7_ in Fig. 1) of ScGrx7 by a WP-motif or the short TG loop of ScGrx7 with the elongated GTPEQPQ loop of HsGrx5. In addition, we generated mutants that contained both replacements. The recombinant mutants ScGrx7^WP^, ScGrx7^loop^, and ScGrx7^WP+loop^ were subsequently compared with wild-type ScGrx7, which was studied in parallel and served as a control for systematic variations. Likewise, we replaced the WP-motif of HsGrx5 by the RR-motif of ScGrx7, the elongated GTPEQPQ loop of HsGrx5 by the TG loop of ScGrx7 and both features in a combined HsGrx5 mutant. Residue Cys122 of HsGrx5, which is part of a moderately conserved GGC-motif in the proximity of the reaction center^4^, was replaced by serine to avoid unwanted side reactions^26,42,43^. The three mutants HsGrx5^RR^, HsGrx5^loop^, and HsGrx5^RR+loop^ were subsequently compared with wild-type HsGrx5 (Fig. 6, Supplementary Figs. 1 and 13–16, Supplementary Tables 7–9). The insertion of the elongated active-site loop in ScGrx7^loop^ decreased the catalytic efficiencies and reciprocal Dalziel coefficients for GSSCys and GSH by more than two orders of magnitude. In contrast, replacement of the RR-motif in ScGrx7^WP^ predominantly affected the catalytic efficiency and reciprocal Dalziel coefficient for GSSCys, resulting in a rate-limiting oxidative half-reaction in accordance with the results from Fig. 5. The oxidative half-reaction became also rate-limiting for ScGrx7^WP+loop^, which had an even lower activity than ScGrx7^loop^. Freshly purified recombinant wild-type HsGrx5 contained an iron–sulfur cluster (as indicated by its brown color and absorbance maxima at 321, 412, and 455 nm) as reported previously^37,38^. For activity measurements, the iron–sulfur cluster was removed by EDTA and subsequent gel filtration chromatography. In contrast, recombinant HsGrx5^RR^, HsGrx5^loop^, and HsGrx5^RR+loop^ were colorless, suggesting that the WP-motif and the elongated active-site loop are both crucial for iron–sulfur cluster binding. Shortening the active-site loop in HsGrx5^loop^ activated the protein, resulting in reciprocal Dalziel coefficients for GSSCys and GSH around 2 × 10^3^ M^−1^ s^−1^ and 5 × 10^3^ M^−1^ s^−1^, respectively. The activities of the other HsGrx5 constructs were too low (≤10^2^ M^−1^ s^−1^) to determine reliable kinetic constants. The intermediate activity of HsGrx5^loop^ exemplifies the necessity of a combination of structural features for the enzymatic activity of class II Grx. In summary, while ScGrx7 is inactivated by the elongated active-site loop of HsGrx5 but not by its WP-motif, HsGrx5 can be transformed into a moderately active enzyme by shortening its elongated active-site loop but not by replacing its WP-motif. The length of the active-site loop is therefore a determinant structural difference between both Grx classes.

### Intracellular mechanistic assessment of ScGrx7 mutants

Fusion constructs between Grx and a redox-sensitive yellow fluorescent protein have previously been used to gain mechansitic insight into glutaredoxin catalysis in vitro^18^, as well as to dynamically monitor the redox state of the intracellular glutathione pool^44,45^. Recently, we successfully established fusion constructs between redox-sensitive green-fluorescent protein 2 (roGFP2) and peroxidases for monitoring their catalytic mechanism and inactivation in living cells^46,47^. We thus asked if roGFP2 fusion constructs could also be adapted for the noninvasive assessment of Grx structure–function relationships inside living cells. To this end, we sought to establish a yeast-based system to permit the rapid screening of roGFP2–Grx constructs.

**Fig. 7:**
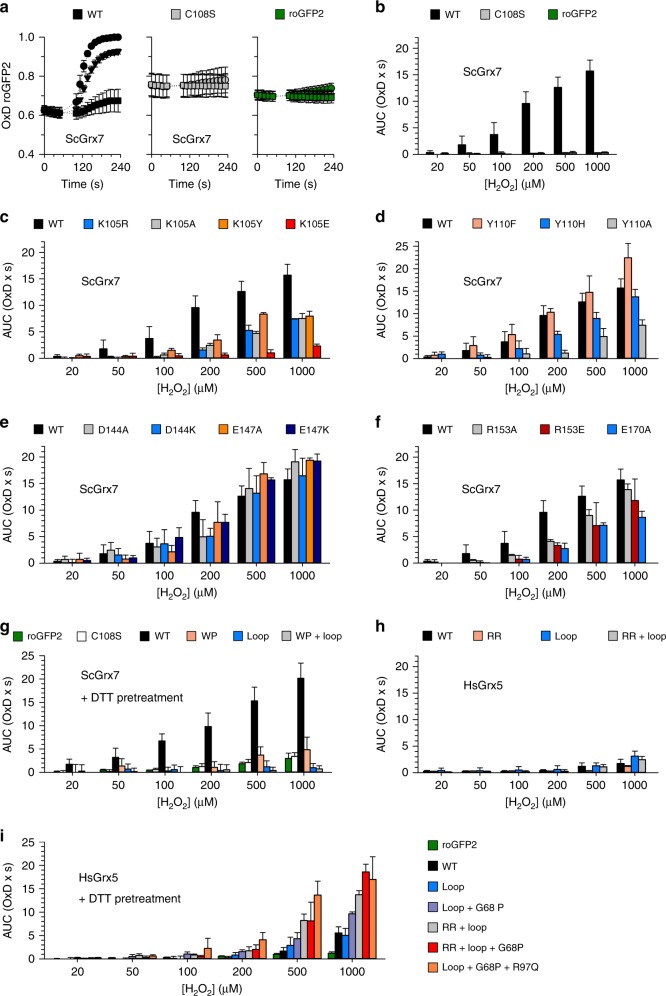
Noninvasive intracellular assessment of Grx structure-function relationships using roGFP2. **a** Time-dependent ratiometric degree of roGFP2 oxidation (OxD) for yeast cells with genetically encoded fusion constructs between roGFP2 and ScGrx7. The results for bolus treatments with 1 mM (circles), 0.2 mM (triangles), and 0.05 mM (squares) H_2_O_2_ are shown. Cells with wild-type roGFP2–ScGrx7 (WT) responded rapidly in contrast to the active-site mutant roGFP-ScGrx7^C108S^ (C108S) and roGFP2 alone (roGFP) which served as negative controls. **b** Integrated dose–response curves from panel (**a**). The area under the OxD curves (AUC) was determined for the first 48 s following the addition of H_2_O_2_. **c**, **d** Integrated dose–response curves for ScGrx7 mutants of residues Lys105 and Tyr110. These residues flank the active-site cysteine residue and the glutathione moiety in glutathionylated Grx. **e**, **f** Integrated dose–response curves for ScGrx7 mutants of residues r_4_, r_5_, r_7_, and r_8_ from Fig. 1. **g**–**i** Integrated dose–response curves for ScGrx7 and HsGrx5 interconversion mutants under standard conditions (panel **h**) or after pretreatment and subsequent washout of DTT (panels **g** and **i**). RoGFP2 alone (roGFP2) as well fusion constructs with inactive ScGrx7^C108S^ (C108S) or wild-type ScGrx7 (WT) served as negative and positive controls. All experiments were repeated at least three times and data were reported as mean AUCs with error bars representing the standard deviation. Source data are provided as a Source Data file. Statistical analyses and *P* values are listed in Supplementary Table 12.

Equilibration of the roGFP2 dithiol/disulfide redox couple with the 2GSH/GSSG redox couple depends upon Grx-mediated catalysis. Therefore, by monitoring the kinetics of roGFP2 oxidation in response to perturbation of the cytosolic glutathione pool we hoped to be able to observe the impact of specific mutations on glutaredoxin activity. Our assay required that we could readily perturb the cytosolic glutathione pool by the addition of exogenous oxidants and that roGFP2 oxidation is specific for the genetically fused glutaredoxin. To fulfill both of these requirements we generated a yeast strain which lacked the genes encoding glutathione reductase and the two enzymatically active cytosolic Grx (Δ*glr1* Δ*grx1* Δ*grx2*). This strain was complemented with cytosolic fusion constructs between roGFP2 and various Grx mutants as described previously^45^. Fusion constructs with wild-type ScGrx7 or its redox-inactive mutant C108S served as a positive and negative control, respectively. Wild-type roGFP2–ScGrx7 was found to be ~60% oxidized at steady state and readily responsive, in a concentration-dependent manner, to the exogenous addition of H_2_O_2_ at concentrations ranging from 0.02 to 1 mM. In contrast, both roGFP2–ScGrx7^C108S^ and unfused roGFP2 were about 70–80% oxidized at steady state and almost completely unresponsive to exogenous H_2_O_2_ (Fig. 7a). To simplify further analyses, we developed a standardized measure of probe response. First, we substracted the baseline of an untreated probe response (0 µM H_2_O_2_) from all H_2_O_2_-treated probe responses. Secondly, we then calculated the integrated area under the curve (AUC) for the first 48 s of the “corrected” probe responses. We subseqently plotted the AUC against the H_2_O_2_ concentration for all constructs tested (Fig. 7b).

The intracellular roGFP2 responses of the ScGrx7 fusion constructs correlated very well with the in vitro data for the oxidative half-reaction, suggesting that GSSG is rapidly sensed as glutathionylated Grx, which subsequently glutathionylates roGFP2 so that it can form an intramolecular disulfide bond. Strongest effects were observed for the charge inversion mutant K105E and for Y110A, followed by intermediate effects for mutants K105R/A/Y and Y110H (Fig. 7c, d). Mutant Y110F appeared to be slightly more active than wild-type ScGrx7 in accordance with the in vitro data. No significant differences were detected between mutants D144A/K or E147A/K and wild-type ScGrx7 (Fig. 7e) in accordance with the predominantly unaffected reaction rates of these mutants with GSSCys. In contrast, lowered roGFP2 responses were detected for fusion constructs with mutants R153A/E and the mutant E170A, which served as a reference for the glutathione-scaffold site^12^ (Fig. 7f). These results further support our interpretation that the altered roGFP2 responses reflect changes during the oxidative half-reaction of the ScGrx7 mutants. In other words, the transfer of oxidation from ScGrx7 to the fused roGFP2 moiety does not appear to be rate-limiting, rather, the rate of oxidation of ScGrx7 by GSSG appears to dictate the kinetics of roGFP2 oxidation.

### Intracellular assessment of Grx interconversion mutants

We also addressed the interconversion of enzymatically active and inactive Grx in yeast using roGFP2-based fusion constructs. In the fusion construct between roGFP2 and ScGrx7^WP^, the roGFP2 moiety was found to be almost fully oxidized at steady state. RoGFP2 was ~80% oxidized at steady state in fusion constructs involving ScGrx7^loop^ or ScGrx7^WP+loop^. In all three constructs roGFP2 oxidation appeared to be robust against further oxidation upon exogenous H_2_O_2_ addition, however, the high steady-state oxidation limited the possibility for further roGFP2 oxidation and confounded interpretation of oxidation kinetics (Supplementary Fig. 17). Therefore, to gain further insight into the functionality of these ScGrx7 constructs we first pretreated the cells with 50 mM DTT to fully reduce roGFP2, followed by a washing step to remove DTT, and then subsequently monitored kinetics of roGFP2 oxidation upon H_2_O_2_ addition. In all constructs, around 10–20% of roGFP2 molecules were oxidized after the DTT pretreatment and subsequent washing steps. The exception was wild-type roGFP2–ScGrx7, which was ~40% oxidized. Importantly, after addition of exogenous H_2_O_2_, we still observed very little response of all constructs, except wild-type roGFP2–ScGrx7, which responded rapidly (Fig. 7g). Again, except for ScGrx7^WP^, the loss of function of the interconversion mutants ScGrx7^loop^ and ScGrx7^WP+loop^ in yeast correlated very well with the in vitro data.

We next asked about the response of fusion constructs between roGFP2 and HsGrx5, HsGrx5^RR^, HsGrx5^loop^, or HsGrx5^RR+loop^. In all cases, the constructs were poorly active and required high H_2_O_2_ concentrations to detect a roGFP2 oxidation response under standard conditions without DTT pretreatment. However, replacement of the elongated GTPEQPQ loop of HsGrx5 by the TG loop of ScGrx7 in HsGrx5^loop^ resulted in an up to twofold increase of the AUC as compared with wild-type roGFP2–HsGrx5 (Fig. 7h). Thus, replacement of the loop of HsGrx5 slightly increased its activity, though neither the loop nor the WP-motif (alone or in combination) is enough to impart a high oxidoreductase activity to HsGrx5 using roGFP2 as a substrate. We therefore screened for additional HsGrx5 mutations that might increase the oxidoreductase activity using our more sensitive DTT treatment and washout protocol. Under these conditions, we were able to identify positive additive effects for the short TG loop in combination with a replacement of residue Gly68 in the CGFS motif by proline, the replacement of the WP-motif by the RR-motif, and/or replacement of Arg97 (residue r_3_ in Fig. 1) by the conserved glutamine residue in helix 3 (Fig. 7i). HsGrx5 triple mutants of these protein areas were, at the highest H_2_O_2_ concentrations, almost as active as ScGrx7.

In summary, roGFP2 can be used for the noninvasive intracellular assessment of Grx structure–function relationships yielding similar patterns for glutathione-scaffold site mutants in vitro and in yeast. Furthermore, the intracellular roGFP2 assay can be used to rapidly screen a set of potential loss- or gain-of-functions mutants of a variety of Grx isoforms. A screen for HsGrx5 gain-of-function mutants revealed that protein areas (i)–(iv) from Fig. 1 synergistically contribute to the oxidoreductase activity of Grx.

### Simulation of the interaction between GS^−^ and ScGrx7-SSG

**Fig. 8:**
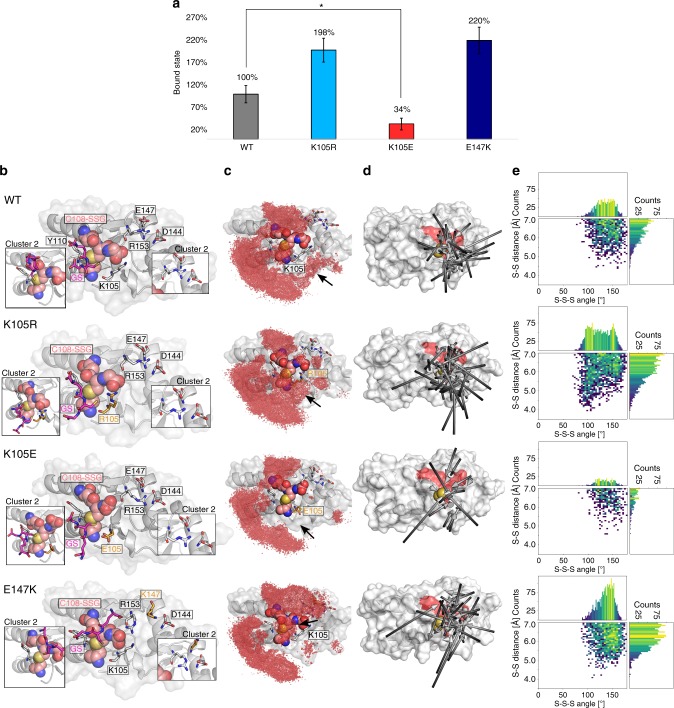
Molecular dynamics simulations of GS^−^ binding to glutathionylated ScGrx7. **a** Fraction of bound states with the value for wild-type ScGrx7 (WT) normalized to 100%. Error bars show the standard error of the mean over four replications for each system. Datasets were tested with a two-tailed *t* test assuming unequal variances; *: null-hypothesis of equal means can be rejected with *P* < 0.05. For WT and K105R *P* = 0.13; for WT and E147K *P* = 0.15 (see also Supplementary Table 13). **b** For each of the systems of the WT and the variants K105R, K105E, and E147K, bound states were clustered with respect to the structural deviation of the active site and the closest GS^−^ from the solution. The structure representing the most populated cluster is shown in the middle and important areas of the structure representing the second most populated cluster (cluster 2) are shown in the boxes next to it. The covalently bound glutathione moiety at Cys108 is depicted in salmon, the freely diffusing GS^−^ in pink. Residues marked in orange show the mutated residues in the variants. All residues with labels were also studied experimentally. **c** The occupation density of the diffusing GS^−^ is shown as a red grid on the structure of each ScGrx7 variant. Bound states were combined across replications for this analysis, and the threshold of the density grids is the same for all variants. **d** Arrows show the binding pathways of GS^−^. For each binding event, an arrow shows the linearized trajectory of the GS^−^ molecule right before the binding event going from black (start of the pathway) to gray (bound). The active site is shown as a red surface and the sulfur atoms of the mixed disulfide bond are shown in yellow. **e** Histograms show the distance between the sulfur atom of GS^−^ and the center of mass of the sulfur atoms of Cys108-SSG (“S–S-distance”) and the angle created by the three sulfur atoms (“S–S–S angle”) over all four replications each. The color of the bins ranges from dark blue (lowest populated bin) over green to yellow (highest populated bin). See Supplementary Table 14 for results of the correlation analysis.

We previously showed that conserved residue Lys105 of ScGrx7 (*K*_a_ in Fig. 1) serves as a glutathione activator for the reductive half-reaction between GSH and the glutathionylated enzyme^12^. To gain insights into the first step of the reductive half-reaction of ScGrx7 at an atomistic level and to identify the residues that form the glutathione-activator site, we performed four replications of molecular dynamics simulations each of wild-type ScGrx7 and the variants K105R, K105E, and E147K. We focused only on the substrate access of the reductive half-reaction by simulating the binding of deprotonated glutathione (GS^−^) in the presence of oxidized ScGrx7, i.e., ScGrx7 that is glutathionylated at Cys108 (Fig. 8). We defined binding events of GS^−^ with a distance cutoff of 5.5 Å between the sulfur atom of one of the freely diffusing GS^−^ molecules and the center of mass of the sulfur atoms of the disulfide bond between the active-site cysteine and the covalently bound glutathione moiety. Overall, the protein structure of ScGrx7 in all simulations was structurally stable and exhibited no major movements of secondary structure elements (Supplementary Figs. 18 and 19). With respect to the fraction of bound GS^−^ states, we observed a ~2-fold increase for K105R and a significant decrease to ~34% for K105E compared to wild-type ScGrx7 (Fig. 8a). For the E147K variant, the fraction of bound states increased by 2.2-fold. These result are in good agreement with those of substitutions of Lys105 by arginine or glutamate that were previously shown to accelerate or decelerate the reductive half-reaction in vitro^12^.

To understand these differences at the structural level, we only considered snapshots that fulfill our distance-based criterion for GS^−^ binding in the following analyses. The representative conformations of the first two clusters of binding poses revealed clear differences in the active sites and binding poses of GS^−^ among the different variants (Fig. 8b). In addition, the occupation density of glutathione (Fig. 8c), its binding pathway (Fig. 8d), and the distance and angular distributions of the encounter complex between GS^−^ and Cys108-SSG (Fig. 8e) were analyzed. The side chain of residue 105 interacts with the glycine carboxyl group of the covalently bound glutathione in wild-type ScGrx7 and the K105R variant, but with the backbone of Cys108 in the K105E variant (Fig. 8b). This conformational change is accompanied by a rotation of the glycine carboxyl group away from the active site toward the solution in the K105E variant. As a consequence, in the binding pose of GS^−^ in the K105E variant, the glycyl moiety of GS^−^ is positioned near Tyr110, and the γ-glutamyl moiety points away from the protein surface, in contrast to wild-type ScGrx7 and the K105R variant. These changes are reflected in differences in the occupation densities of GS^−^ (Fig. 8c) in that in the K105R variant a patch above residue 105 and toward Cys108-SSG is more frequently occupied than in wild-type ScGrx7, but less frequently occupied in the K105E variant.

Substitution of Glu147 with lysine leads to a rearrangement of the side chain of Arg153 toward the glycine carboxyl group of the covalently bound glutathione, in accordance with a changed binding pose of GS^−^ where the molecule is rotated by about 90° so that either the carboxyl group of its γ-glutamyl moiety or its glycyl moiety interacts with the Arg153 guanidinium group (Fig. 8b). This alternative conformation of Arg153 is stable over the whole simulation time for E147K, while it is less frequently sampled for the other variants (Supplementary Fig. 20). As a consequence, the E147K variant exhibits a different pattern of occupation density of GS^−^ across the active site, with the patch of higher occupation across Cys108-SSG now being shifted towards the glutathione moiety and Arg153 (Fig. 8c).

The analysis of binding paths revealed that GS^−^ mostly directly approaches Cys108-SSG from the solvent rather than it exploring the surface near the active site (Fig. 8d). In line with this, there is no single most preferred binding pathway. However, GS^−^ only rarely approaches from the direction of Tyr110, while pathways crossing Lys105 and helix 3 below Glu147 and Asp144 are more common. A contact analysis for GS^−^ revealed several patches, for example, including the covalently bound glutathione moiety and residues Lys105, Thr106, and Gly107 from protein area (iv) in Fig. 1 (Supplementary Fig. 21, Supplementary Table 15). The distance between the sulfur atom of GS^−^ and the center of mass of the sulfur atoms of Cys108-SSG (“S–S distance”) and the angle created by the three sulfur atoms (“S–S–S angle”) (Fig. 8e) are uncorrelated for wild-type ScGrx7 and the K105E variant and only weakly correlated for the E147K variant (*R*^2^ = 0.08, *P* = 0.001). By contrast, both parameters are fairly correlated in the K105R variant (*R*^2^ = 0.42, *P* < 0.001) (Supplementary Table 14).

In summary, we were able to relate changes in rate constants of the reductive half-reaction upon substitutions in ScGrx7 to differential GS^−^ access to the active site and the covalently bound glutathione in the molecular dynamics simulations. The observed higher fraction of bound states of GS^−^ to the K105R and E147K variants is caused by conformational changes in the binding sites, which lead to differential population of specific binding site regions. In the K105R variant, furthermore, there is a fair correlation between S–S distances and S–S–S angles, which may impact the turnover rate of the reaction. In the E147K variant, by contrast, the fraction of bound states is indirectly increased by changing the conformation of the Arg153 side chain, leading to an increase in overall positive charge at the active site.

### Simulation of the interaction between GS^−^ and HsGrx5-SSG

**Fig. 9:**
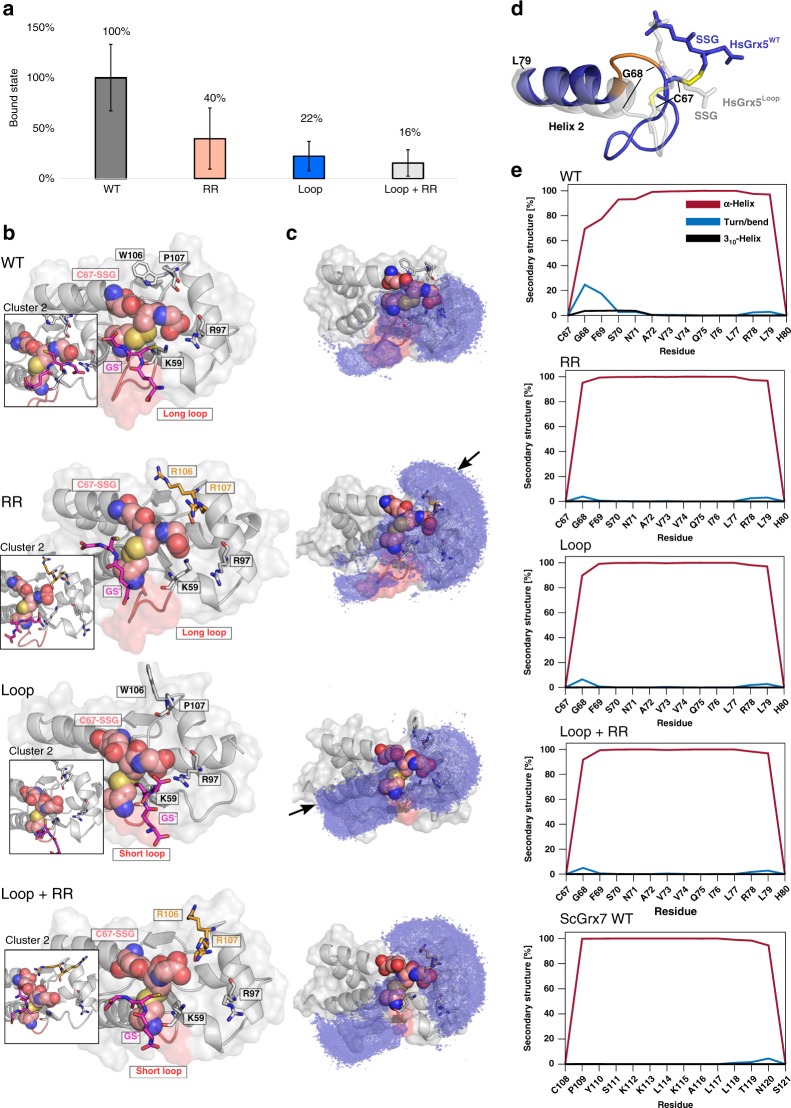
GS^−^ binding to and conformational changes of glutathionylated HsGrx5 variants. **a** Fraction of bound states with the value for wild-type HsGrx5 (WT) normalized to 100%. Error bars show the standard error of the mean over four replications for each system. Datasets were tested with a two-tailed *t* test assuming unequal variances; null-hypothesis of equal means could not be rejected with *P* < 0.05. For WT and HsGrx5^RR^ *P* = 0.17; for WT and HsGrx5^loop^ *P* = 0.10; for WT and HsGrx5^RR+loop^ *P* = 0.08. **b** For each of the systems of the WT and the variants HsGrx5^RR^, HsGrx5^loop^, and HsGrx5^RR+loop^, bound states were clustered with respect to the structural deviation of the active site and the closest GS^−^ from the solution. The structure representing the most populated cluster is shown in the middle, and important areas of the structure representing the second most populated cluster are shown in the boxes next to it. The covalently bound glutathione moiety at Cys67 is depicted in salmon, the freely diffusing GS^−^ in pink. Residues marked in orange show the mutated residues in the variants; the active site loop is colored red. Residue are numbered always according to the WT sequence. **c** The occupation density of the diffusing GS^−^ is shown as a blue grid on the structure of each HsGrx5 variant. Bound states were combined across replications for this analysis, and the threshold of the density grids is the same for all variants. Arrows show the additional patterns emerging for the RR and loop variants compared to the WT, respectively. **d** Partial unfolding of helix 2 (residues marked orange) was observed in one of the replications of HsGrx5 WT (blue), HsGrx5^loop^ is shown for comparison of the helical conformation (gray). **e** Residue-wise fraction of secondary structure content averaged over the four MD replications of the HsGrx5 variants and the ScGrx7 WT for helix 2. Only relevant secondary structure types are shown: α-helix in red, turn or bend in blue, and 3_10_-helix in black. Adjacent residues Cys67/Cys108 and His80/Ser121 have a coil conformation.

We performed molecular dynamics simulations and subsequent analysis of wild-type HsGrx5 (WT) as described for ScGrx7, i.e., with a glutathionylated Cys67 and freely diffusing GS^−^ in the solution. We also chose the variants HsGrx5^RR^, HsGrx5^loop^, and HsGrx5^RR+loop^ for comparison with the in vitro and in vivo experiments. The fraction of bound GS^−^ states was lower for all variants of HsGrx5 than the WT (Fig. 9a) although not statistically significantly. Compared to ScGrx7 (Fig. 8b), clustered binding poses of GS^–^ at the active site exhibit a larger heterogeneity (Fig. 9b), and GS^−^ is oriented more towards conserved Lys59. Notably, residue Arg97 is able to interact with the glycine carboxyl group of Cys67-SSG in some of the clusters. Concordant with the diverse binding poses, the occupation density for HsGrx5 (Fig. 9c) shows different patches across the surface compared tor ScGrx7, with no sampling between helix 2 and helix 4 but primarily across helix 3 and its N-terminal loop. In HsGrx5^RR^ and HsGrx5^RR+loop^, the density is markedly extended across and around the surface of the RR motif, while this area is rarely sampled in the cases of WT and HsGrx5^loop^. In HsGrx5^loop^ and HsGrx5^RR+loop^, the occupation density extends along helix 2.

A comparison of EcGrx4 structures with and without iron–sulfur cluster^34,36^ revealed conformational changes of the N-terminal part of helix 2 and the following elongated loop (Fig. 1c). Secondary structure analysis of helix 2 across all simulations of HsGRx5 variants revealed conformational changes in terms of an increased propensity of turn/bend or 3_10_-helix formation in that region only for the WT (Fig. 9d, e). This conformational change was seen only in one of the four replications (not shown), concordant with the slow unfolding kinetics of helices compared to our simulation time scales^48^. Only short-lived (~20 ns) conformational changes of Gly68 were observed for the other variants of HsGrx5, while the corresponding Pro109 residue in ScGrx7 remained α-helical throughout the simulations (Fig. 9e). A marked difference between ScGrx7 and HsGrx5 is the interaction between the conserved lysine residue at the active site and Cys-SSG. In all HsGrx5 variants, Lys59 forms mostly transient interactions to the glycine carboxyl group of Cys-SSG (Supplementary Fig. 22a), whereas Lys105 in ScGrx7 always forms a stable salt bridge in the WT and E147K variant (Supplementary Fig. 22a, b). With respect to differences among HsGrx5 variants, in HsGrx5^loop^ and HsGrx5^RR+loop^, Lys59 is consistently closer to the disulfide with a distance of ~6 Å than in WT and HsGrx5^RR^, where distances ~9–11 Å are sampled as well (Supplementary Fig. 22c, d). In summary, the increased activity of HsGrx5^loop^ and HsGrx5^loop+RR^ in vitro correlates with increased occupation densities of GS^−^ near the active site. The increased occupation may result from an improved Lys59-dependent attraction of GS^−^ from the solution because the residue is consistently closer to the active-site disulfide.

## Discussion

We established redox-sensitive GFP2 as a tool for the noninvasive intracellular assessment of Grx structure–function relationships yielding similar patterns for glutathione-scaffold site mutants in vitro and in yeast. This novel technique can now be used, for example, to perform initial structure-function analyses by rapidly screening multiple Grx mutants or isoforms from a variety of species without needing to go to the initial effort of purifying recombinant proteins. Furthermore, in combination with classic yeast genetics, the method might be also adopted to screen for gain- or loss-of-function mutants as well as specifc protein–protein interactions or post-translational modifications.

In our roGFP2 assays we choose to use H_2_O_2_ to initiate GSSG formation. Whilst yeast do not harbor any bona fide glutathione peroxidase, it was recently shown that GSH and/or Grx can reduce both 1-Cys and typical 2-Cys peroxiredoxins thereby leading to GSSG production^42,46,47,49,50^. Furthermore, GSSG was shown to readily accumulate in Δ*glr1* cells following H_2_O_2_ treatment^45^. We used Δ*glr1*Δ*grx1*Δ*grx2* cells for our assay, which lack any endogenous cytosolic glutaredoxin activity. Thus, the expressed roGFP2–Grx fusions will likely play a role in cytosolic GSSG production in our assays following H_2_O_2_ treatment. It is therefore possible that Grx mutants with impaired activity will affect GSSG production as well as roGFP2 oxidation. Consequently, with this assay, it is not possible to strictly separate the impact of Grx mutants on the capacity to reduce glutathionylated peroxiredoxins on the one hand and the ability to transfer this oxidation to roGFP2 on the other hand. Nonetheless, we consider that the consistent and robust correlation between our in vitro assays and in cellulo roGFP2 assays fully supports the conclusion that roGFP2-based assays allow rapid assessment of glutaredoxin activity, mechanism and structure–function relationships in living cells.

Which protein areas are crucial for Grx catalysis? All four protein areas in Fig. 1 directly or indirectly affect the reactivity of Grx with GSSR and GSH. In addition to residue Glu170, which was characterized previously^12^, our kinetic analyses reveal that residues Tyr110, Asp144, Arg152, and Arg153 (r_1_, r_4_, r_6_, and r_7_ in Fig. 1) form parts of the glutathione-scaffold site. Based on the effect of alanine mutations on the rate constants for the oxidative half-reaction, the relevance of these glutathione-scaffold site residues is ranked as follows: Tyr110 > Arg152/Arg153 > Arg153 > Glu170 > Asp144. Furthermore, the roGFP2 assay with the HsGrx5 gain-of-function mutants confirms an important role for the active-site proline residue and for the conserved glutamine residue in helix 3 (r_3_ in Fig. 1). Regarding protein area (i) in Fig. 1, the hydroxyl group of Tyr110 is dispensable for catalysis and its removal could even accelerate the reaction with GSSCys. These results are in accordance with previous activity measurements of poplar Grx mutants at a single substrate concentration^51^. The conservation of the tyrosine residue might point towards a post-translational modification or the recognition of a specialized substrate. While molecular dynamics simulations suggest a direct role of protein area (i) for the interaction with GS^−^, replacement of Tyr110 (or Arg153) can also indirectly affect the reductive half-reaction, as observed for mutants Y110A and R153E. Thus, positioning of the glutathione moiety in glutathionylated Grx is crucial for the reactivity with GSH and significantly contributes to the glutathione-activator site, although it still remains puzzling how Grx use GSH much more eficiently than cysteinylglycine or other thiols^22,52^.

The side chains of the conserved active-site residues Lys105 and Tyr110 do not affect the cysteine thiol p*K*_a_ value of free ScGrx7. Even a charge inversion of Lys105, which was shown to slow down the reaction with GSSCys by two orders of magnitude^12^, has only moderate effects on the cysteine protonation state. These findings correspond well with experimental data for human Grx1, but differ from studies on the inverse D35Y mutant of yeast ScGrx8 as well as calculations for Lys19 of human Grx1 and Lys8 of NrdH-redoxin from *C. glutamicum*^33,40,41^. Since Lys105 and Tyr110 do not alter the thiol p*K*_a_ value of free ScGrx7, we suggest that these flanking residues stabilize the conformation of the free and glutathionylated enzyme and/or its negatively charged transition states. Further studies are needed to unravel these nonexclusive contributions in addition to the relevance of Lys105 as a GSH activator and potential stabilizer for the thiolate leaving group of the first product^12^.

Regarding protein area (ii) in Fig. 1, helix 3 not only contributes to the glutathione-scaffold site but also to the charge-dependent recruitment of GSH, as exemplified by the molecular dynamics simulations and the accelerated reductive half-reaction of the gain-of-function mutants D144K and E147K. The results confirm that geometric and electrostatic complementarity are both crucial for glutathione catalysis^4,12,13,53^. A minus-plus-plus charge distribution along the side chains of helix 3 is frequently found in enzymatically inactive Grx in contrast to the much more variable charge distribution in active Grx (Fig. 1d). The gain-of-function mutants D144K and E147K refute the hypothesis that additional positive charges in helix 3 prevent a productive interaction with GSH. The potentially missed option for ScGrx7 optimization by a simple point mutation in vivo might point toward alternative helix 3-dependent physiological substrate interactions, as previously demonstrated for the complementary surfaces between EcGrx1 and a peptide from ribonucleotide reductase^28^.

Regarding protein area (iii) in Fig. 1, replacement of Arg153 or the introduction of the WP-motif decelerates the oxidative but not the reductive half-reaction of ScGrx7 in vitro and in yeast. Thus, the WP-motif does not prevent the interaction with GSH but might stabilize the loop between helix 3 and strand 3 and decelerate the unwanted glutathionylation of enzymatically inactive Grx. This interpretation is in accordance with the detected gain-of-function of selected HsGrx5 mutants in the intracellular roGFP2 assay. The relevance of this loop as a crucial part of the glutathione-scaffold site is also supported by previous studies on a conserved TV-motif and its replacement in ScGrx8 (Fig. 1d)^33^. An additional function of the WP-motif might be to facilitate or to block the interaction of enzymatically inactive Grx with a specific protein. Substrate- and conformation-dependent altered reduction and oxidation kinetics might explain why ScGrx7^WP^ is active with GSSCys in vitro whereas bulky roGFP2 is predominantly oxidized in yeast. Likewise, HsGrx5 and *Arabidopsis thaliana* GrxS15 cannot efficiently reduce roGFP2 in yeast and in vitro, respectively^12^. An inactivating WP-motif also makes sense from a physiological perspective, because it might allow the stabilization of a modified sensor and/or avoid the accumulation of trapped Grx-SS-protein species in the absence of a resolving cysteine^7,11,13^. For example, ScGrx3 and ScGrx4 both have a WP-motif and were shown to deglutathionylate very slowly the histone deacetylase Sir2 in a redox-dependent signaling cascade^54^.

How is the Grx-dependent synthesis or sensing of iron–sulfur clusters kinetically uncoupled from redox catalysis at millimolar GSH concentrations^12,13^, in particular, taking into account that class II Grx can be glutathionylated in vitro^26,55,56^? The active-site loop in protein area (iv) in Fig. 1 seems to affect an important GS^−^ interaction pathway according to molecular dynamics simulations. Furthermore, modification of this loop had the strongest effect on the oxidative and the reductive half-reaction of ScGrx7 and HsGrx5 and allowed a partial interconversion between enzymatically active and inactive Grx. The variable loop before the active-site cysteine residue is therefore a determinant structural difference between both Grx classes and seems to act as an on/off switch. Nevertheless, our data reveal that protein areas (i)–(iv) together determine and fine-tune the oxidative and reductive half-reaction of Grx. An appropriate loop conformation near the active site is necessary to convert an enzymatically inactive Grx to an active one, but such a conformation alone is not sufficient, and additional replacements, for example, of Gly68 and/or Arg97 in HsGrx5 can further increase the enzymatic activity. A kinetic uncoupling mechanism because of a loop-dependent on/off switch is supported by the NMR solution structures of EcGrx4 (Fig. 1c)^34^. Helix 2 in the apoprotein is partially unfolded so that the active-site cysteine and lysine residue are repositioned and point away from the glutathione-scaffold site. This protein conformation obviously has to be enzymatically inactive. In contrast, EcGrx4 adopts a potentially functional conformation in the presence of the iron–sulfur cluster^36^, however, the cysteine residue of the holoprotein is now blocked and therefore remains enzymatically inactive. If we assume that the conformational change in class II Grx is triggered or stabilized by the iron–sulfur cluster, we can explain the enzymatic inactivity, because the effective concentration of free enzyme in the active conformation is too low. Conformational changes at the N-terminus of helix 2 and the active-site loop are less pronounced in the apo- and holoprotein NMR solution structures of HsGrx5, however, the local unfolding of helix 2 and the crucial repositioning of the active-site cysteine residue in the apoprotein (PDB entry 2MMZ)^38^ are similar to EcGrx4. Furthermore, while our molecular dynamics simulations did not reveal an increase of bound GS^−^ for loop mutants of HsGrx5, a conformational transition from the proposed active conformation to a presumably inactive state with a partially unfolded helix 2 and rearrangement of the Cys-SSG disulfide was observed for wild-type HsGrx5. In contrast, the N-terminal part of helix 2 of ScGrx7 always remained α-helical during the simulations. Subtle conformational changes around the active-site proline, serine or glycine residue in protein area (i) are also in agreement with structural and kinetic data on HsGrx1, HsGrx2, ScGrx1, ScGrx2, and ScGrx6-8^4,11,19,29,40,57^. For example, ScGrx6 with its CSYS-motif has a ~40 times lower $k_{{\mathrm{cat}}}^{{\mathrm{app}}}$ value than CPYS-containing ScGrx7^19^. In summary, we propose an active-site loop-dependent conformational switch that parks the apoprotein of class II Grx in an enzymatically inactive state when no iron–sulfur clusters are present. This conformational switch, in addition to combined structural variations around the substrate interaction sites, kinetically uncouple the Grx-dependent synthesis or sensing of iron–sulfur clusters from redox catalysis at millimolar GSH concentrations.

In conclusion, we established and applied a roGFP2-based assay to rapidly screen for gain- or loss-of-function mutants of Grx isoforms in yeast, quantified the relevance and contribution of four crucial protein areas for the oxidative and reductive half-reaction of Grx catalyis, show that the flanking lysine and tyrosine residue do not affect the thiol p*K*_a_ value of the active-site cysteine residue but rather stabilize the transition states, and propose an active-site loop-dependent conformational on/off switch that inactivates class II Grx in the absence of iron–sulfur clusters.

## Methods

### Materials

GSH, GSSG, diamide, H_2_O_2_, and yeast glutathione reductase (ScGR) were from Sigma-Aldrich, HEDS was obtained from Alfa Aesar, GSSCys from Toronto Research Chemicals, DTT from AppliChem, and NADPH was from Gerbu. Polymerase chain reaction (PCR) primers were purchased from Metabion.

### Site-directed mutagenesis, gene synthesis, and cloning

Point mutations were introduced by PCR with *Pfu* polymerase (Promega) using the mutagenesis primers listed in Supplementary Table 10 and the double stop-codon construct of pQE30/*SCGRX7* (ref. ^11^) as template. Following the digestion of the methylated template DNA by *Dpn*I (NEB), plasmids were transformed into chemically competent *E. coli* XL1-Blue cells. Correct mutations and sequences were confirmed for all constructs by sequencing both strands (SEQ-IT). Codon- and mRNA structure-optimized genes *SCGRX7*^*WP*^, *SCGRX7*^*loop*^, *SCGRX7*^*WP+loop*^, *HSGRX5*^*C122S*^, *HSGRX5*^*RR*^, *HSGRX5*^*loop*^, and *HSGRX5*^*RR+loop*^ were synthesized (Genscript) and either subcloned into the *Eco*RI and *Xho*I restriction sites of p416TEF/*roGFP2* (ref. ^45^) for roGFP2 measurements in yeast or PCR-amplified using the primers in Supplementary Table 10 and subcloned into the *Bam*HI and *Hin*dIII restriction sites of pQE30 for heterologous expression in *E. coli*. Please note that all *HSGRX5* constructs encode C122S mutants that lack the mitochondrial presequence and start with residue Ala32.

### Heterologous expression and protein purification

*E. coli* strain XL1-Blue was transformed with the according pQE30 plasmid for the expression of wild-type and mutant *SCGRX7* and *HSGRX5*^*C122S*^. Recombinant N-terminally MRGS(H)_6_-tagged wild-type and mutant ScGrx7 and HsGrx5^C122S^ (without their N-terminal targeting sequences^19^) were purified after lysozyme treatment and sonication by Ni-NTA affinity chromatography using an elution buffer containing 200 mM imidazole, 300 mM NaCl, and 50 mM sodium phosphate, pH 8.0^11,19,25,42^.

### GSSCys and HEDS oxidoreductase assays

Steady-state kinetics of wild-type and mutant ScGrx7 and HsGrx5^C122S^ in the GSSCys and HEDS assays were determined spectrophotometrically by monitoring the consumption of NADPH at 340 nm and 25 °C using a thermostated Jasco V-650 UV/vis spectrophotometer^11,19,25^. Fresh stock solutions of NADPH, GSH, GR, and GSSCys or HEDS were prepared in assay buffer containing 0.1 M Tris/HCl, 1 mM EDTA, pH 8.0 before each experiment. Both assays were performed with 0.1 mM NADPH and 1 U/ml ScGR. The following final protein concentrations were used for Y110F/H/A (5–150 nM), D144A/K (10 nM), E147A/K (10 nM), R153A/E (25–50 nM), ScGrx7^WP^ (20 nM), ScGrx7^loop^ (up to 1.4 µM), ScGrx7^WP+loop^ (up 2.6 µM), HsGrx5^C122S^ (up to 15 µM), HsGrx5^RR^ (up to 12 µM), HsGrx5^loop^ (up to 1.6 µM), and HsGrx5^RR+loop^ (up to 4.0 µM). For the GSSCys assays, GSH was varied between 50 µM and 1.5 mM at fixed GSSCys concentrations (25, 50, 100, and 150 µM). NADPH, GSH, and GR were mixed in assay buffer before Grx was added and a baseline was recorded for 30 s. All GSSCys assays were started by the addition of GSSCys. The absorbance of a reference cuvette containing all components but no Grx was measured in parallel and subtracted from the obtained Grx activity. For the HEDS assays, GSH was varied between 100 µM and 2.0 mM at fixed concentrations of HEDS (0.18, 0.37, 0.55, and 0.74 mM). NADPH, GSH, and HEDS were preincubated in assay buffer for 2 min before GR was added and a baseline was recorded for 30 s. All HEDS assays were started by the addition of enzyme. Kinetic data were analyzed in Excel and SigmaPlot 13 by nonlinear and linear regression according to Michaelis–Menten, Lineweaver–Burk, Eadie–Hofstee, and Hanes theory^11,19,25^.

### Determination of the thiol p*K*_a_ value of ScGrx7

The protocol was modified from Mieyal et al.^58^ and Gallogly et al.^22^ and is based on the pH-dependent alkylation of the cysteine thiolate of ScGrx7 followed by determination of the residual enzyme activity in a standard HEDS assay. Freshly purified protein (0.3 mM) was reduced with a 20-fold molar excess of NaBH_4_ for 2 h on ice following a previous established protocol^42^. Subsequently, 6 µM wild-type or mutant ScGrx7 was incubated for 180 s with 150 µM iodacetamide at 23 °C in a three-buffer system^59^ containing 100 mM KCl, 50 mM potassium acetate, 50 mM MES, and 100 mM Tris at pH values between 3.5 and 8.5. A mock control without iodacetamide was incubated in parallel and the activity of this control was used for normalization. Enzyme activities after incubation with or without iodacetamide were determined in standard HEDS assays as described above. The percentage of the normalized residual activity after alkylation was calculated in Excel and plotted in SigmaPlot13. A sigmoidal fit (Hill equation, 4 parameter) was used to determine the p*K*_a_ values of ScGrx7 and the different mutants.

### Yeast strains and growth conditions

All experiments were performed in a YPH499 strain background (*MAT*a *ura3-52 lys2-801_amber ade2-101_ochre trp1-Δ63 his3-Δ200 leu2-Δ1*). Yeast were grown in Hartwell’s complete (HC) medium lacking the appropriate amino acids for plasmid selection with 2% glucose as carbon source.

### Generation of yeast strains

Deletion of the genes encoding glutathione reductase (*GLR1*), glutaredoxin 1 (*GRX1*), and glutaredoxin 2 (*GRX2*) were performed using a standard homologous recombination-based technique. Antibiotic resistance markers were amplified using primers with homologous regions up- and downstream of the gene of interest. PCR products were transformed into yeast cells using a standard lithium acetate-based method.

Gene deletions were confirmed by PCR on chromosomal DNA using primers designed to bind ~200 bp up- and down-stream of the gene of interest. Furthermore, PCR reactions were performed using primers designed to bind up- or down-stream of the gene of interest in combination with primers designed to bind inside the antibiotic resistance maker genes.

### Intracellular roGFP2-based monitoring of Grx activity

RoGFP2 has been engineered to contain two cysteine residues on parallel β-strands adjacent to the GFP chromophore. The two cysteines can form a disulfide bond. The roGFP2 dithiol/disulfide redox couple readily equilibrates with the cellular 2GSH/GSSG redox couple in a manner dependent upon glutaredoxin activity. RoGFP2 exhibits two major fluorescence excitation maxima at ~400 and ~490 nm, with one major emission maximum at ~510 nm. The intensity of the two excitation maxima changes in opposite directions upon the formation of the disulfide bond. Ratiometric fluorescence measurements therefore allow the real-time monitoring of the roGFP2 oxidation state.

YPH499 Δ*glr1*Δ*grx1*Δ*grx2* yeast cells were transformed with p416TEF plasmids for the expression of roGFP2–Grx fusion constructs containing either wild-type or mutant *GRX* variants. Cells were grown at 30 °C in HC media, lacking uracil for plasmid selection, to a *D*_600_ ≈ 3.0. Subsequently, the response of the roGFP2–Grx fusion constructs to exogenous H_2_O_2_, applied at concentrations ranging from 0 to 1000 µM, was monitored^45,60^. Briefly, cells were harvested from the growth media by centrifugation at 800*g,* 3 min, room temperature, and resuspended in 100 mM MES/Tris pH 6.0 buffer to a *D*_600_ ≈ 7.5. Subsequently, 200 µl aliquots of cells suspension were transferred to the wells of a 96-well plate. Control samples were treated with either 100 mM DTT or 20 mM diamide to yield fully reduced and oxidized roGFP2, respectively. These samples allow for determination of the degree of probe oxidation (OxD), according to Eq. (1),

1 $${\mathrm{OxD}}_{roGFP2} = \frac{{(I400{\mathrm{sample}} \ast I480{\mathrm{red}}) - (I400{\mathrm{red}} \ast I480{\mathrm{sample}})}}{{(I400{\mathrm{sample}} \ast I480{\mathrm{red}} - I400{\mathrm{sample}} \ast I480{\mathrm{ox}}) + (I400{\mathrm{ox}} \ast I480{\mathrm{sample}} - I400{\mathrm{red}} \ast I480{\mathrm{sample}})}},$$

where “*I*” represents fluorescence emission following excitation at 400 or 480 nm, for the fully oxidized (ox), fully reduced (red) and experimental samples (sample), respectively.

The 96-well plates were centrifuged at 30*g*, 5 min, room temperature to create loose pellets of yeast cells at the bottom of the wells. Subsequently, H_2_O_2_ was added at the required concentration and the fluorescence response was monitored using a BMG Labtech CLARIOstar fluorescence plate-reader. All experiments were performed at least three times using cells from independent cultures. In addition, pre-reduction experiments were performed for constructs that had a too high steady-state roGFP2 oxidation to perform kinetic analyses. Briefly, cells expressing the relevant constructs were incubated with 50 mM DTT for 5 min, isolated by centrifugation, washed once with 100 mM MES/Tris pH 6.0 buffer, and then treated as described above for all other roGFP2 constructs.

### Data analysis and statistics

RoGFP2 responses were analyzed by calculating the integrated area under the roGFP2 response curves, which had been corrected by subtraction of an untreated sample. The area was determined for the first 48 s following addition of H_2_O_2_. All experiments were repeated at least three times and data were reported as mean AUCs with error bars representing the standard deviation. Statistically significant differences between samples were determined using one-way ANOVA analyses followed by a Holm-Sidak test were calculated in SigmaPlot 13 (*P* > 0.05: ns; *P* ≤ 0.05: *P ≤ 0.01: **P ≤ 0.001: ***).

### Molecular dynamics simulations

We used the TopModel program^61,62^ to build a homology model of ScGrx7. The crystal structure of HsGrx5 (PDB-ID: 2WUL)^37^ was used to prepare the simulations for HsGrx5 WT and HsGrx5^RR^, while for HsGrx5^loop^ and HsGrx5^RR+loop^, models were created with TopModel. The ScGrx7 mutants K105E, K105R, and E147K as well as HsGrx5^RR^ were created by deleting the side-chain atoms of the wild-type residue and rebuilding the respective variant with LEaP. To mimic the reductive half reaction, a glutathione moiety was covalently attached to the active-site cysteine via a disulfide bond in all systems.

From the models of ScGrx7 and HsGrx5, solvated systems were built using PACKMOL Memgen^63^ with 100 mM of GS^–^ molecules in a TIP3P water box^64^; K^+^ ions^65,66^ were added to neutralize the charge of the system. All MD simulations were performed using the the GPU implementation^67^ of the AMBER 18 suite of programs, with the ff14SB force field for the proteins^68^. Since the bond between glutamate and cysteine in glutathione is not a regular peptide bond but a γ-peptide bond for which no corresponding residue exists in the ff14SB force field, we first derived force field parameters for the N-terminal γ-glutamyl residue. To this end, we derived atom-centered point charges for γ-glutamylmethylamide by first performing a gas phase geometry optimization at the HF/6-31G(d) level with GAUSSIAN 09, Revision B.01. To ensure invariance of the molecular electrostatic potential (MEP) with respect to the molecular orientation, the subsequent calculation of the MEP (level of theory: HF/6-31G(d)) and the fitting of the point charges to reproduce the MEP were performed on the R.E.D. server^69^, which uses a rigid-body reorientation algorithm^70^ before calculating the electrostatic potential. The MEP was calculated on four layers defined by scaling the atomic van der Waals radii by factors of 1.4, 1.8, 2.0, and 2.2, respectively, and a point density of 0.28 points au^−2^ (1 pt Å^−2^). Charge fitting was performed using the RESP procedure with two fitting stages (hyperbolic constraint values: 0.0005/0.001), and intramolecular charge constraints on the *N*-methylamide fragment of γ glutamylmethylamide with a target value of zero were employed for charge derivation^71,72^. Lastly, the charge constrained atoms were removed to obtain the γ-glutamyl residue. Force field parameters for the γ-glutamyl residue were fully assigned by the R.E.D. server^73^ using the ff14SB force field. The γ-glutamyl residue was then used in the disulfide glutathione as well as the free GS^−^ molecules in the solution.

Minimization, equilibration, and thermalization were carried out as described previously^73^. In the production simulations, the particle mesh Ewald method^74^ was used to treat long-range electrostatic interactions, and bonds involving hydrogen atoms were constrained using the SHAKE algorithm. A time step of 4 fs was used in accordance with the hydrogen mass repartitioning scheme^71^. The direct-space, non-bonded cutoff was 10 Å. Four independent replications of each system were simulated for 500 ns each in NVT (constant number of particles, constant volume, constant temperature) conditions. The system state was saved every 20 ps. This setup allowed us to observe the unbiased diffusion^72,75^ of GS^−^ around the Grx proteins. Binding frequencies were calculated as the fraction of frames with a binding event of all the frames per simulation replication. Binding events were then clustered with the agglomerative hierarchical clustering algorithm of cpptraj^76^ included in the AMBER 18 program suite. Further geometric analyses, including side chain dihedrals and density grids of GS^–^ diffusion, were also performed with cpptraj^76^.

### Reporting summary

Further information on research design is available in the Nature Research Reporting Summary linked to this article.

## Supplementary information

Supplementary Information

Peer Review

Reporting Summary

## Data Availability

All relevant data are included in the paper or its Supplementary information and are available from the authors upon request. The source data underlying Figs. 3 and 7 as well as Supplementary Fig. 17 are provided as a Source Data file. The MD simulations data underlying Figs. 8 and 9 have been deposited at 10.25838/d5p-10 and 10.25838/d5p-11.

## Source data

Source Data
