# Supplementary material for: Quantitative assessment of the determinant structural differences between redox-active and inactive glutaredoxins
Source: Nat Commun. 2020 Apr 7;11:1725. doi: 10.1038/s41467-020-15441-3 (PMC7138851; doi:10.1038/s41467-020-15441-3)
Supplement: Supplementary file 1 — Supplementary Information [file 41467_2020_15441_MOESM1_ESM.pdf]

Supplementary Information

Quantitative assessment of the determinant structural differences between redox-active and inactive glutaredoxins

Linda Liedgens[1§], Jannik Zimmermann[2§], Lucas Wäschenbach[3§], Fabian Geissel[1], Hugo Laporte[2], Holger Gohlke[3*], Bruce Morgan[2*] & Marcel Deponte[1*]

[1] Fachbereich Chemie, Abteilung Biochemie, Technische Universität Kaiserslautern, D-67663 Kaiserslautern, Germany

[2] Institut für Biochemie, Zentrum für Human- und Molekularbiologie (ZHMB), Universität des Saarlandes, D-66123 Saarbrücken, Germany

[3] Mathematisch-Naturwissenschaftliche Fakultät, Institut für Pharmazeutische und Medizinische Chemie, Heinrich-Heine-Universität Düsseldorf , D-40225 Düsseldorf , Germany.

[4] John von Neumann Institute for Computing (NIC), Jülich Supercomputing Centre (JSC) & Institute of Complex Systems, ICS-6: Structural Biochemistry, Forschungszentrum Jülich GmbH, D-52425 Jülich, Germany

[§]These authors contributed equally to this work.

*Correspondence and requests for materials should be addressed to M.D. (deponte@chemie.uni-kl.de), B.M. (bruce.morgan@uni-saarland.de) and H.G. (gohlke@uni-duesseldorf.de)

Supplementary Figure 1

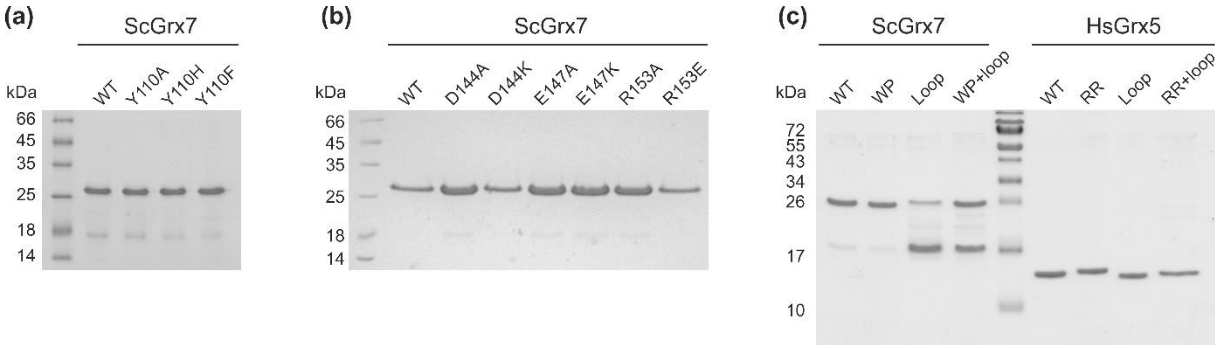

Supplementary Figure 1 | Purification of recombinant wild-type and mutant enzymes. SDS-PAGE analyses of representative eluates on 15% gels after protein purification by Ni-NTA affinity chromatography. (**a**) Tyr110 mutants of ScGrx7. ScGrx7 wild-type enzyme, WT; ScGrx7^{Y110A}, Y110A; ScGrx7^{Y110H}, Y110H; ScGrx7^{Y110F}, Y110F. (**b**) Asp144, Glu147 and Arg153 mutants of ScGrx7. ScGrx7 wild-type enzyme, WT; ScGrx7^{D144A}, D144A; ScGrx7^{D144K}, D144K, ScGrx7^{E147A}, E147A; ScGrx7^{E147K}, E147K; ScGrx7^{R153A}, R153A; ScGrx7^{R153E}, R153E. Average yields from up to seven independent protein purification experiments for each mutant were highly reproducible and ranged from 12.6 ± 4.1 to 53.7 ± 5.1 mg of recombinant ScGrx7 per liter of *E. coli* culture depending on the mutant. (**c**) Interconversion mutants of ScGrx7 and HsGrx5. Left side: ScGrx7 wild-type enzyme, WT; ScGrx7^{WP}, WP; ScGrx7^{loop}, Loop; ScGrx7$^{WP+loop}$, WP+loop. Right side: Wild-type HsGrx5, WT; HsGrx5^{RR}, RR; HsGrx5^{loop}, Loop; HsGrx5$^{RR+loop}$, RR+loop. The calculated molecular masses of recombinant wild-type ScGrx7 and HsGrx5 are 20.2 and 15.1 kDa, respectively. ScGrx7 runs at approximately 26 kDa as reported previously. The exchange of the active site loop in ScGrx7 results in partial proteolysis. Average yields from three independent protein purification experiments for each mutant were highly reproducible and ranged from 2.2 to 16.2 mg of recombinant protein per liter of *E. coli* culture depending on the mutant.

Supplementary Figure 2

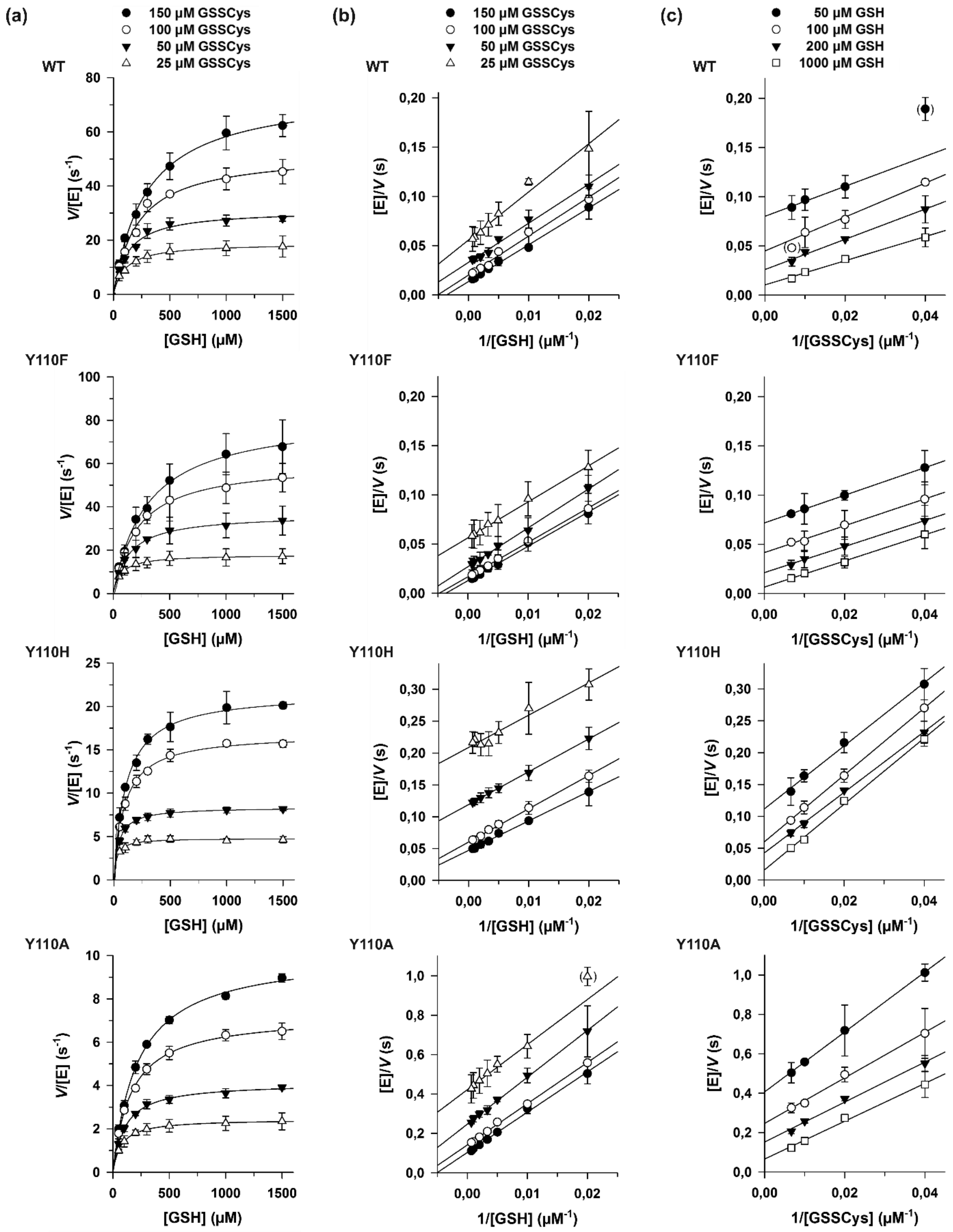

Supplementary Figure 2 | GSSCys assay steady-state kinetics of ScGrx7 wild-type enzyme and Y110X mutants. (**a**) Michaelis-Menten plots of the GSH-dependent reaction velocity at different initial concentrations of GSSCys. (**b**) Lineweaver-Burk plots of the GSH-

dependent reaction velocity at different initial concentrations of GSSCys revealing ping-pong kinetic patterns. (**c**) Lineweaver-Burk plots of the GSSCys-dependent reaction velocity at different initial concentrations of GSH. Data points and error bars are the mean ± s.d. from three independent protein purification experiments and were plotted and fitted in SigmaPlot 13 according to Michaelis-Menten, Lineweaver-Burk, Eadie-Hofstee and Hanes theory (the latter two plots are not shown). Calculated k_{cat}^{app} and K_m^{app} values from the four different plots usually varied by less than 10%. Data points in brackets were omitted from the regression analysis when the k_{cat}^{app} or K_m^{app} values from all four plots varied by more than 10% and converged after removal of the outlier. True kinetic constants were estimated from secondary plots in Supplementary Fig. 3 and are listed in Supplementary Table 1. Selected apparent kinetic constants from non-linear regression analyses of Michaelis-Menten plots are listed in Supplementary Table 2.

Supplementary Figure 3

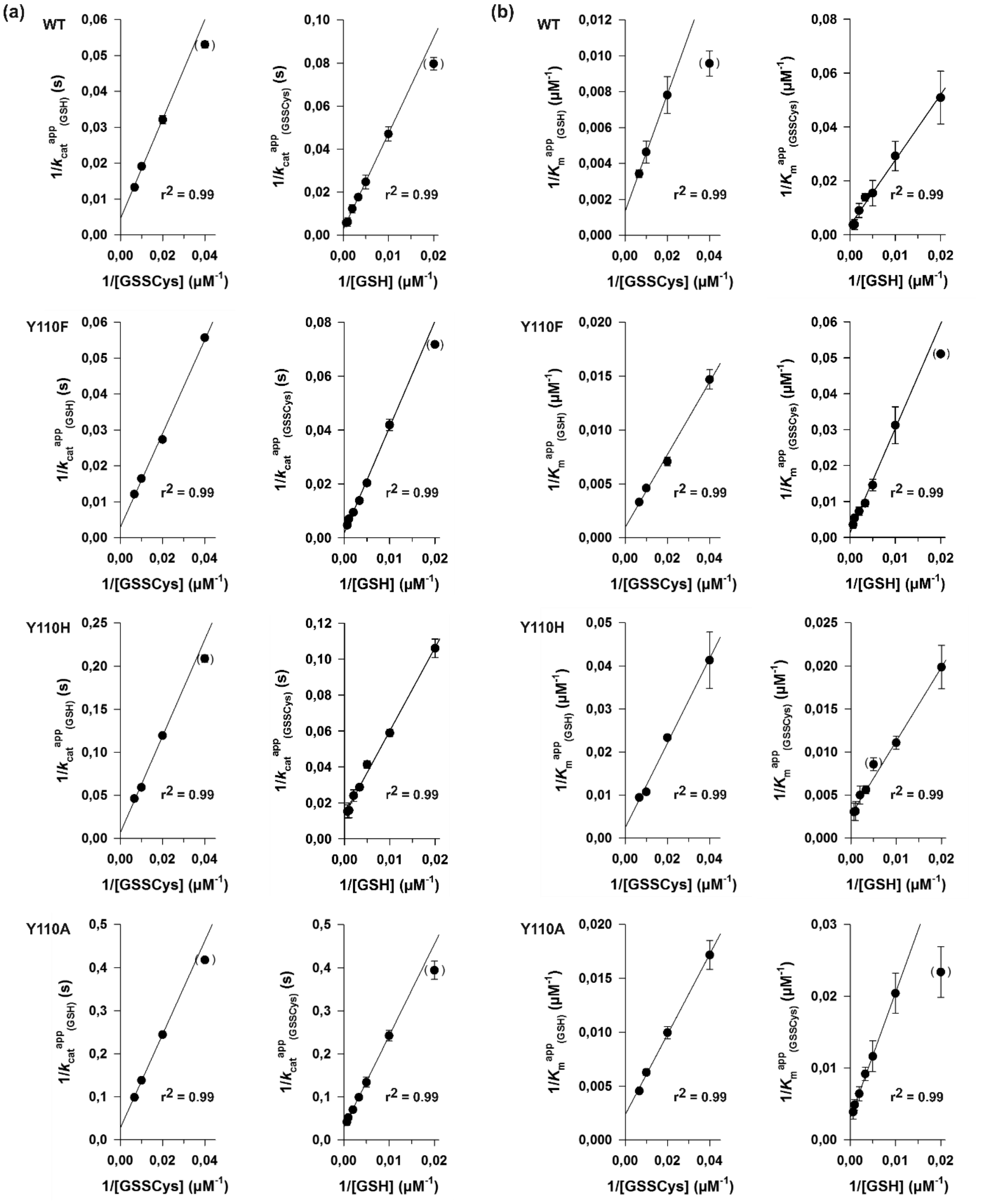

Supplementary Figure 3 | GSSCys assay secondary plots for ScGrx7 wild-type enzyme and Y110X mutants. (**a**) Secondary plots of the k_{cat}^{app} values at different concentrations of GSSCys (left panels) and GSH (right panels) allowing the estimation of the true k_{cat} value from the y-axis intercept ($1/k_{cat}$) and of the true K_m value from the x-axis intercept ($-1/K_m$). Estimated

true kinetic constants have to be interpreted with care because of the proximity of the intersection points to the origin of the graphs. Dalziel coefficients were obtained from the slopes. (**b**) Secondary plots of the K_m^{app} values at different concentrations of GSSCys (left panels) and GSH (right panels). K_m^{app} and k_{cat}^{app} values were obtained from non-linear regression analyses of Michaelis-Menten plots (Supplementary Fig. 2). Error bars are the calculated standard error from the hyperbolic curve fits in SigmaPlot 13. Outliers in brackets were identified based on the r^2 values and omitted from the linear regression analysis in SigmaPlot 13. Estimated true kinetic constants and Dalziel coefficients are listed in Supplementary Table 1.

Supplementary Figure 4

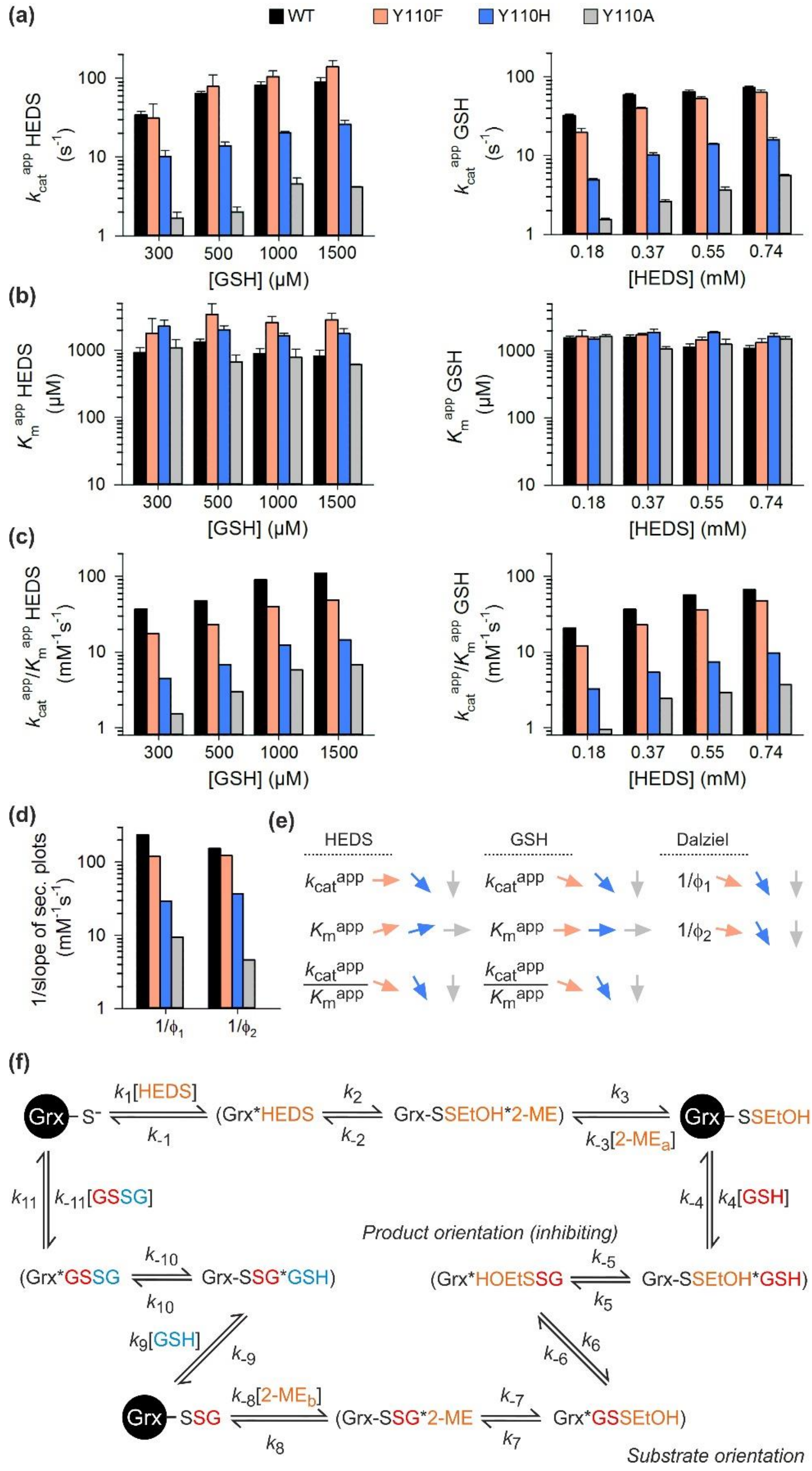

Supplementary Figure 4 | Replacement of Tyr110 in ScGrx7 slows down the turnover of both substrates in the HEDS assay. (**a**) and (**b**) Selected k_{cat}^{app} and K_{m}^{app} values of ScGrx7 wild- type enzyme and Y110X mutants for HEDS and GSH. (**c**) Calculated catalytic efficiencies

from panels **a** and **b**. Original plots and kinetic parameters for panels **a**-**c** are shown in Supplementary Fig. 5 and Supplementary Table 3. Error bars are the calculated standard error from the curve fits in SigmaPlot 13. (**d**) Reciprocal Dalziel coefficients obtained from Supplementary Fig. 6. The reciprocal Dalziel coefficients and true k_{cat} values are also listed in Supplementary Table 1. (**e**) Summary of the altered kinetic parameters. (**f**) Potential reaction sequence for the HEDS assay. Please note that GSSEtOH is a product and substrate that can bind in two orientations at the Grx active site. This could explain the sequential kinetic patterns with a common x-axis intercept in the Lineweaver-Burk plots (Supplementary Fig. 5) as discussed previously[12]. Statistical analyses and *P*-values for the k_{cat}^{app} and K_m^{app} values from panels **a** and **b** are listed in Supplementary Table 11.

Supplementary Figure 5

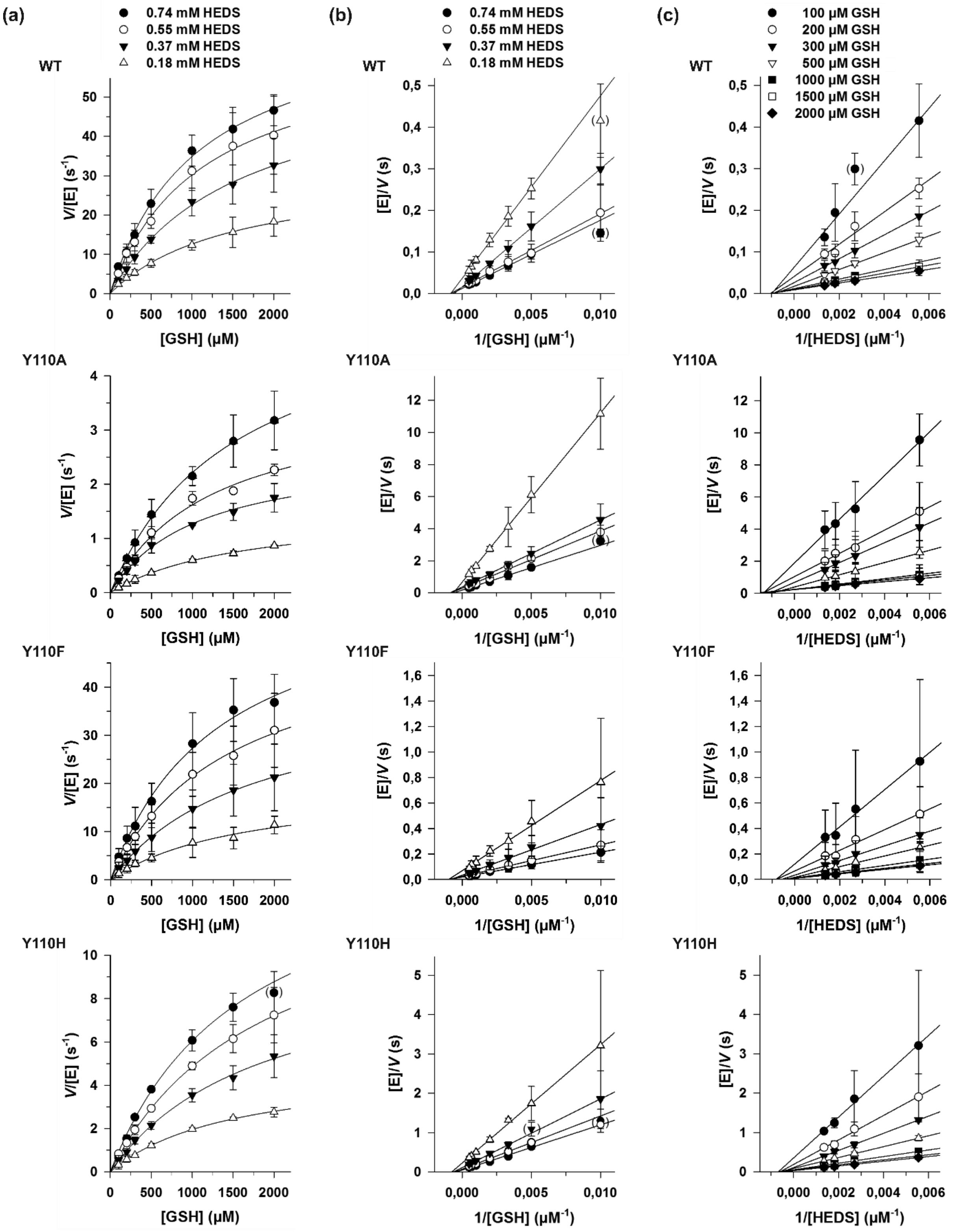

Supplementary Figure 5 | HEDS assay steady-state kinetics of ScGrx7 wild-type enzyme and Y110X mutants. (**a**) Michaelis-Menten plots of the GSH-dependent reaction velocity at different initial concentrations of HEDS. (**b**) Lineweaver-Burk plots of the GSH-dependent

reaction velocity at different initial concentrations of HEDS revealing sequential kinetic patterns. (**c**) Lineweaver-Burk plots of the HEDS-dependent reaction velocity at different initial concentrations of GSH. Data points and error bars are the mean ± s.d. from three independent protein purification experiments and were plotted and fitted in SigmaPlot 13 according to Michaelis-Menten, Lineweaver-Burk, Eadie-Hofstee and Hanes theory (the latter two plots are not shown). Calculated k_{cat}^{app} and K_m^{app} values from the four different plots usually varied by less than 10%. Data points in brackets were omitted from the regression analysis when the k_{cat}^{app} or K_m^{app} values from all four plots varied by more than 10% and converged after removal of the outlier. True kinetic constants were estimated from secondary plots in Supplementary Fig. 6 and are listed in Supplementary Table 1. Selected apparent kinetic constants from non-linear regression analyses of Michaelis-Menten plots are listed in Supplementary Table 3.

Supplementary Figure 6

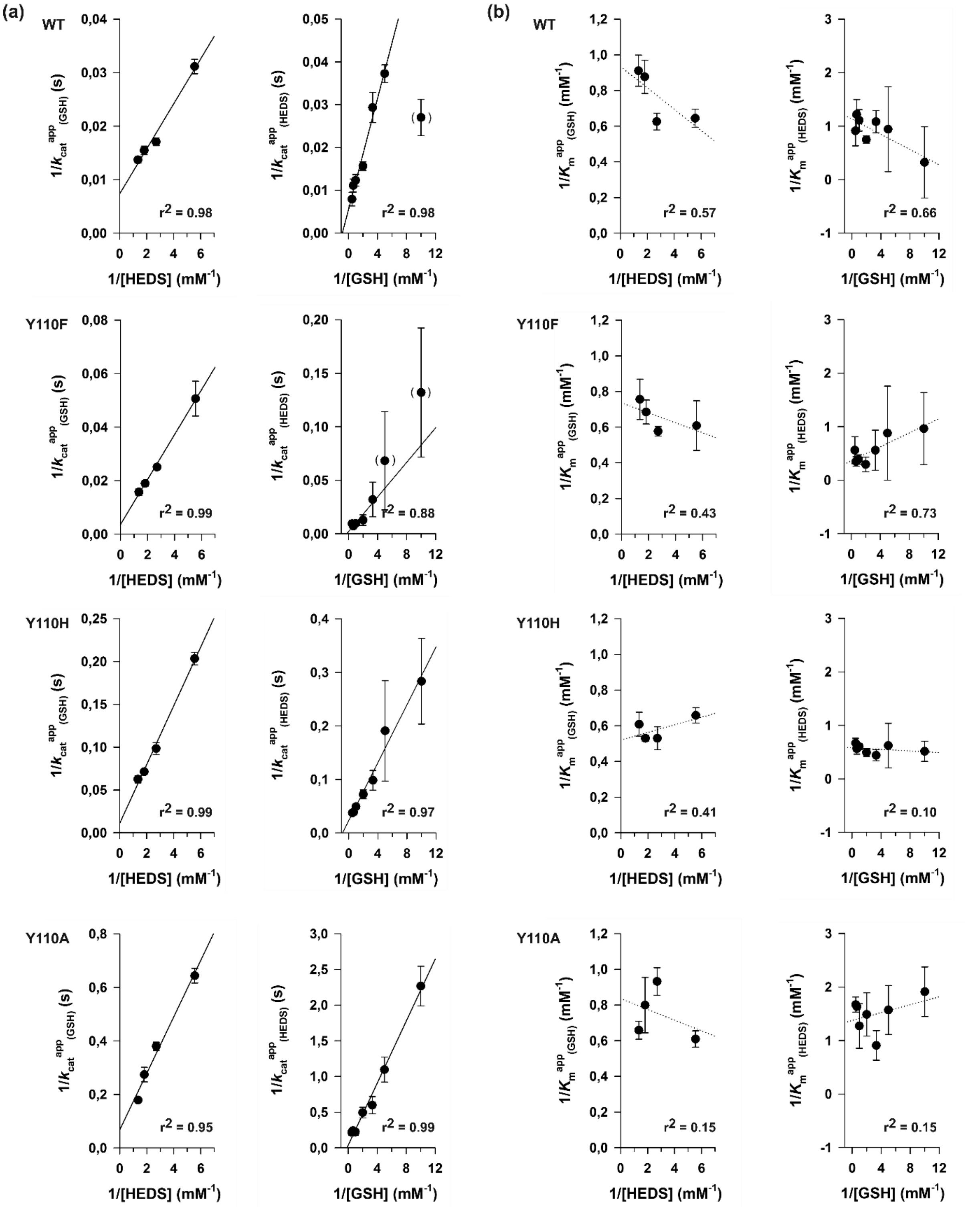

Supplementary Figure 6 | HEDS assay secondary plots for ScGrx7 wild-type enzyme and Y110X mutants. K_m^{app} and k_{cat}^{app} values were obtained from non-linear regression analyses of Michaelis-Menten plots (Supplementary Fig. 5). Error bars are the calculated standard error from the hyperbolic curve fits in SigmaPlot 13. (**a**) Secondary plots of the k_{cat}^{app}

values at different concentrations of HEDS (left panels) and GSH (right panels) allowing the estimation of the true k_{cat} value from the y-axis intercept ($1/k_{cat}$) and of the true K_m value from the x-axis intercept ($-1/K_m$). Outliers in brackets at the lowest substrate concentration were identified based on the r^2 values and omitted from the linear regression analysis in SigmaPlot 13. Estimated true kinetic constants have to be interpreted with care because of the proximity of the intersection points to the origin of the graphs. The Dalziel coefficients were obtained from the slopes. (**b**) Secondary plots of the K_m^{app} values at different concentrations of HEDS (left panels) and GSH (right panels). Please note that the differences for K_m^{app} values among independent measurements and mutants are mostly not statistically significant (see also Supplementary Table 3). Estimated true kinetic constants and Dalziel coefficients are listed in Supplementary Table 1.

Supplementary Figure 7

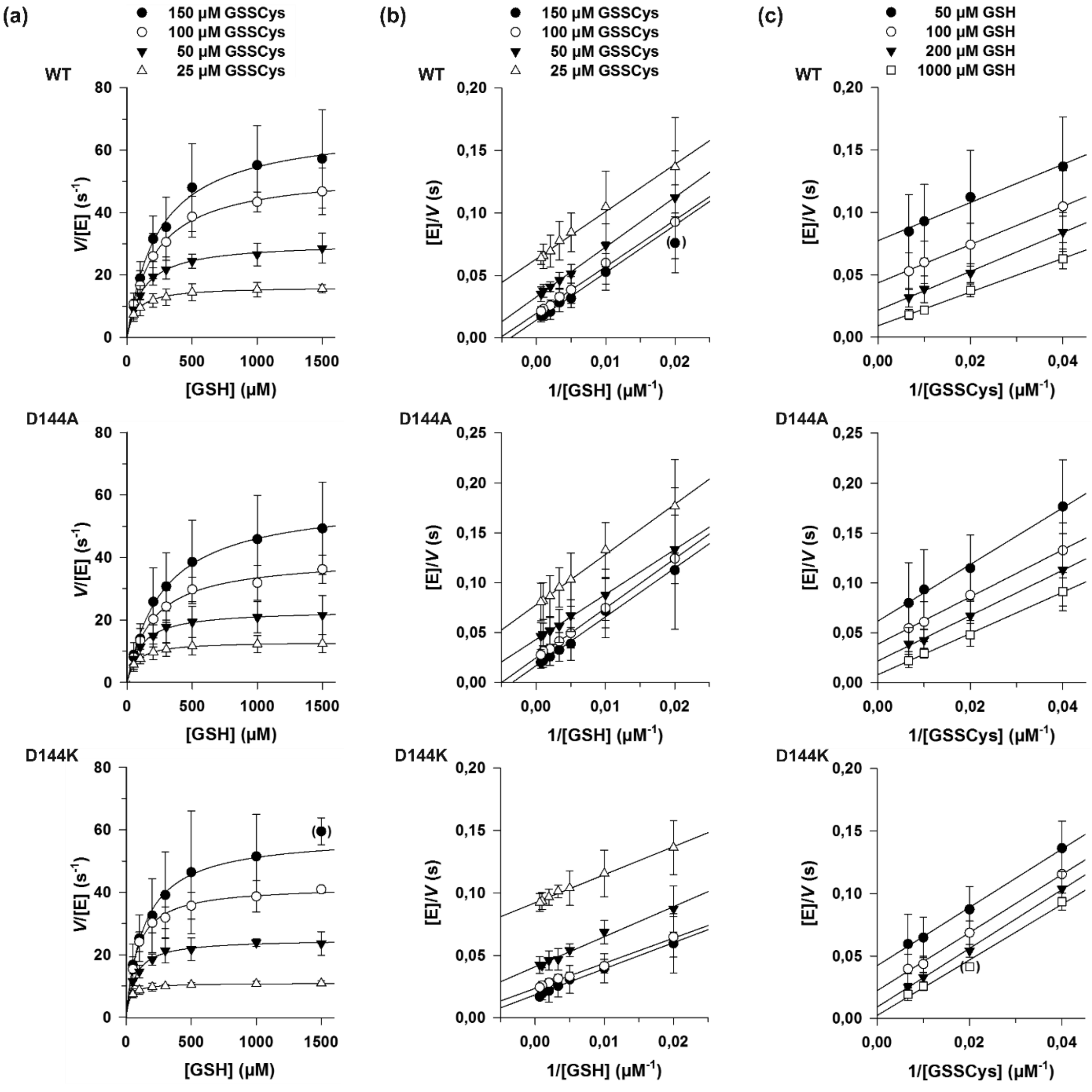

Supplementary Figure 7 | GSSCys assay steady-state kinetics of ScGrx7 wild-type enzyme and D144X mutants. (**a**) Michaelis-Menten plots of the GSH-dependent reaction velocity at different initial concentrations of GSSCys. (**b**) Lineweaver-Burk plots of the GSH-dependent reaction velocity at different initial concentrations of GSSCys revealing ping-pong kinetic patterns. (**c**) Lineweaver-Burk plots of the GSSCys-dependent reaction velocity at different initial concentrations of GSH. Data points and error bars are the mean ± s.d. from three independent protein purification experiments and were plotted and fitted in SigmaPlot 13 according to Michaelis-Menten, Lineweaver-Burk, Eadie-Hofstee and Hanes theory (the latter

two plots are not shown). Calculated k_{cat}^{app} and K_m^{app} values from the four different plots usually varied by less than 10%. Data points in brackets were omitted from the regression analysis when the k_{cat}^{app} or K_m^{app} values from all four plots varied by more than 10% and converged after removal of the outlier. True kinetic constants were estimated from secondary plots in Supplementary Fig. 8 and listed in Supplementary Table 4. Selected apparent kinetic constants from non-linear regression analyses of Michaelis-Menten plots are listed in Supplementary Table 5.

Supplementary Figure 8

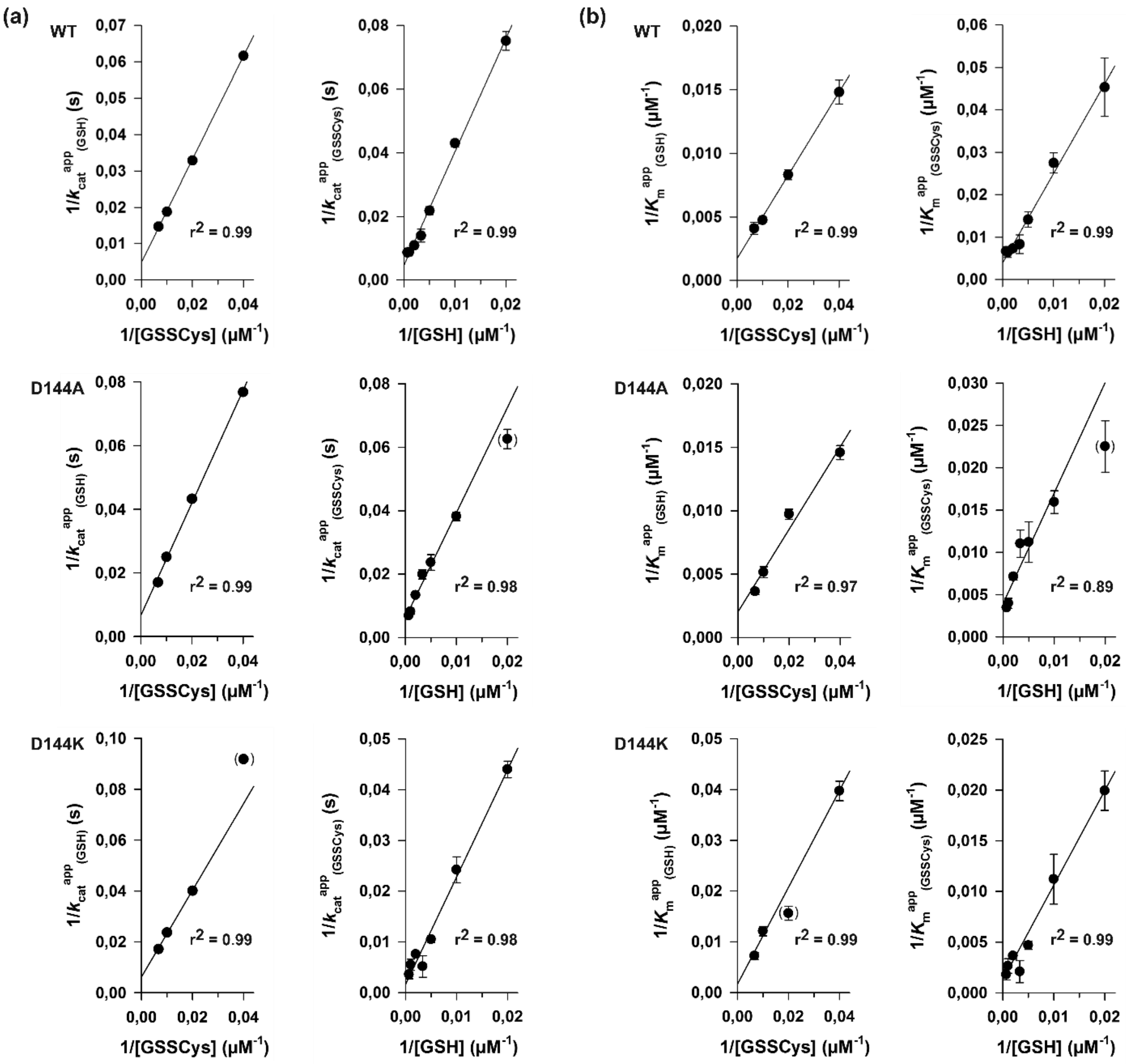

Supplementary Figure 8 | GSSCys assay secondary plots for ScGrx7 wild-type enzyme and D144X mutants. (**a**) Secondary plots of the k_{cat}^{app} values at different concentrations of GSSCys (left panels) and GSH (right panels) allowing the estimation of the true k_{cat} value from the y-axis intercept ($1/k_{cat}$) and of the true K_m value from the x-axis intercept ($-1/K_m$). Estimated true kinetic constants have to be interpreted with care because of the proximity of the intersection points to the origin of the graphs. Dalziel coefficients were obtained from the slopes. (**b**) Secondary plots of the K_m^{app} values at different concentrations of GSSCys (left panels) and GSH (right panels). K_m^{app} and k_{cat}^{app} values were obtained from non-linear regression analyses of Michaelis-Menten plots (Supplementary Fig. 7). Error bars are the

calculated standard error from the hyperbolic curve fits in SigmaPlot 13. Outliers in brackets were identified based on the r^2 values and omitted from the linear regression analysis in SigmaPlot 13. Estimated true kinetic constants and Dalziel coefficients are listed in Supplementary Table 4.

Supplementary Figure 9

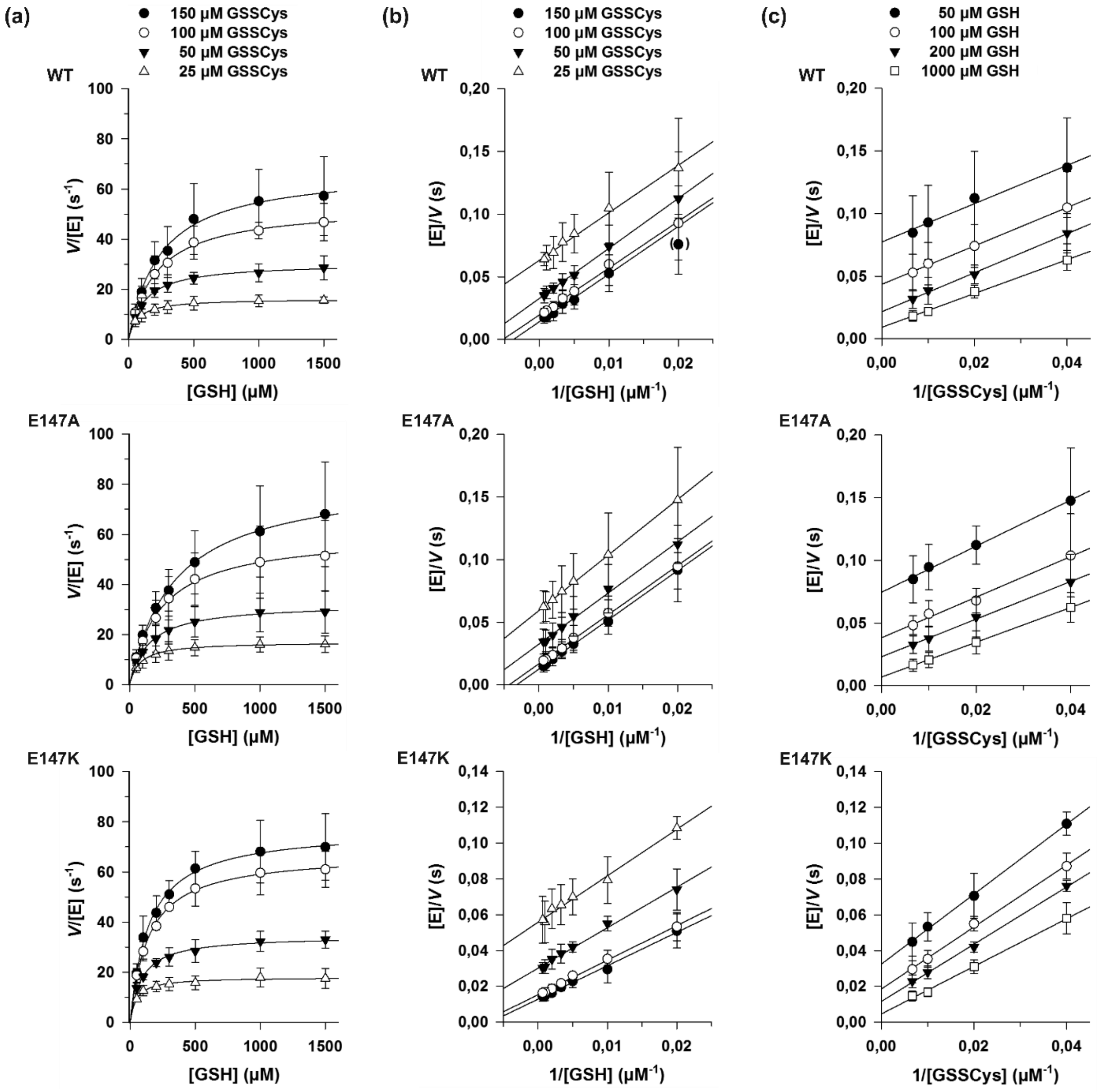

Supplementary Figure 9 | GSSCys assay steady-state kinetics of ScGrx7 wild-type enzyme and E147X mutants. (**a**) Michaelis-Menten plots of the GSH-dependent reaction velocity at different initial concentrations of GSSCys. (**b**) Lineweaver-Burk plots of the GSH-dependent reaction velocity at different initial concentrations of GSSCys revealing ping-pong kinetic patterns. (**c**) Lineweaver-Burk plots of the GSSCys-dependent reaction velocity at different initial concentrations of GSH. Data points and error bars are the mean ± s.d. from three independent protein purification experiments and were plotted and fitted in SigmaPlot 13 according to Michaelis-Menten, Lineweaver-Burk, Eadie-Hofstee and Hanes theory (the latter

two plots are not shown). Calculated k_{cat}^{app} and K_m^{app} values from the four different plots usually varied by less than 10%. Data points in brackets were omitted from the regression analysis when the k_{cat}^{app} or K_m^{app} values from all four plots varied by more than 10% and converged after removal of the outlier. True kinetic constants were estimated from secondary plots in Supplementary Fig. 10 and are listed in Supplementary Table 4. Selected apparent kinetic constants from non-linear regression analyses of Michaelis-Menten plots are listed in Supplementary Table 5.

Supplementary Figure 10

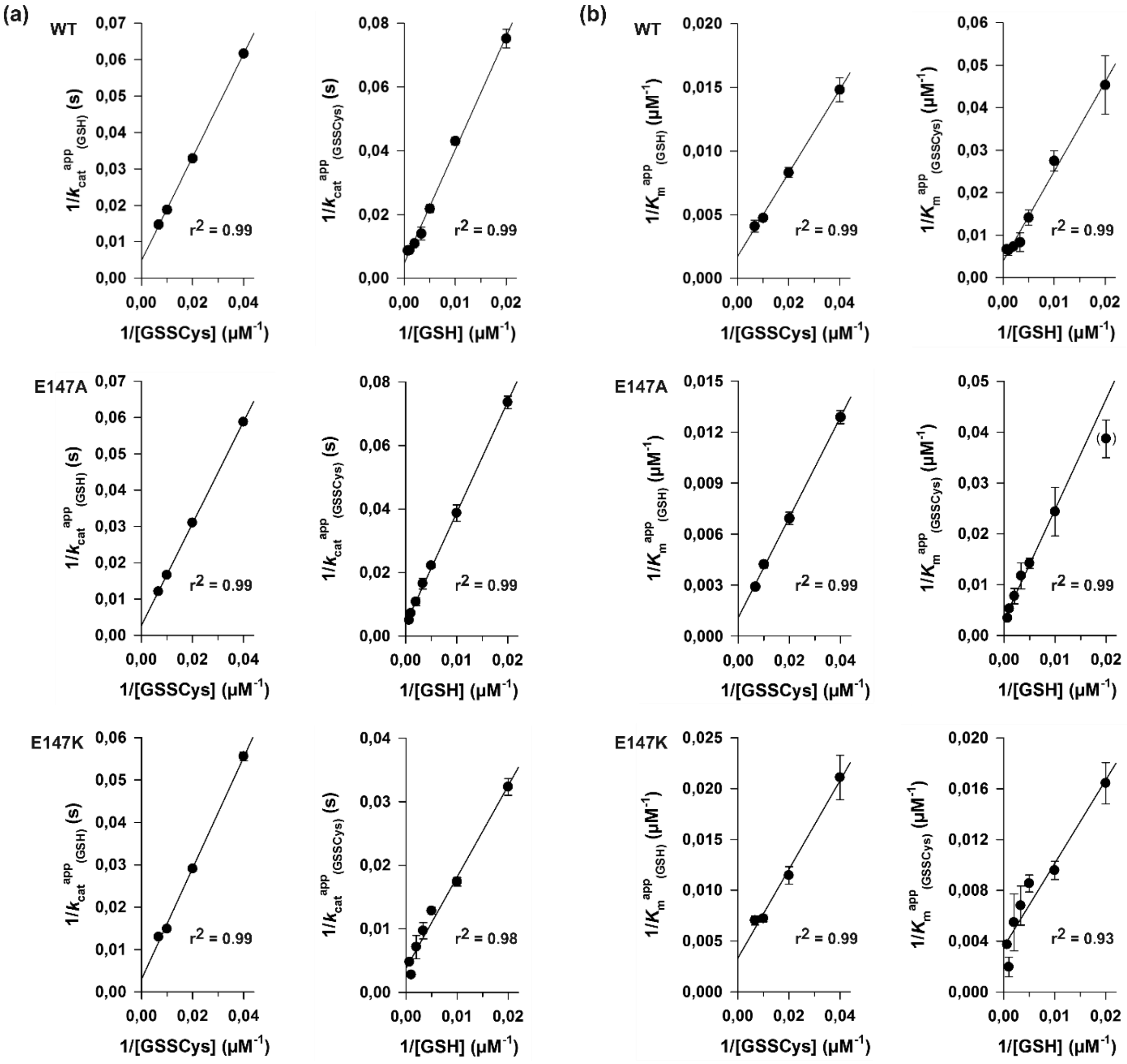

Supplementary Figure 10 | GSSCys assay secondary plots for ScGrx7 wild-type enzyme and E147X mutants. (**a**) Secondary plots of the k_{cat}^{app} values at different concentrations of GSSCys (left panels) and GSH (right panels) allowing the estimation of the true k_{cat} value from the y-axis intercept ($1/k_{cat}$) and of the true K_m value from the x-axis intercept ($-1/K_m$). Estimated true kinetic constants have to be interpreted with care because of the proximity of the intersection points to the origin of the graphs. Dalziel coefficients were obtained from the slopes. (**b**) Secondary plots of the K_m^{app} values at different concentrations of GSSCys (left panels) and GSH (right panels). K_m^{app} and k_{cat}^{app} values were obtained from non-linear regression analyses of Michaelis-Menten plots (Supplementary Fig. 9). Error bars are the

calculated standard error from the hyperbolic curve fits in SigmaPlot 13. Outliers in brackets were identified based on the r^2 values and omitted from the linear regression analysis in SigmaPlot 13. Estimated true kinetic constants and Dalziel coefficients are listed in Supplementary Table 4.

Supplementary Figure 11

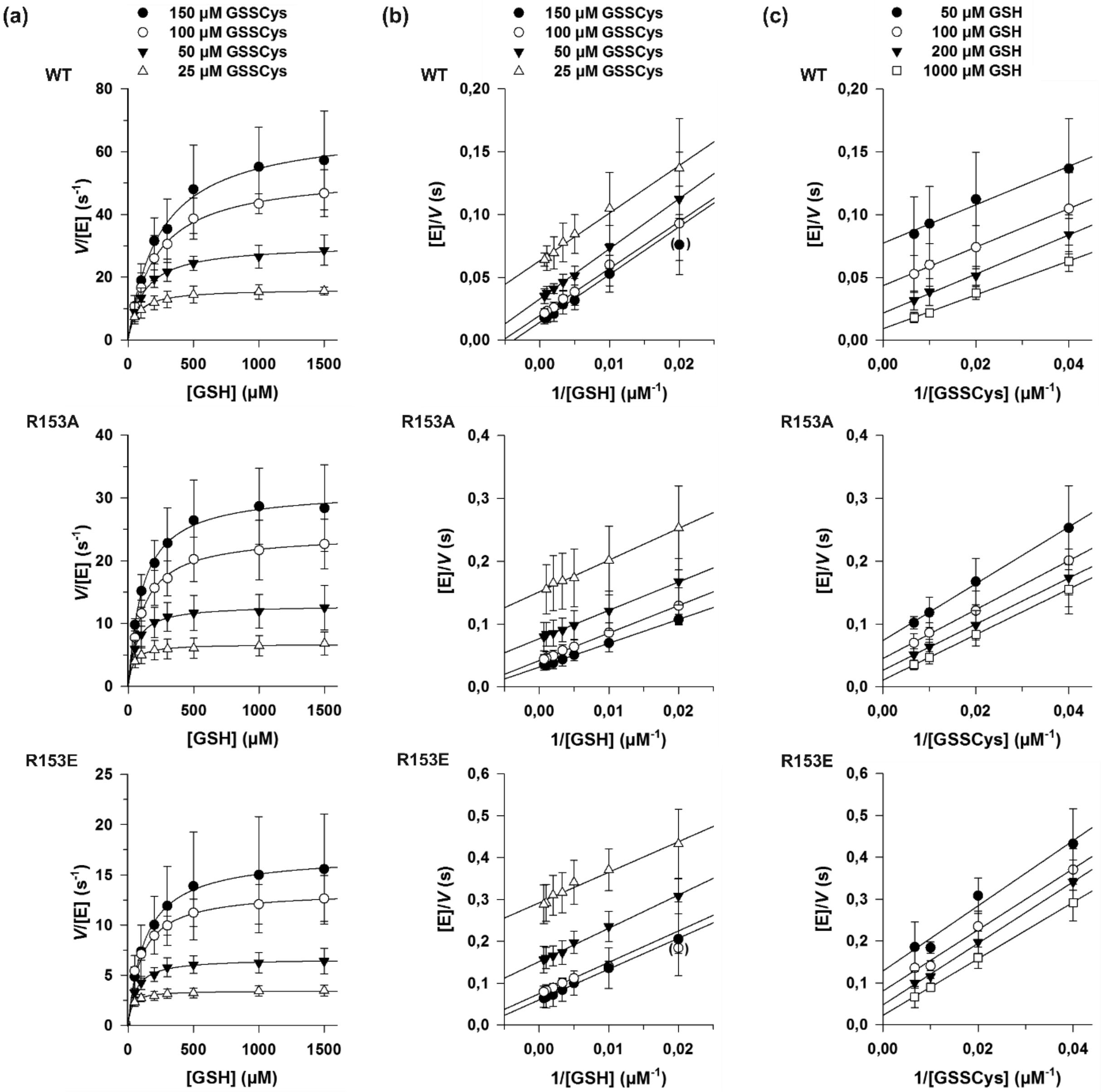

Supplementary Figure 11 | GSSCys assay steady-state kinetics of ScGrx7 wild-type enzyme and R153X mutants. (**a**) Michaelis-Menten plots of the GSH-dependent reaction velocity at different initial concentrations of GSSCys. (**b**) Lineweaver-Burk plots of the GSH-dependent reaction velocity at different initial concentrations of GSSCys revealing ping-pong kinetic patterns. (**c**) Lineweaver-Burk plots of the GSSCys-dependent reaction velocity at different initial concentrations of GSH. Data points and error bars are the mean ± s.d. from three independent protein purification experiments and were plotted and fitted in SigmaPlot 13 according to Michaelis-Menten, Lineweaver-Burk, Eadie-Hofstee and Hanes theory (the latter two plots are not shown). Calculated k_{cat}^{app} and K_m^{app} values from the four different plots usually

varied by less than 10%. Data points in brackets were omitted from the regression analysis when the k_{cat}^{app} or K_m^{app} values from all four plots varied by more than 10% and converged after removal of the outlier. True kinetic constants were estimated from secondary plots in Supplementary Fig. 12 are listed in Supplementary Table 4. Selected apparent kinetic constants from non-linear regression analyses of Michaelis-Menten plots are listed in Supplementary Table 6.

Supplementary Figure 12

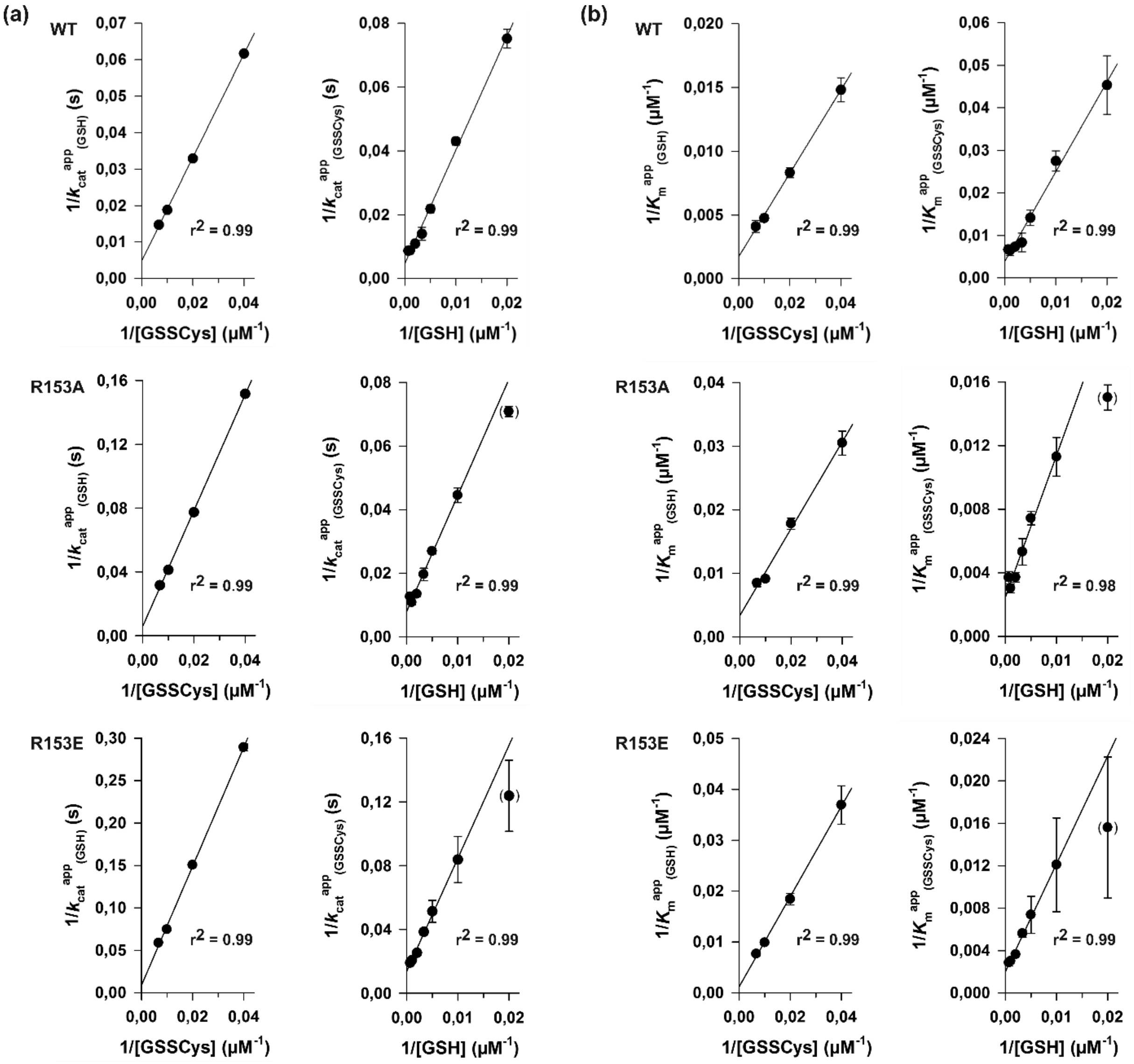

Supplementary Figure 12 | GSSCys assay secondary plots for ScGrx7 wild-type enzyme and R153X mutants. (**a**) Secondary plots of the k_{cat}^{app} values at different concentrations of GSSCys (left panels) and GSH (right panels) allowing the estimation of the true k_{cat} value from the y-axis intercept ($1/k_{cat}$) and of the true K_m value from the x-axis intercept ($-1/K_m$). Estimated true kinetic constants have to be interpreted with care because of the proximity of the intersection points to the origin of the graphs. Dalziel coefficients were obtained from the slopes. (**b**) Secondary plots of the K_m^{app} values at different concentrations of GSSCys (left panels) and GSH (right panels). K_m^{app} and k_{cat}^{app} values were obtained from non-linear regression analyses of Michaelis-Menten plots (Supplementary Fig. 11). Error bars are the

calculated standard error from the hyperbolic curve fits in SigmaPlot 13. Outliers in brackets were identified based on the r^2 values and omitted from the linear regression analysis in SigmaPlot 13. Estimated true kinetic constants and Dalziel coefficients are listed in Supplementary Table 4.

Supplementary Figure 13

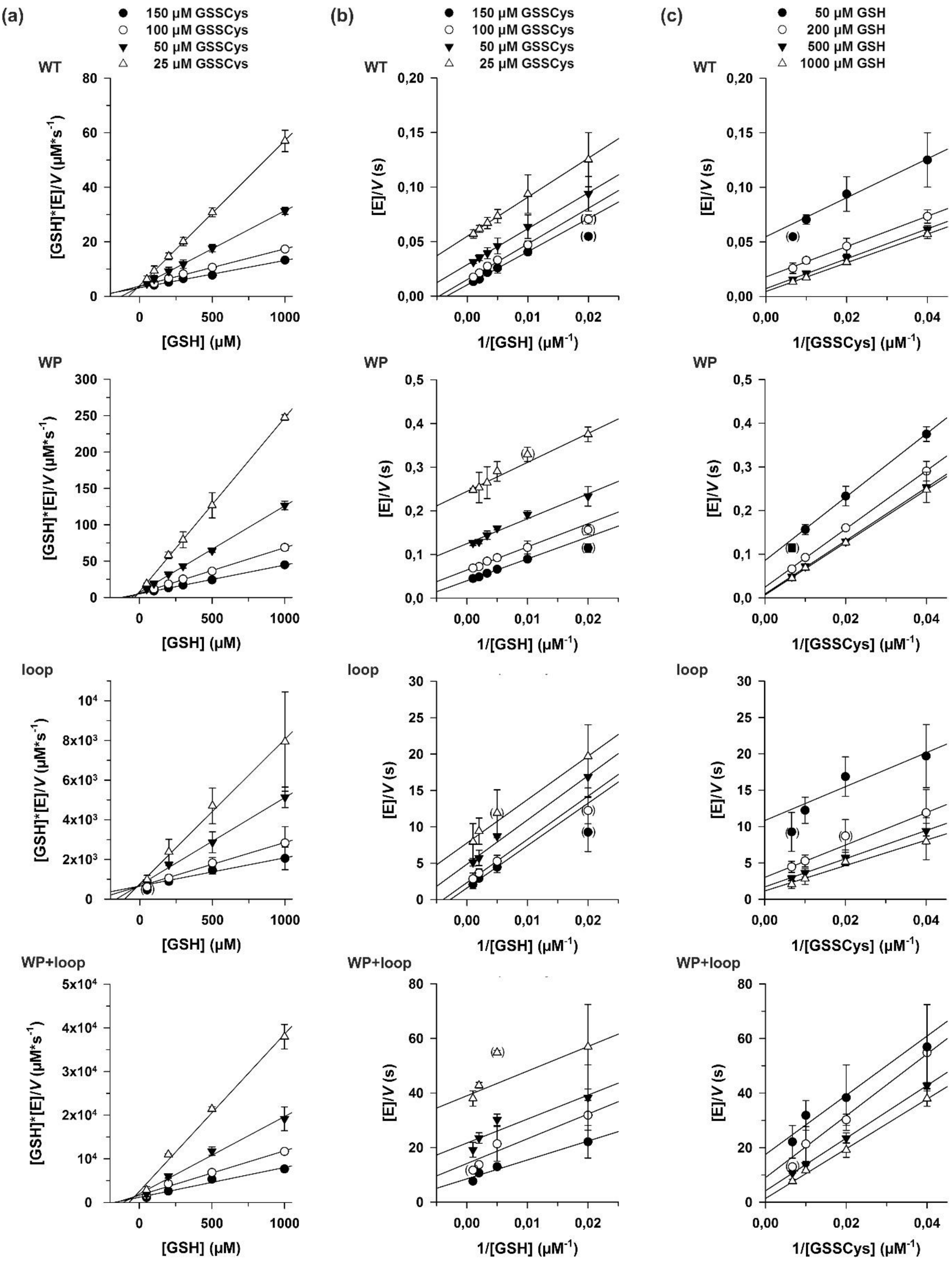

Supplementary Figure 13 | GSSCys assay steady-state kinetics of ScGrx7 wild-type enzyme and mutants ScGrx7^{WP}, ScGrx7^{loop} and ScGrx7$^{WP+loop}$. (**a**) Hanes plots of the GSH-dependent reaction velocity at different initial concentrations of GSSCys. (**b**) Lineweaver-Burk

plots of the GSH-dependent reaction velocity at different initial concentrations of GSSCys revealing ping-pong kinetic patterns. (**c**) Lineweaver-Burk plots of the GSSCys-dependent reaction velocity at different initial concentrations of GSH. Data points and error bars are the mean ± s.d. from three independent protein purification experiments and were plotted and fitted in SigmaPlot 13 according to Hanes, Lineweaver-Burk and Eadie-Hofstee theory (the latter plots are not shown). Calculated k_{cat}^{app} and K_m^{app} values from the three different plots usually varied by less than 10%. Data points in brackets were omitted from the regression analysis when the k_{cat}^{app} or K_m^{app} values from all four plots varied by more than 10% and converged after removal of the outlier. True kinetic constants were estimated from secondary plots in Supplementary Fig. 14 and are listed in Supplementary Table 7. Selected apparent kinetic constants from linear regression analyses of Hanes plots are listed in Supplementary Table 8.

Supplementary Figure 14

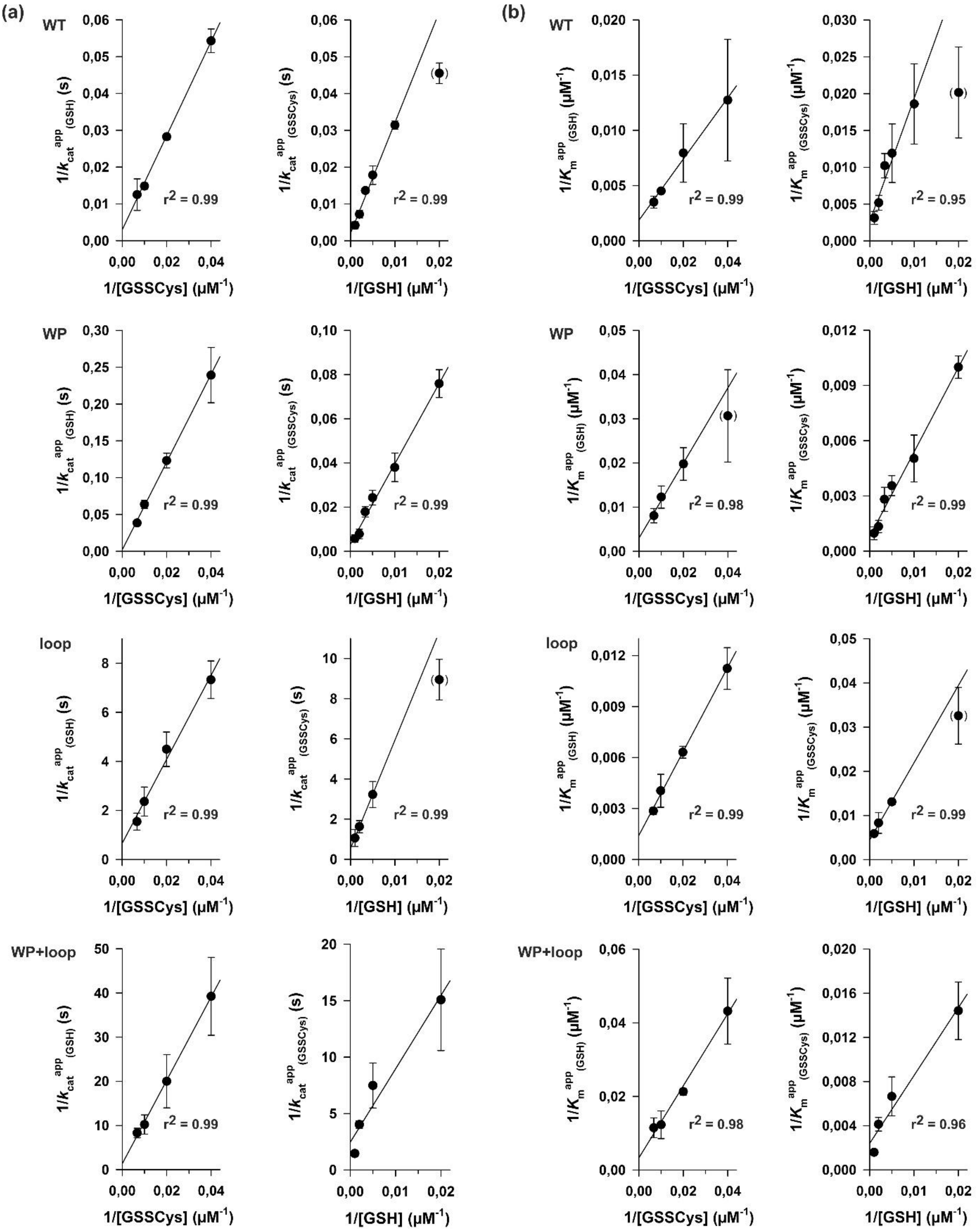

Supplementary Figure 14 | GSSCys assay secondary plots for ScGrx7 wild-type enzyme and mutants ScGrx7^{WP}, ScGrx7^{loop} and ScGrx7$^{WP+loop}$. (**a**) Secondary plots of the k_{cat}^{app} values at different concentrations of GSSCys (left panels) and GSH (right panels) allowing the

estimation of the true k_{cat} value from the y-axis intercept ($1/k_{cat}$) and of the true K_m value from the x-axis intercept ($-1/K_m$). Estimated true kinetic constants have to be interpreted with care because of the proximity of the intersection points to the origin of the graphs. Dalziel coefficients were obtained from the slopes. (**b**) Secondary plots of the K_m^{app} values at different concentrations of GSSCys (left panels) and GSH (right panels). K_m^{app} and k_{cat}^{app} values were obtained from linear regression analyses of Hanes plots (Supplementary Fig. 13). Error bars are the calculated standard error from the hyperbolic curve fits in SigmaPlot 13. Outliers in brackets were identified based on the r^2 values and omitted from the linear regression analysis in SigmaPlot 13. Estimated true kinetic constants and Dalziel coefficients are listed in Supplementary Table 7.

Supplementary Figure 15

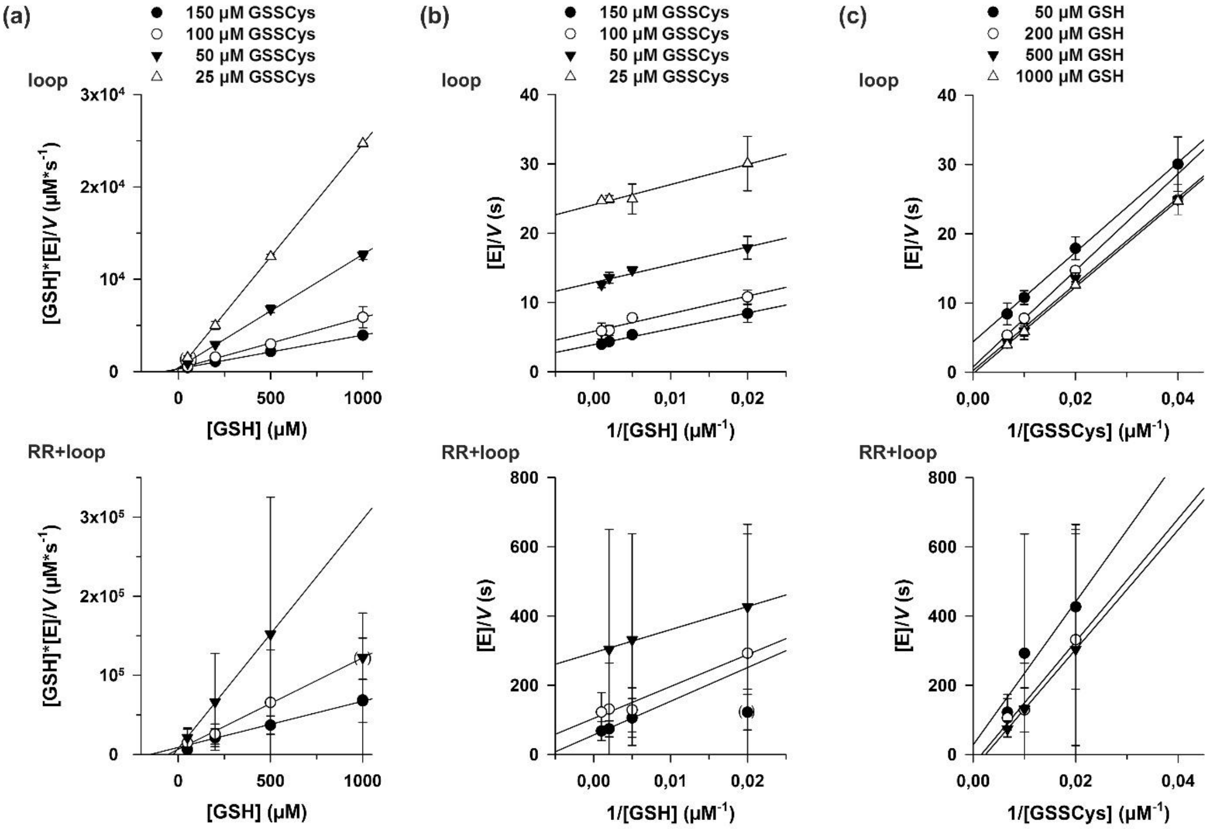

Supplementary Figure 15 | GSSCys assay steady-state kinetics of HsGrx5^{loop} and HsGrx5$^{RR+loop}$. (**a**) Hanes plots of the GSH-dependent reaction velocity at different initial concentrations of GSSCys. (**b**) Lineweaver-Burk plots of the GSH-dependent reaction velocity at different initial concentrations of GSSCys revealing ping-pong kinetic patterns. (**c**) Lineweaver-Burk plots of the GSSCys-dependent reaction velocity at different initial concentrations of GSH. Data points and error bars are the mean ± s.d. from three independent protein purification experiments and were plotted and fitted in SigmaPlot 13 according to Hanes, Lineweaver-Burk and Eadie-Hofstee theory. Data points in brackets were omitted from the regression analysis when the k_{cat}^{app} or K_m^{app} values from all four plots varied by more than 10% and converged after removal of the outlier. True kinetic constants were estimated from secondary plots in Supplementary Fig. 16 and are listed in Supplementary Table 7. Selected apparent kinetic constants from linear regression analyses of Hanes plots are listed in Supplementary Table 9. The activities of HsGrx5 and HsGrx5^{RR} were similar to the NADPH consumption of negative controls without enzyme and could not be analyzed.

Supplementary Figure 16

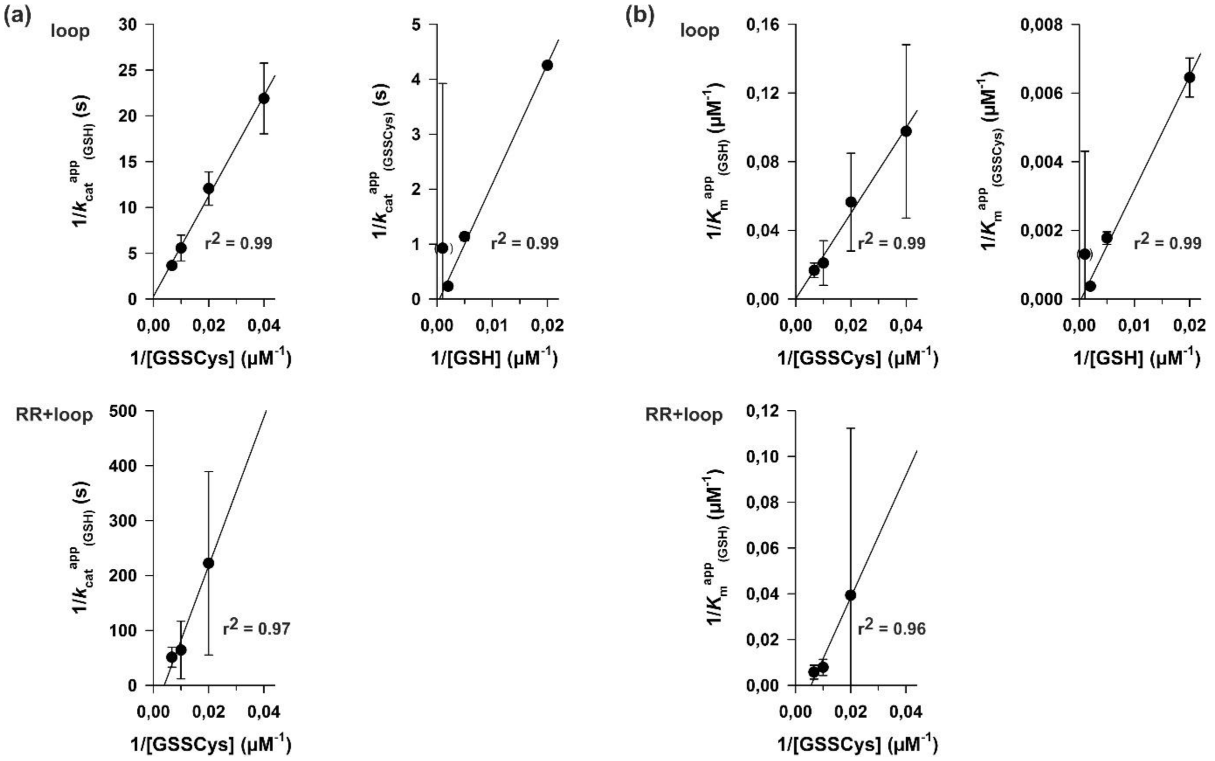

Supplementary Figure 16 | GSSCys assay secondary plots for HsGrx5^{loop} and HsGrx5$^{RR+loop}$. (**a**) Secondary plots of the k_{cat}^{app} values at different concentrations of GSSCys (left panels) and GSH (right panels) allowing the estimation of the true k_{cat} value from the y-axis intercept ($1/k_{cat}$) and of the true K_m value from the x-axis intercept ($-1/K_m$). Estimated true kinetic constants have to be interpreted with care because of the proximity of the intersection points to the origin of the graphs. Dalziel coefficients were obtained from the slopes. (**b**) Secondary plots of the K_m^{app} values at different concentrations of GSSCys (left panels) and GSH (right panels). K_m^{app} and k_{cat}^{app} values were obtained from linear regression analyses of Hanes plots (Supplementary Fig. 15). Error bars are the calculated standard error from the hyperbolic curve fits in SigmaPlot 13. Outliers in brackets were identified based on the r^2 values and omitted from the linear regression analysis in SigmaPlot 13. Estimated true kinetic constants and Dalziel coefficients are listed in Supplementary Table 7.

Supplementary Figure 17

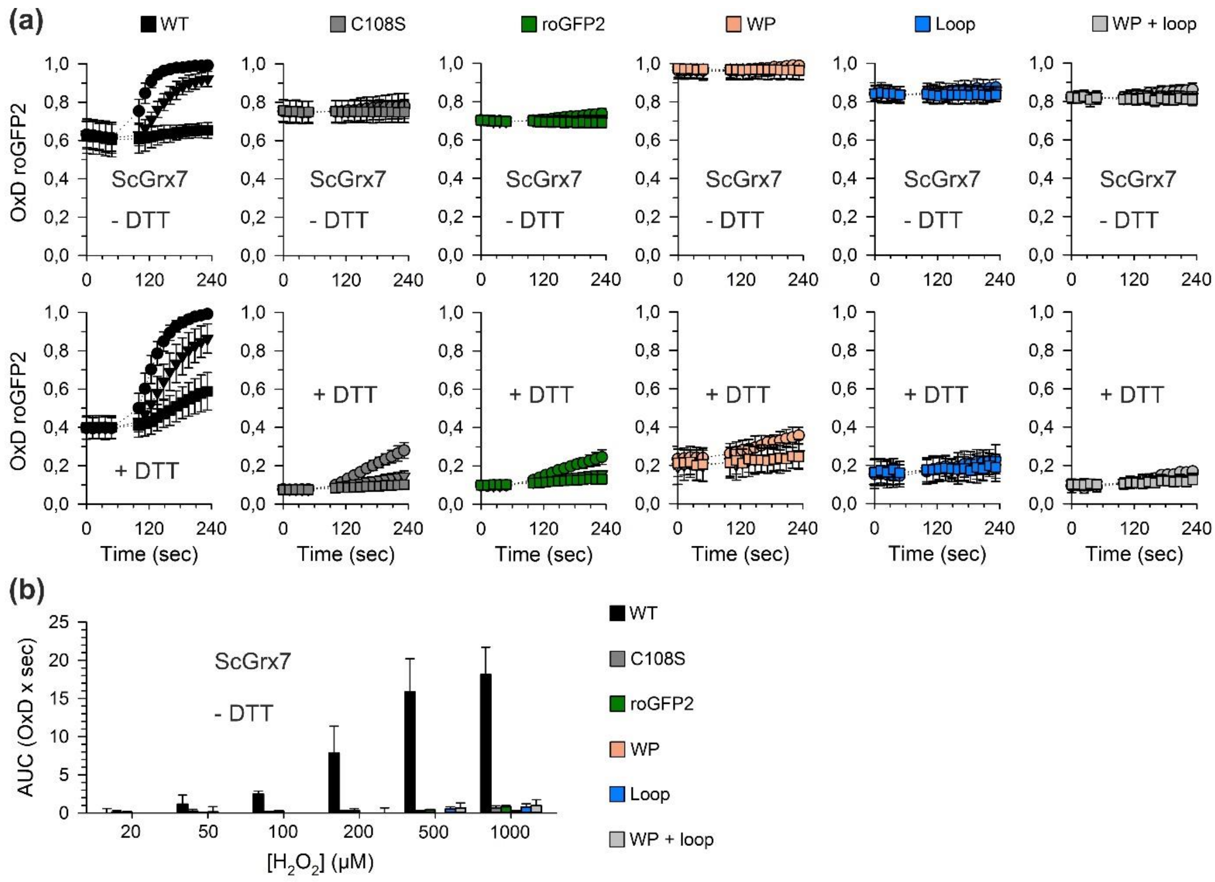

Supplementary Figure 17 | Noninvasive intracellular assessment of ScGrx7 interconversion mutants with or without pretreatment with DTT. (**a**) Time-dependent ratiometric degree of roGFP2 oxidation (OxD) for yeast cells with genetically encoded fusion constructs between roGFP2 and interconversion mutants of ScGrx7 under standard conditions (upper row) or after pretreatment and subsequent washout of DTT (lower row). Representative results for bolus treatments with 1 mM (circles), 0.2 mM (triangles) and 0.05 mM (squares) H_2O_2 are shown. (**b**) Integrated dose-response curves for interconversion mutants ScGrx7^{WP} (WP), ScGrx7^{loop} (Loop) and ScGrx7$^{WP+loop}$ (WP + loop) under standard conditions. The area under the OxD curves was determined for the first 48 seconds following the addition of H_2O_2. RoGFP2 alone (roGFP2) as well fusion constructs with inactive ScGrx7^{C108S} (C108S) or wild-type ScGrx7 (WT) served as negative and positive controls. All experiments were repeated at least three times and data were reported as mean AUCs with error bars representing the standard deviation. Source data are provided as a Source Data file. Statistical analyses and *P*-values are listed in Supplementary Table 12.

Supplementary Figure 18

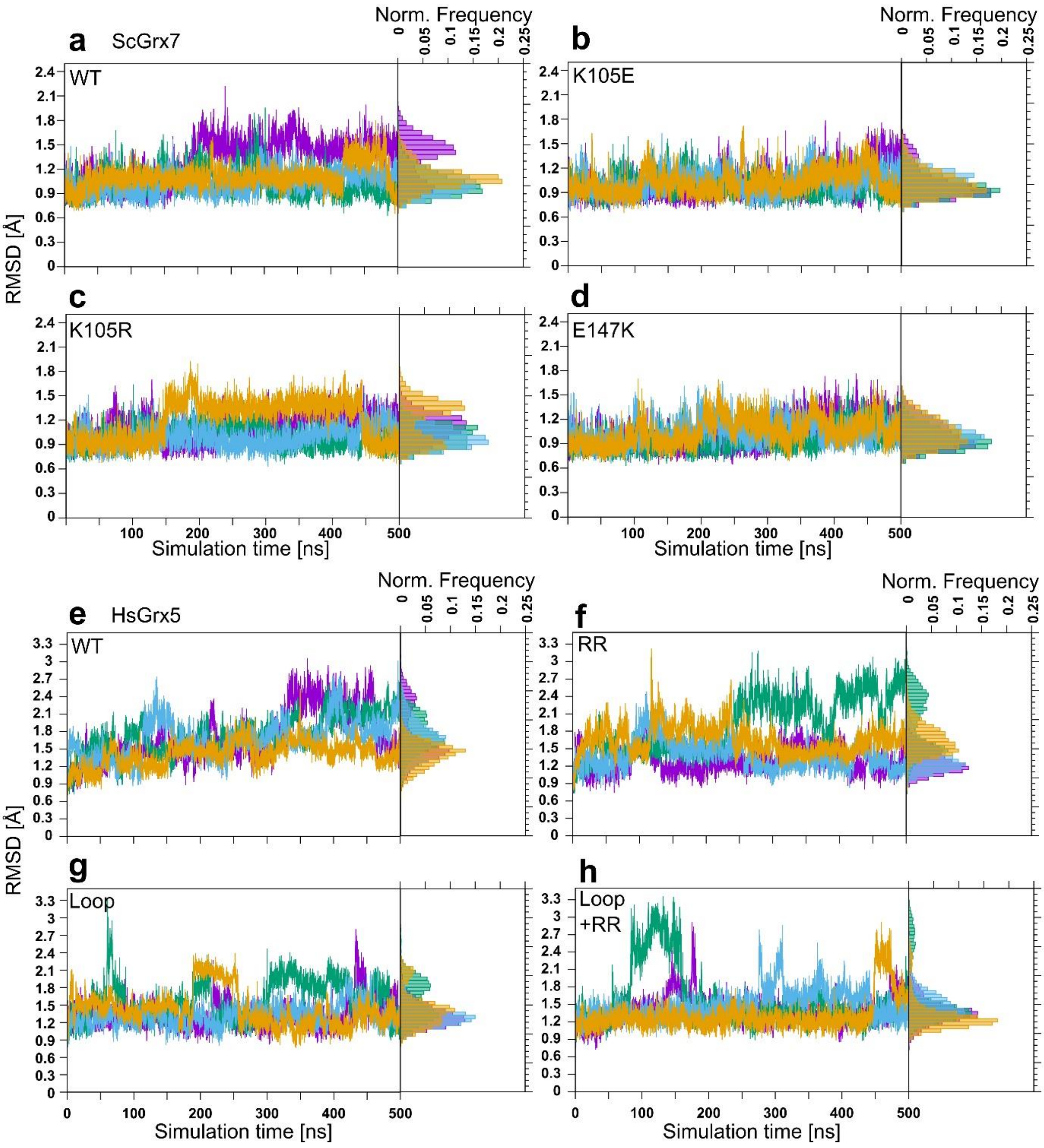

Supplementary Figure 18 | RMSD time series of ScGrx7 and HsGrx5. The RMSD of the peptide backbone atoms of ScGrx7 to the starting structure was calculated for the wild-type ScGrx7 (**a**), the K105E variant (**b**), K105R variant (**c**), and E147K variant (**d**) as well as for HsGrx5 WT (**e**), HsGrx5^{RR} (**f**), HsGrx5^{loop} (**g**) and HsGrx5$^{RR+loop}$ (**h**) over the simulation time of 500 ns. For each protein, the four independent replications of the simulation are colored purple, blue, green, and yellow, respectively. The frequency distributions of the RMSD time series were normalized to a total sum of 1 per replication.

Supplementary Figure 19

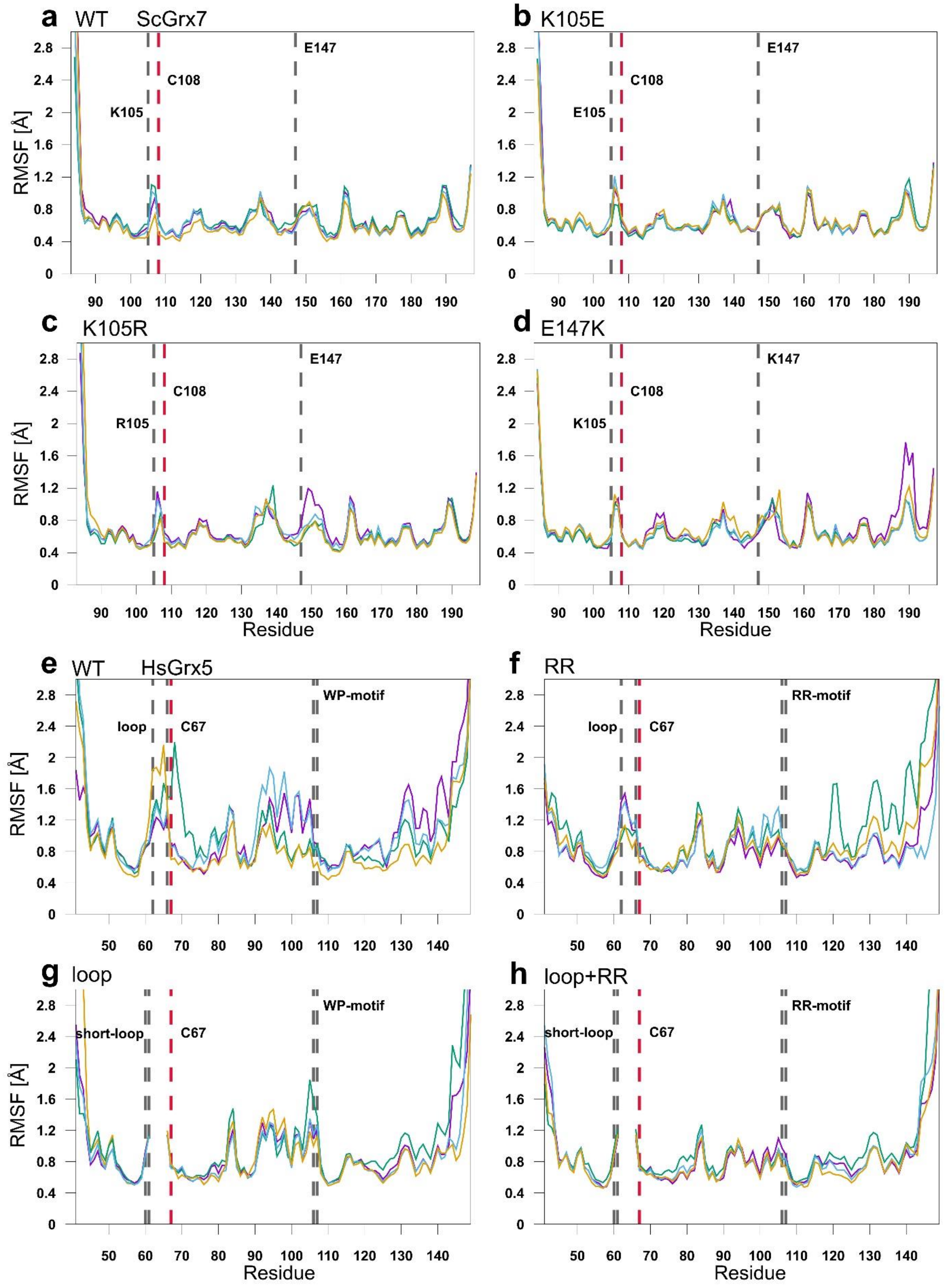

Supplementary Figure 19 | Structural fluctuations of ScGrx7 and HsGrx5. Per-residue root mean square fluctuations (RMSF) of protein backbone atoms over the MD trajectories of

500 ns are shown per replication (purple, blue, green, and yellow) for ScGrx7 WT **(a),** K105E **(b)**, K105R **(c),** and E147K **(d)** as well as for HsGrx5 WT (**e**), HsGrx5^{RR} (**f**), HsGrx5^{loop} (**g**) and HsGrx5$^{RR+loop}$ (**h**). The active cysteine residue is marked with a red line and important positions of the variants are marked with grey lines. The gap in the sequence for short-loop variants of HsGrx5 is plotted to maintain the residue numbering of the WT.

Supplementary Figure 20

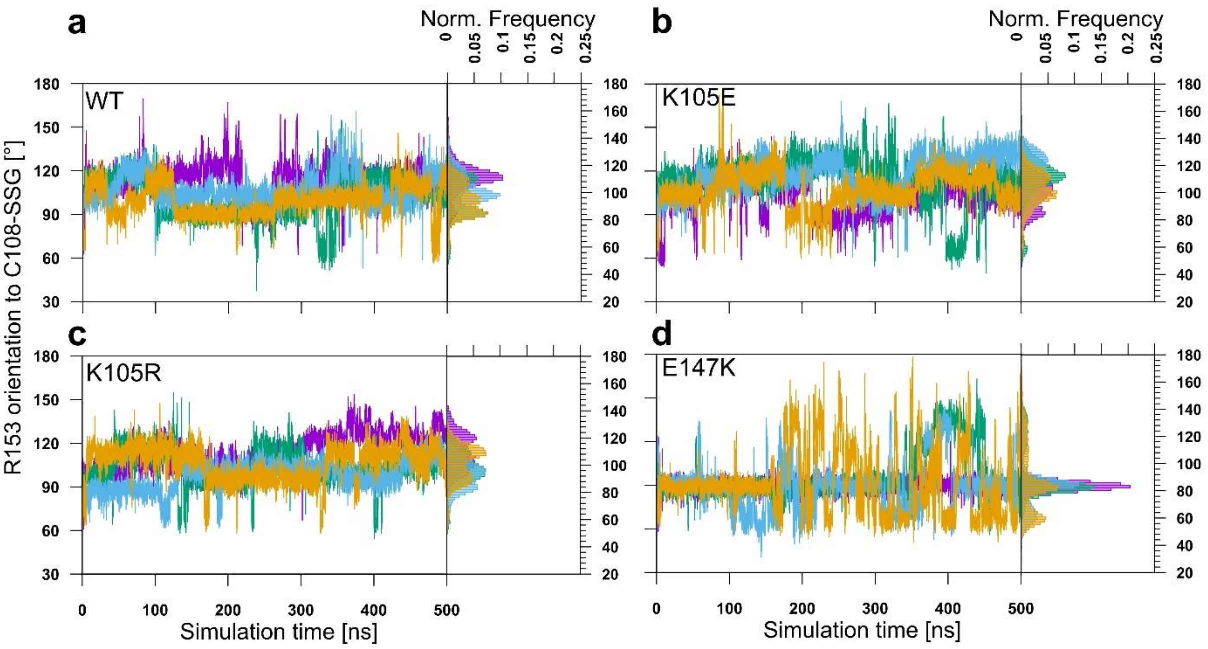

Supplementary Figure 20 | Orientation of R153 in the ScGrx7 variants. The angle between the sulfur atom of the bound glutathione (SSG), the Cα-carbon of R153 and the carbon of the guanidino group of its side chain was chosen to show the differential orientation of R153 towards the SSG moiety. In the WT **(a),** the K105E **(b)** and K105R variants **(c)**, the angle is mainly distributed between 90° and 130°. For the E147K **(d)** variant, the angle predominantly shows a narrow distribution around 90°. For each ScGrx7 variant, the four independent replications of the simulation are colored purple, blue, green, and yellow, respectively. The frequency distributions were normalized to a total sum of 1 per replication.

Supplementary Figure 21

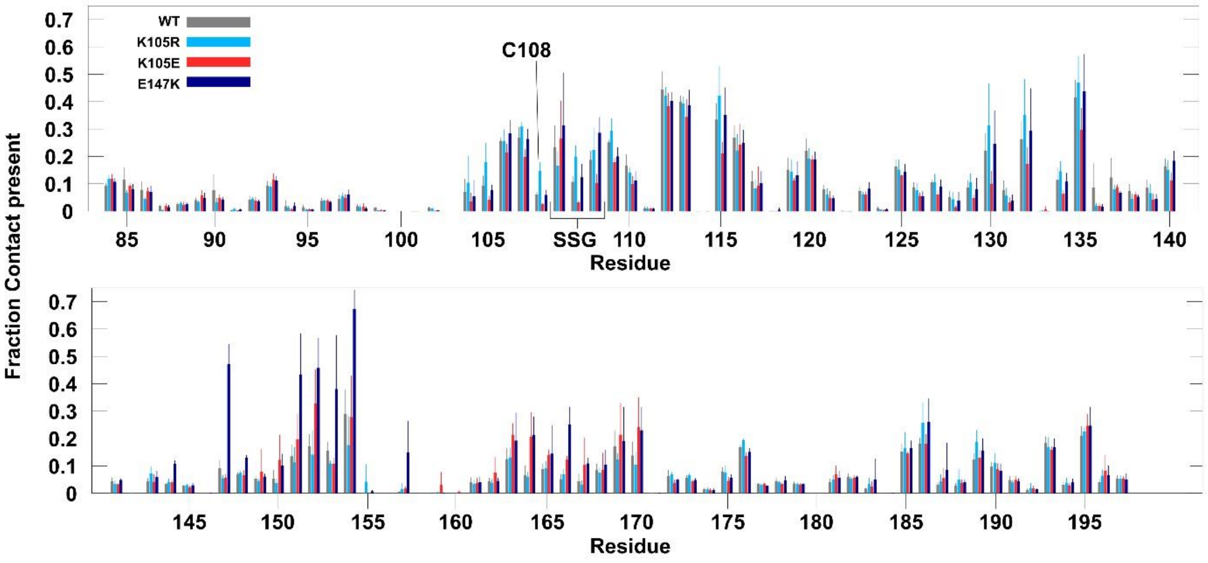

Supplementary Figure 21 | Contact analysis of GS⁻ with ScGrx7. Bars show the fraction of the molecular dynamics simulations where a contact between a residue of ScGrx7 and a freely diffusing GS⁻ was present for the WT (grey), the K105R variant (light blue), K105E variant (red), and E147K variant (dark blue) with error bars showing the standard deviation over the four replications. Contacts were counted when the distance between the Cγ of the glutamyl moiety of a GS⁻ and a specific atom of the residue was below 8 Å. For each residue type, a specific atom of the side chain was selected for the contact definition such that there is no bias against residues with longer side chains. The glutathione disulfide (SSG) bound to Cys108 was not counted in the residue numbering and is represented following Cys108 as three residues: γ-glutamyl, cysteine, and glycine.

Supplementary Figure 22

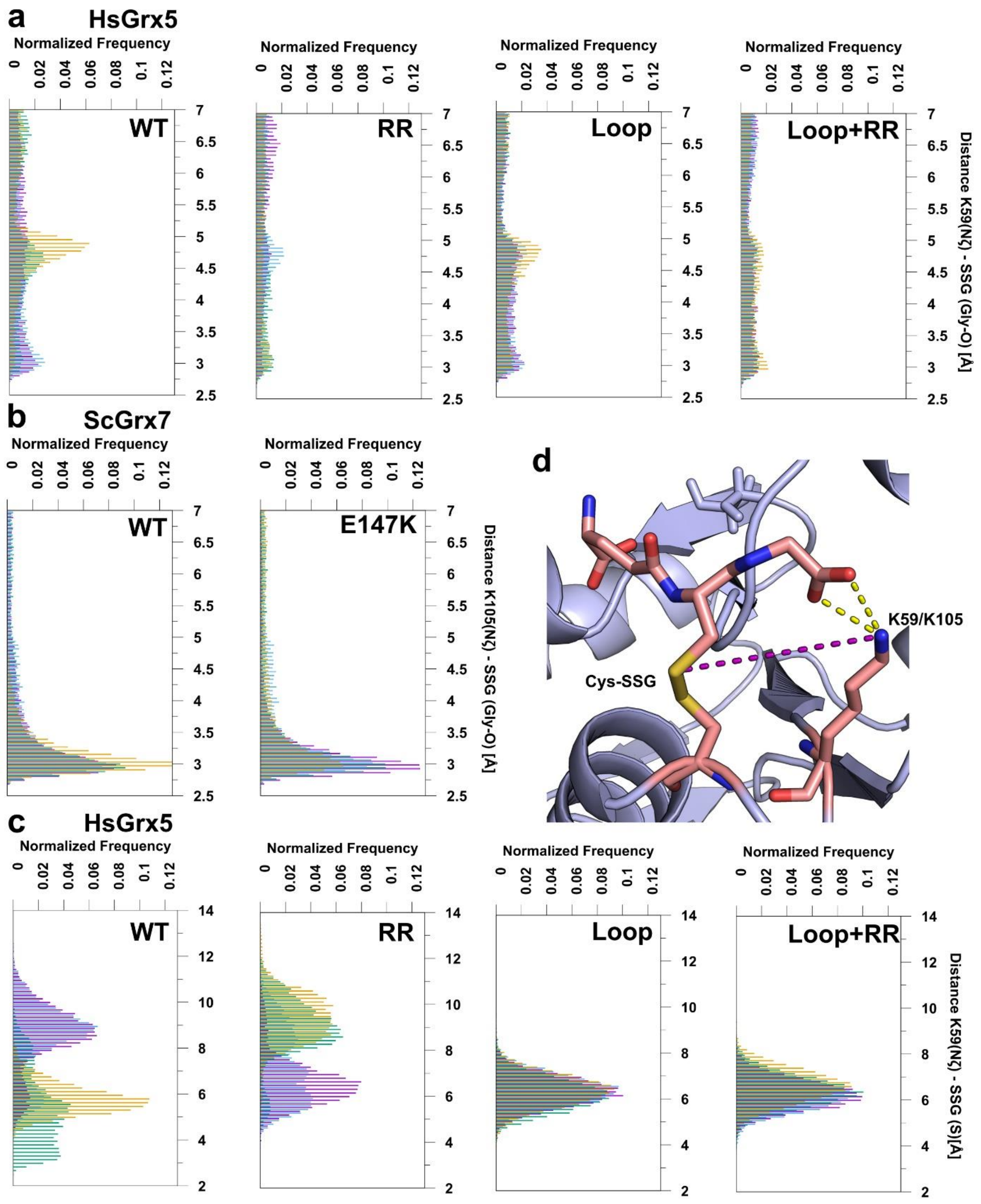

Supplementary Figure 22 | Interactions of the conserved active-site lysine residue in HsGrx5 and ScGrx7. (**a**) Distributions of the distances of Lys59 Nζ of HsGrx5 variants to the closer oxygen atom of the glycine carboxyl group of the covalently bound glutathione for each of the four replications. (**b**) For ScGrx7, the equivalent residue Lys105 was used in the

analysis. (**c**) Distance distributions of Lys59 Nζ to the sulfur atom of bound glutathione. (**d**) Structural representation of the measured distances (yellow for the distance to the carboxyl group as in panels (**a**) and (**b**), purple for the distance as in panel (**c**)).

Supplementary Table 1. Comparison of estimated true k_{cat} and K_m values and Dalziel coefficients Φ_1 and Φ_2 of wild-type ScGrx7 and Y110X mutants in the GSSCys and HEDS assay.

GSSCys assay

**ScGrx7**	**1/Φ1** [a] ($M^{-1}s^{-1}$)	**1/Φ2** [a] ($M^{-1}s^{-1}$)	$k_{cat\,(GSSCys)}$ [a] (s^{-1})	$k_{cat\,(GSH)}$ [a] (s^{-1})	$K_{m\,(GSSCys)}$ [a] (µM)	$K_{m\,(GSH)}$ [a] (µM)
WT	7.2×10^5	2.2×10^5	380	219	292	762
Y110F	7.7×10^5	2.5×10^5	554	346	784	1067
Y110H	1.8×10^5	2.2×10^5	74	154	375	422
Y110A	9.2×10^4	4.7×10^4	35	36	332	426

HEDS assay

**ScGrx7**	**1/Φ1** [b] ($M^{-1}s^{-1}$)	**1/Φ2** [b] ($M^{-1}s^{-1}$)	$k_{cat\,(HEDS)}$ [b] (s^{-1})	$k_{cat\,(GSH)}$ [b] (s^{-1})	$K_{m\,(HEDS)}$ [c] (mM)	$K_{m\,(GSH)}$ [c] (mM)
WT	2.4×10^5	1.5×10^5	185	136	1.3 ± 0.3	1.3 ± 0.8
Y110F	1.2×10^5	1.2×10^5	485	275	1.5 ± 0.2	2.1 ± 0.9
Y110H	2.9×10^4	3.7×10^4	43	92	1.7 ± 0.2	1.8 ± 0.3
Y110A	9.5×10^3	4.6×10^3	30	15	1.4 ± 0.3	0.7 ± 0.2

[a] Estimated Dalziel coefficients, k_{cat} and K_m values were obtained from Supplementary Fig. 3. The true k_{cat} and K_m values have to be interpreted with care because of the proximity of the intersection points to the origin of the graphs.

[b] Estimated Dalziel coefficients and k_{cat} values were obtained from Supplementary Fig. 6a. The true k_{cat} values have to be interpreted with care because of the proximity of the intersection points to the origin of the graphs.

[c] Estimated K_m values were averaged from Supplementary Fig. 5.

Supplementary Table 2. Selected k_{cat}^{app} and K_m^{app} values from GSSCys assays with ScGrx7 wild-type enzyme and Y110X mutants obtained from Supplementary Fig. 2.

ScGrx7	[GSSCys] (µM)	$k_{cat}^{app}{}_{(GSH)}$ [a] (s^{-1})		$K_m^{app}{}_{(GSH)}$ [a] (µM)		$k_{cat}^{app}/K_m^{app}{}_{(GSH)}$ ($mM^{-1}s^{-1}$)	
WT	25	18.8 ± 0.3	100 %	104 ± 7.6	100 %	180	100 %
Y110F	25	17.9 ± 0.2	95 %	68.1 ± 4.2	65 %	264	147 %
Y110H	25	4.8 ± 0.1	26 %	24.8 ± 3.8	24 %	198	110 %
Y110A	25	2.4 ± 0.0	13 %	58.2 ± 4.5	56 %	41	23 %
WT	50	31.1 ± 1.1	100 %	128 ± 16.8	100 %	243	100 %
Y110F	50	36.6 ± 0.5	118 %	141 ± 7.7	110 %	259	107 %
Y110H	50	8.4 ± 0.0	27 %	42.8 ± 0.9	33 %	196	81 %
Y110A	50	4.1 ± 0.1	13 %	100 ± 5.7	78 %	41	17 %
WT	100	52.3 ± 2.2	100 %	215 ± 28.5	100 %	243	100 %
Y110F	100	60.7 ± 1.0	116 %	216 ± 10.8	101 %	280	115 %
Y110H	100	16.9 ± 5.0	32 %	93.0 ± 5.0	43 %	181	74 %
Y110A	100	7.2 ± 0.1	14 %	159 ± 7.1	74 %	46	19 %
WT	150	75.2 ± 1.6	100 %	291 ± 17.6	100 %	258	100 %
Y110F	150	82.5 ± 2.0	110 %	302 ± 20.1	104 %	273	106 %
Y110H	150	21.6 ± 0.3	29 %	105 ± 6.4	36 %	204	79 %
Y110A	150	10.1 ± 0.1	13 %	219 ± 9.6	75 %	46	18 %

ScGrx7	[GSH] (µM)	$k_{cat}^{app}{}_{(GSSCys)}$ [a] (s^{-1})		$K_m^{app}{}_{(GSSCys)}$ [a] (µM)		$k_{cat}^{app}/K_m^{app}{}_{(GSSCys)}$ ($mM^{-1}s^{-1}$)	
WT	50	12.6 ± 0.5	100 %	19.7 ± 3.8	100 %	639	100 %
Y110F	50	13.9 ± 0.03	110 %	19.6 ± 0.2	99 %	712	111 %
Y110H	50	9.4 ± 0.5	75 %	50.4 ± 6.4	256 %	187	29 %
Y110A	50	2.5 ± 0.1	20 %	42.8 ± 6.5	217 %	60	9 %
WT	100	21.2 ± 1.5	100 %	34.2 ± 6.4	100 %	621	100 %
Y110F	100	23.9 ± 1.2	113 %	32.0 ± 5.2	94 %	746	120 %
Y110H	100	17.0 ± 0.6	80 %	90.3 ± 6.1	264 %	188	30 %
Y110A	100	4.1 ± 0.2	19 %	48.9 ± 6.7	143 %	84	14 %
WT	200	40.4 ± 5.2	100 %	64.7 ± 19.7	100 %	624	100 %
Y110F	200	49.1 ± 2.4	122 %	68.6 ± 7.6	106 %	716	115 %
Y110H	200	24.2 ± 1.1	60 %	116 ± 10.3	180 %	207	33 %
Y110A	200	7.5 ± 0.7	19 %	85.9 ± 15.9	133 %	87	14 %
WT	1000	163 ± 54.8	100 %	266 ± 129	100 %	612	100 %
Y110F	1000	142 ± 13.7	87 %	202 ± 28.3	76 %	753	123 %
Y110H	1000	63.1 ± 16.3	39 %	319 ± 113	120 %	198	32 %
Y110A	1000	19.2 ± 1.9	12 %	204 ± 30.7	77 %	94	15 %

[a] Mean ± s.d. from Michaelis-Menten plots of three independent protein purifications. Percentages are relative to wild-type enzyme. *P*-values are listed in Supplementary Table 11 'Statistics'.

Supplementary Table 3. Selected k_{cat}^{app} and K_m^{app} values from HEDS assays with ScGrx7 wild-type enzyme and Y110X mutants obtained from Supplementary Fig. 5.

ScGrx7	[HEDS] (mM)	$k_{cat}^{app}{}_{(GSH)}$ [a] (s^{-1})		$K_m^{app}{}_{(GSH)}$ [a] (mM)		$k_{cat}^{app}/K_m^{app}{}_{(GSH)}$ ($mM^{-1}s^{-1}$)	
WT	0.18	32.1 ± 1.4	100 %	1.55 ± 0.12	100 %	20.7	100 %
Y110F	0.18	19.7 ± 2.5	61 %	1.64 ± 0.38	106 %	12.0	58 %
Y110H	0.18	4.9 ± 0.2	15 %	1.52 ± 0.10	98 %	3.2	15 %
Y110A	0.18	1.6 ± 0.1	5 %	1.64 ± 0.12	106 %	0.9	4 %
WT	0.37	58.6 ± 2.4	100 %	1.60 ± 0.12	100 %	36.7	100 %
Y110F	0.37	39.9 ± 1.0	68 %	1.73 ± 0.08	108 %	23.0	63 %
Y110H	0.37	10.2 ± 0.7	17 %	1.89 ± 0.23	118 %	5.4	15 %
Y110A	0.37	2.6 ± 0.1	4 %	1.07 ± 0.09	67 %	2.5	7 %
WT	0.55	64.6 ± 3.4	100 %	1.14 ± 0.12	100 %	56.6	100 %
Y110F	0.55	52.7 ± 2.8	82 %	1.46 ± 0.14	128 %	36.1	64 %
Y110H	0.55	14.0 ± 0.3	22 %	1.89 ± 0.06	166 %	7.4	13 %
Y110A	0.55	3.6 ± 0.4	6 %	1.25 ± 0.24	110 %	2.9	5 %
WT	0.74	72.9 ± 3.4	100 %	1.10 ± 0.10	100 %	66.5	100 %
Y110F	0.74	63.4 ± 4.9	87 %	1.32 ± 0.19	120 %	48.0	72 %
Y110H	0.74	16.0 ± 1.1	22 %	1.64 ± 0.18	149 %	9.7	15 %
Y110A	0.74	5.6 ± 0.2	8 %	1.52 ± 0.12	138 %	3.7	6 %

ScGrx7	[GSH] (mM)	$k_{cat}^{app}{}_{(HEDS)}$ [a] (s^{-1})		$K_m^{app}{}_{(HEDS)}$ [a] (mM)		$k_{cat}^{app}/K_m^{app}{}_{(HEDS)}$ ($mM^{-1}s^{-1}$)	
WT	0.3	34.1 ± 4.1	100 %	0.92 ± 0.18	100 %	36.9	100 %
Y110F	0.3	31.2 ± 15.8	91 %	1.78 ± 1.20	193 %	17.5	47 %
Y110H	0.3	10.2 ± 1.9	30 %	2.27 ± 0.53	245 %	4.5	12 %
Y110A	0.3	1.7 ± 0.3	5 %	1.10 ± 0.33	120 %	1.5	4 %
WT	0.5	63.9 ± 4.1	100 %	1.33 ± 0.12	100 %	47.8	100 %
Y110F	0.5	78.5 ± 31	123 %	3.40 ± 1.58	256 %	23.1	48 %
Y110H	0.5	13.8 ± 1.6	22 %	2.02 ± 0.30	152 %	6.9	14 %
Y110A	0.5	2.0 ± 0.3	3 %	0.67 ± 0.18	50 %	3.0	6 %
WT	1.0	81.1 ± 8.9	100 %	0.90 ± 0.16	100 %	90.1	100 %
Y110F	1.0	104.4 ± 19.4	129 %	2.59 ± 0.59	288 %	40.2	45 %
Y110H	1.0	20.4 ± 0.8	25 %	1.65 ± 0.15	183 %	12.3	14 %
Y110A	1.0	4.5 ± 0.9	6 %	0.79 ± 0.26	88 %	5.8	6 %
WT	1.5	90.0 ± 12.5	100 %	0.82 ± 0.19	100 %	110	100 %
Y110F	1.5	138.9 ± 28.9	154 %	2.85 ± 0.71	348 %	48.8	44 %
Y110H	1.5	25.8 ± 3.6	29 %	1.77 ± 0.32	216 %	14.6	13 %
Y110A	1.5	4.2 ± 0.1	5 %	0.61 ± 0.02	74 %	6.8	6 %

[a] Mean ± s.d. from Michaelis-Menten plots of three independent protein purifications. Percentages are relative to wild-type enzyme. *P*-values are listed in Supplementary Table 11 'Statistics'.

Supplementary Table 4. Comparison of estimated true k_{cat} and K_m values and Dalziel coefficients Φ_1 and Φ_2 of wild-type ScGrx7 and D144X, E147X and R153X mutants.

GSSCys assay							
**ScGrx7**	**1/Φ_1** [a] ($M^{-1}s^{-1}$)	**1/Φ_2** [a] ($M^{-1}s^{-1}$)	$k_{cat\ (GSSCys)}$ [a] (s^{-1})	$k_{cat\ (GSH)}$ [a] (s^{-1})	$K_{m\ (GSSCys)}$ [a] (µM)	$K_{m\ (GSH)}$ [a] (µM)
WT	7.1×10^5	2.8×10^5	213	206	257	744
D144A	5.7×10^5	3.0×10^5	161	151	250	488
D144K	5.9×10^5	4.7×10^5	591	161	760	590
E147A	7.1×10^5	2.9×10^5	259	370	314	926
E147K	7.6×10^5	7.0×10^5	263	332	280	300
R153A	2.8×10^5	2.7×10^5	125	168	404	293
R153E	1.4×10^5	1.4×10^5	72	113	491	807

[a] Estimated Dalziel coefficients, k_{cat} and K_m values were obtained from Supplementary Fig. 8 (D144X), Supplementary Fig. 10 (E147X) and Supplementary Fig. 12 (R153X). The true k_{cat} and K_m values have to be interpreted with care because of the proximity of the intersection points to the origin of the graphs.

Supplementary Table 5. Selected k_{cat}^{app} and K_m^{app} values from GSSCys assays with ScGrx7 wild-type enzyme and D144X and E147X mutants obtained from Supplementary Figs. 7 and 9.

ScGrx7	[GSSCys] (µM)	$k_{cat}^{app}{}_{(GSH)}$ [a] (s^{-1})		$K_m^{app}{}_{(GSH)}$ [a] (µM)		$k_{cat}^{app}/K_m^{app}{}_{(GSH)}$ (mM^{-1}s^{-1})	
WT	25	16.2 ± 0.2	100 %	67.5 ± 4.4	100 %	240	100 %
D144A	25	13.0 ± 0.1	80 %	68.6 ± 2.6	102 %	190	79 %
D144K	25	10.9 ± 0.1	67 %	25.2 ± 1.2	37 %	433	180 %
E147A	25	17.0 ± 0.1	105 %	77.6 ± 2.3	115 %	219	91 %
E147K	25	18.0 ± 0.3	111 %	47.4 ± 4.9	70 %	379	158 %
WT	50	30.4 ± 0.4	100 %	120 ± 5.6	100 %	253	100 %
D144A	50	23.1 ± 0.2	76 %	103 ± 4.2	86 %	225	89 %
D144K	50	24.9 ± 0.4	82 %	64.0 ± 5.6	53 %	389	154 %
E147A	50	32.3 ± 0.5	106 %	145 ± 0.5	121 %	223	88 %
E147K	50	34.4 ± 0.6	113 %	87.2 ± 6.5	73 %	395	156 %
WT	100	53.3 ± 0.9	100 %	211 ± 11.1	100 %	253	100 %
D144A	100	40.0 ± 1.0	75 %	194 ± 16.4	92 %	214	85 %
D144K	100	42.2 ± 0.7	79 %	82.6 ± 6.2	38 %	511	202 %
E147A	100	60.4 ± 1.0	113 %	237 ± 12.4	112 %	255	101 %
E147K	100	67.3 ± 0.8	126 %	139 ± 6.3	66 %	486	192 %
WT	150	68.0 ± 2.4	100 %	244 ± 27.1	100 %	279	100 %
D144A	150	58.7 ± 1.3	86 %	275 ± 18.1	113 %	214	77 %
D144K	150	58.2 ± 1.7	86 %	138 ± 13.1	56 %	422	151 %
E147A	150	82.9 ± 1.3	122 %	344 ± 14.0	141 %	241	86 %
E147K	150	77.2 ± 1.4	114 %	142 ± 9.2	58 %	543	195 %

ScGrx7	[GSH] (µM)	$k_{cat}^{app}{}_{(GSSCys)}$ [a] (s^{-1})		$K_m^{app}{}_{(GSSCys)}$ [a] (µM)		$k_{cat}^{app}/K_m^{app}{}_{(GSSCys)}$ (mM^{-1}s^{-1})	
WT	50	13.3 ± 0.5	100 %	22.1 ± 3.3	100 %	603	100 %
D144A	50	16.0 ± 0.8	120 %	44.4 ± 6.0	201 %	360	60 %
D144K	50	22.7 ± 0.8	171 %	50.2 ± 4.9	227 %	453	75 %
E147A	50	13.6 ± 0.4	102 %	25.8 ± 2.5	117 %	526	87 %
E147K	50	30.9 ± 1.3	232 %	60.9 ± 6.0	276 %	508	84 %
WT	100	23.2 ± 0.7	100 %	36.4 ± 3.1	100 %	639	100 %
D144A	100	26.2 ± 0.9	112 %	62.7 ± 5.3	172 %	418	65 %
D144K	100	41.3 ± 4.4	178 %	89.2 ± 19.5	245 %	463	72 %
E147A	100	25.8 ± 1.8	111 %	41.1 ± 8.0	113 %	629	98 %
E147K	100	57.5 ± 2.2	248 %	104 ± 7.9	287 %	550	86 %
WT	200	45.8 ± 2.6	100 %	70.7 ± 9.0	100 %	648	100 %
D144A	200	42.3 ± 4.4	92 %	89.3 ± 19.1	126 %	473	73 %
D144K	200	95.2 ± 5.3	208 %	213 ± 18.1	301 %	446	69 %
E147A	200	45.0 ± 1.4	98 %	70.4 ± 4.9	100 %	640	99 %
E147K	200	77.9 ± 3.3	170 %	117 ± 9.2	165 %	665	103 %
WT	1000	114 ± 13.0	100 %	155 ± 29.9	100 %	735	100 %
D144A	1000	122 ± 12.5	107 %	252 ± 37.3	163 %	485	66 %
D144K	1000	182 ± 37.1	160 %	377 ± 103	243 %	483	66 %
E147A	1000	139 ± 7.3	122 %	189 ± 15.6	122 %	737	100 %
E147K	1000	363 ± 33.2	318 %	509 ± 54.2	328 %	714	97 %

[a] Mean ± s.d. from Michaelis-Menten plots of three independent protein purifications. Percentages are relative to wild-type enzyme. *P*-values are listed in Supplementary Table 11 'Statistics'.

Supplementary Table 6. Selected k_{cat}^{app} and K_m^{app} values from GSSCys assays with ScGrx7 wild-type enzyme and R153X mutants obtained from Supplementary Fig. 11.

**ScGrx7**	**[GSSCys]** (µM)	$k_{cat}^{app}{}_{(GSH)}$ [a] (s^{-1})		$K_m^{app}{}_{(GSH)}$ [a] (µM)		$k_{cat}^{app}/K_m^{app}{}_{(GSH)}$ ($mM^{-1}s^{-1}$)	
WT	25	16.2 ± 0.2	100 %	67.5 ± 4.4	100 %	240	100 %
R153A	25	6.6 ± 0.1	41 %	32.8 ± 2.0	49 %	201	84 %
R153E	25	3.5 ± 0.0	22 %	27.1 ± 2.8	40 %	128	53 %
WT	50	30.4 ± 0.4	100 %	120 ± 5.6	100 %	253	100 %
R153A	50	12.9 ± 0.1	42 %	56.2 ± 2.7	47 %	230	91 %
R153E	50	6.6 ± 0.1	22 %	54.3 ± 3.4	45 %	122	48 %
WT	100	53.3 ± 0.9	100 %	211 ± 11.1	100 %	253	100 %
R153A	100	24.2 ± 0.3	45 %	110 ± 4.8	52 %	221	87 %
R153E	100	13.4 ± 0.1	25 %	101 ± 4.3	48 %	133	53 %
WT	150	68.0 ± 2.4	100 %	244 ± 27.1	100 %	279	100 %
R153A	150	31.6 ± 0.6	46 %	118 ± 8.0	48 %	268	96 %
R153E	150	17.0 ± 0.2	25 %	130 ± 6.2	53 %	131	47 %

**ScGrx7**	**[GSH]** (µM)	$k_{cat}^{app}{}_{(GSSCys)}$ [a] (s^{-1})		$K_m^{app}{}_{(GSSCys)}$ [a] (µM)		$k_{cat}^{app}/K_m^{app}{}_{(GSSCys)}$ ($mM^{-1}s^{-1}$)	
WT	50	13.3 ± 0.5	100 %	22.1 ± 3.3	100 %	603	100 %
R153A	50	14.1 ± 0.3	106 %	66.5 ± 3.5	306 %	212	35 %
R153E	50	8.1 ± 1.5	61 %	64.1 ± 27.3	290 %	126	21 %
WT	100	23.2 ± 0.7	100 %	36.4 ± 3.1	100 %	639	100 %
R153A	100	22.5 ± 1.2	97 %	88.5 ± 9.5	243 %	254	40 %
R153E	100	11.9 ± 2.0	51 %	82.7 ± 30.3	227 %	144	23 %
WT	200	45.8 ± 2.6	100 %	70.7 ± 9.0	100 %	648	100 %
R153A	200	37.1 ± 1.2	81 %	135 ± 7.6	191 %	276	43 %
R153E	200	19.5 ± 2.6	43 %	136 ± 32.2	192 %	144	22 %
WT	1000	114 ± 13.0	100 %	155 ± 29.9	100 %	735	100 %
R153A	1000	79.0 ± 5.8	69 %	270 ± 28.2	174 %	293	40 %
R153E	1000	52.2 ± 4.1	46 %	332 ± 19.0	214 %	145	20 %

[a] Mean ± s.d. from Michaelis-Menten plots of three independent protein purifications. Percentages are relative to wild-type enzyme. *P*-values are listed in Supplementary Table 11 'Statistics'.

Supplementary Table 7. Comparison of estimated true k_{cat} and K_m values and Dalziel coefficients Φ_1 and Φ_2 of wild-type ScGrx7 ScGrx7^{WP}, ScGrx7^{loop}, ScGrx7$^{WP+loop}$ as well as HsGrx5, HsGrx5^{RR}, HsGrx5^{loop} and HsGrx5$^{RR+loop}$.

GSSCys assay							
**ScGrx7**	**1/Φ₁** [a] (M^{-1}s^{-1})	**1/Φ₂** [a] (M^{-1}s^{-1})	$k_{cat\ (GSSCys)}$ [a] (s^{-1})	$k_{cat\ (GSH)}$ [a] (s^{-1})	$K_{m\ (GSSCys)}$ [a] (µM)	$K_{m\ (GSH)}$ [a] (µM)
WT	7.8 x 10^5	3.3 x 10^5	492	334	427	1479
RR→WP	1.7 x 10^5	2.8 x 10^5	312	496	2953	1134
loop	5.8 x 10^3	1.9 x 10^3	1.9	1.5	262	1013
WP+loop	1.1 x 10^3	1.5 x 10^3	0.4	0.7	694	265

GSSCys assay						
**HsGrx5** [c]	**1/Φ₁** [b] (M^{-1}s^{-1})	**1/Φ₂** [b] (M^{-1}s^{-1})	$k_{cat\ (GSSCys)}$ [b] (s^{-1})	$k_{cat\ (GSH)}$ [b] (s^{-1})	$K_{m\ (GSSCys)}$ [b] (µM)	$K_{m\ (GSH)}$ [b] (mM)
WT	/	/	/	/	/	/
WP→RR	/	/	/	/	/	/
loop	1.8 x 10^3	4.6 x 10^3	∞	∞	∞	∞
RR+loop	74	/	/	/	/	/

[a] Estimated Dalziel coefficients, k_{cat} and K_m values were obtained from Supplementary Fig. 14. The true k_{cat} and K_m values have to be interpreted with care because of the proximity of the intersection points to the origin of the graphs.

[b] Estimated Dalziel coefficients, k_{cat} and K_m values were obtained from Supplementary Fig. 16. The true k_{cat} values have to be interpreted with care because of the proximity of the intersection points to the origin of the graphs.

[c] Except for HsGrx5^{loop}, the activities of the HsGrx5 constructs were very similar to the NADPH consumption of negative controls (with an activity tendency HsGrx5$^{RR+loop}$ > HsGrx5 > HsGrx5^{RR}).

Supplementary Table 8. Selected k_{cat}^{app} and K_m^{app} values from GSSCys assays with wild-type ScGrx7, ScGrx7^{WP}, ScGrx7^{loop} and ScGrx7$^{WP+loop}$ obtained from Supplementary Fig. 13.

**ScGrx7**	**[GSSCys]** (µM)	$k_{cat}^{app}$$_{(GSH)}$ [a] (s^{-1})		$K_m^{app}$$_{(GSH)}$ [a] (µM)		$k_{cat}^{app}/K_m^{app}$$_{(GSH)}$ (mM^{-1}s^{-1})	
WT	25	18.4 ± 1.1	100 %	78.5 ± 34	100 %	275	100 %
RR→WP	25	4.18 ± 0.66	23 %	32.6 ± 11	42 %	138	50 %
loop	25	0.14 ± 0.01	0.8 %	89.0 ± 9.7	113 %	1.5	0.6 %
WP+loop	25	0.025 ± 0.006	0.1 %	23.2 ± 4.8	30 %	1.1	0.4 %
WT	50	35.4 ± 1.0	100 %	126 ± 42	100 %	308	100 %
RR→WP	50	8.12 ± 0.66	23 %	50.6 ± 9.4	40 %	163	53 %
loop	50	0.22 ± 0.03	0.6 %	158 ± 8.8	125 %	1.4	0.5 %
WP+loop	50	0.05 ± 0.015	0.1 %	46.9 ± 2.0	37 %	1.1	0.3 %
WT	100	67.4 ± 3.9	100 %	222 ± 9.4	100 %	305	100 %
RR→WP	100	15.7 ± 1.4	23 %	81.4 ± 17	37 %	197	65 %
loop	100	0.42 ± 0.11	0.6 %	247 ± 59	111 %	1.7	0.6 %
WP+loop	100	0.098 ± 0.021	0.1 %	81.2 ± 25	37 %	1.2	0.4 %
WT	150	79.9 ± 27	100 %	286 ± 45	100 %	273	100 %
RR→WP	150	26.0 ± 2.0	33 %	124 ± 25	43 %	214	78 %
loop	150	0.65 ± 0.14	0.8 %	350 ± 25	122 %	1.8	0.7 %
WP+loop	150	0.12 ± 0.02	0.2 %	87.0 ± 21	30 %	1.5	0.5 %

**ScGrx7**	**[GSH]** (µM)	$k_{cat}^{app}$$_{(GSSCys)}$ [a] (s^{-1})		$K_m^{app}$$_{(GSSCys)}$ [a] (µM)		$k_{cat}^{app}/K_m^{app}$$_{(GSSCys)}$ (mM^{-1}s^{-1})	
WT	50	22.0 ± 1.3	100 %	49.6 ± 15	100 %	475	100 %
RR→WP	50	13.2 ± 1.1	60 %	100 ± 6.1	202 %	132	28 %
loop	50	0.11 ± 0.01	0.5 %	30.7 ± 6.0	62 %	3.8	0.8 %
WP+loop	50	0.066 ± 0.02	0.3 %	69.4 ± 13	140 %	1.0	0.2 %
WT	200	56.0 ± 7.9	100 %	84.0 ± 28	100 %	696	100 %
RR→WP	200	41.2 ± 5.6	74 %	281 ± 43	335 %	147	21 %
loop	200	0.31 ± 0.06	0.6 %	76.4 ± 3.8	91 %	4.0	0.6 %
WP+loop	200	0.13 ± 0.04	0.2 %	150 ± 40	179 %	0.9	0.1 %
WT	500	139 ± 20	100 %	193 ± 37	100 %	727	100 %
RR→WP	500	128 ± 36	92 %	750 ± 190	389 %	170	23 %
loop	500	0.61 ± 0.11	0.4 %	120 ± 34	62 %	5.3	0.7 %
WP+loop	500	0.25 ± 0.02	0.2 %	242 ± 36	125 %	1.0	0.1 %
WT	1000	239 ± 54	100 %	320 ± 91	100 %	757	100 %
RR→WP	1000	175 ± 51	73 %	1036 ± 377	324 %	173	23 %
loop	1000	0.94 ± 0.38	0.4 %	170 ± 5.8	53 %	5.5	0.7 %
WP+loop	1000	0.69 ± 0.12	0.3 %	631 ± 38	197 %	1.1	0.1 %

[a] Mean ± s.d. from Hanes plots of three independent protein purifications. Percentages are relative to wild-type enzyme. *P*-values are listed in Supplementary Table 11 'Statistics'.

Supplementary Table 9. Selected k_{cat}^{app} and K_m^{app} values from GSSCys assays with HsGrx5, $HsGrx5^{RR}$, $HsGrx5^{loop}$ and $HsGrx5^{RR+loop}$ obtained from Supplementary Fig. 15.

HsGrx5[b]	[GSSCys] (µM)	$k_{cat}^{app}{}_{(GSH)}$ [a] (s^{-1})		$K_m^{app}{}_{(GSH)}$ [a] (µM)		$k_{cat}^{app}/K_m^{app}{}_{(GSH)}$ ($mM^{-1}s^{-1}$)	
WT	25	/	0 %	/	0 %	<0.1	<2 %
WP→RR	25	/	0 %	/	0 %	/	0 %
loop	25	0.046 ± 0.008	100 %	10.2 ± 5.3	100 %	5.7	100 %
RR+loop	25	/	0 %	/	0 %	<0.1	<2 %
WT	50	0.004 ± 0.002	<5 %	/	0 %	<0.2	<4 %
WP→RR	50	/	0 %	/	0 %	/	0 %
loop	50	0.083 ± 0.012	100 %	17.7 ± 8.9	100 %	5.4	100 %
RR+loop	50	0.004 ± 0.003	5 %	25.4 ± 47	144 %	0.1	1 %
WT	100	0.003 ± 0.002	<2 %	/	0 %	<0.2	<5 %
WP→RR	100	/	0 %	/	0 %	/	0 %
loop	100	0.18 ± 0.05	100 %	47.6 ± 30	100 %	3.9	100 %
RR+loop	100	0.016 ± 0.013	9 %	127 ± 87	267 %	0.1	3 %
WT	150	0.003 ± 0.002	<1 %	/	0 %	<0.2	<4 %
WP→RR	150	/	0 %	/	0 %	/	0 %
loop	150	0.27 ± 0.02	100 %	59.9 ± 15	100 %	4.8	100 %
RR+loop	150	0.019 ± 0.007	7 %	174 ± 91	290 %	0.1	3 %

HsGrx5[b]	[GSH] (µM)	$k_{cat}^{app}{}_{(GSSCys)}$ [a] (s^{-1})		$K_m^{app}{}_{(GSSCys)}$ [a] (µM)		$k_{cat}^{app}/K_m^{app}{}_{(GSSCys)}$ ($mM^{-1}s^{-1}$)	
WT	50	/	0 %	/	0 %	<0.1	<6 %
WP→RR	50	/	0 %	/	0 %	/	0 %
loop	50	0.24 ± 0.00	100 %	156 ± 19	100 %	1.5	100 %
RR+loop	50	/	0 %	/	0 %	<0.2	<12 %
WT	200	/	0 %	/	0 %	<0.1	<6 %
WP→RR	200	/	0 %	/	0 %	/	0 %
loop	200	0.88 ± 0.06	100 %	563 ± 58	100 %	1.6	100 %
RR+loop	200	/	0 %	/	0 %	<0.2	<12 %
WT	500	/	0 %	/	0 %	<0.2	<12 %
WP→RR	500	/	0 %	/	0 %	/	0 %
loop	500	4.33 ± 0.21	100 %	2703 ± 119	100 %	1.6	100 %
RR+loop	500	/	0 %	/	0 %	<0.2	<12 %
WT	1000	/	0 %	/	0 %	<0.1	<6 %
WP→RR	1000	/	0 %	/	0 %	/	0 %
loop	1000	1.08 ± 6.3	100 %	765 ± 3811	100 %	1.7	100 %
RR+loop	1000	/	0 %	/	0 %	<0.2	<12 %

[a] Mean ± s.d. from Hanes plots of three independent protein purifications. Percentages are relative to $HsGrx5^{loop}$. *P*-values are listed in Supplementary Table 11 'Statistics'.

[b] Except for $HsGrx5^{loop}$, the activities of the HsGrx5 constructs were very similar to the NADPH consumption of negative controls (with an activity tendency $HsGrx5^{RR+loop}$ > HsGrx5 > $HsGrx5^{RR}$).

Supplementary Table 10. List of mutagenesis and subcloning primers.

**Primer**	**Sequence** (codons, mutations and restriction sites highlighted)
ScGrx7/Y110A/s	5’-GCAAGACTGGCTGCCCA*GCT*AGCAAAAAACTGAAAGC-3’
ScGrx7/Y110A/as	5’-GCTTTCAGTTTTTTGCT*AGC*TGGGCAGCCAGTCTTGC-3’
ScGrx7/Y110F/s	5’-GCAAGACTGGCTGCCCA*TTT*AGCAAAAAACTGAAAGC-3’
ScGrx7/Y110F/as	5’-GCTTTCAGTTTTTTGCT*AAA*TGGGCAGCCAGTCTTGC-3’
ScGrx7/Y110H/s	5’-GCAAGACTGGCTGCCCA*CAT*AGCAAAAAACTGAAAGC-3’
ScGrx7/Y110H/as	5’-GCTTTCAGTTTTTTGCT*ATG*TGGGCAGCCAGTCTTGC-3’
ScGrx7/D144A/s	5’-CACACAAAAGAACTACAA*GCC*CAGATTGAAAAAGTCACTGG-3’
ScGrx7/D144A/as	5’-CCAGTGACTTTTTCAATCTG*GGC*TTGTAGTTCTTTTGTGTG-3’
ScGrx7/D144K/s	5’-CACACAAAAGAACTACAA*AAA*CAGATTGAAAAAGTCACTGG-3’
ScGrx7/D144K/as	5’-CCAGTGACTTTTTCAATCTG*TTT*TTGTAGTTCTTTTGTGTG-3’
ScGrx7/D147A/s	5’-GAACTACAAGACCAGATT*GCA*AAAGTCACTGGTAGGAGAAC-3’
ScGrx7/D147A/s	5’-GTTCTCCTACCAGTGACTTT*TGC*AATCTGGTCTTGTAGTTC-3’
ScGrx7/D147K/s	5’-GAACTACAAGACCAGATT*AAA*AAAGTCACTGGTAGGAGAAC-3’
ScGrx7/D147K/as	5’-GTTCTCCTACCAGTGACTTT*TTT*AATCTGGTCTTGTAGTTC-3’
ScGrx7/R153A/s	5’-GAAAAAGTCACTGGTAGG*GCA*ACAGTCCCAAACGTTATCATC-3’
ScGrx7/R153A/as	5’-GATGATAACGTTTGGGACTGT*TGC*CCTACCAGTGACTTTTTC-3’
ScGrx7/R153E/s	5’-GAAAAAGTCACTGGTAGG*GAA*ACAGTCCCAAACGTTATCATC-3’
ScGrx7/R153E/as	5’-GATGATAACGTTTGGGACTGT*TTC*CCTACCAGTGACTTTTTC-3’
ScGrx7/BamHI/s	5’-GATC*GGATCC*GTTAACGAATCAATCACTACACATC-3’
ScGrx7/HindIII/as	5’-GATC*AAGCTT*TCATTAAGCAGATTCTGATTGAGAATTAG-3’
HsGrx5/BamHI/s	5’-GATC*GGATCC*GCTGGTTCTGGTGCTGGTGG-3’
HsGrx5/HindIII/as	5’-GATC*AAGCTT*TCATTATTTTGAATCTTGATCTTTCTTTTC-3’
HsGrx5^{Loop}/G68P/s	5’-GAAGACAGGATGT*CCA*TTTTCTAACGCTG-3’
HsGrx5^{Loop}/G68P/as	5’-CAGCGTTAGAAAA*TGG*ACATCCTGTCTTC-3’
HsGrx5/R97Q/s	5’-GATGATCCAGAATTG*CAA*CAAGGTATTAAAG-3’
HsGrx5/ R97Q /as	5’-CTTTAATACCTTG*TTG*CAATTCTGGATCATC-3’

Supplementary Table 11. Statistical analysis of k_{cat}^{app} and K_m^{app} values from the indicated measurements of ScGrx7. *P*-values from one way ANOVA analysis followed by a Holm-Sidak test were calculated in SigmaPlot 13 (P > 0.05 : ns; P ≤ 0.05 : *; P ≤ 0.01 : **, P ≤ 0.001 : ***).

A) GSSCys assay ScGrx7 Y110X (Fig. 2, Supplementary Fig. 2 and Supplementary Table 2)

A1.1) $k_{cat}^{app}{}_{(GSSCys)}$ @ 50 µM GSH

Comparison		P-value	
WT	→ Y110F	0.019	*
WT	→ Y110H	<0.001	***
WT	→ Y110A	<0.001	***
Y110F	→ Y110H	<0.001	***
Y110F	→ Y110A	<0.001	***
Y110H	→ Y110A	<0.001	***

A1.2) $k_{cat}^{app}{}_{(GSSCys)}$ @ 100 µM GSH

Comparison		P-value	
WT	→ Y110F	0.101	ns
WT	→ Y110H	0.033	*
WT	→ Y110A	<0.001	***
Y110F	→ Y110H	0.004	**
Y110F	→ Y110A	<0.001	***
Y110H	→ Y110A	<0.001	***

A1.3) $k_{cat}^{app}{}_{(GSSCys)}$ @ 200 µM GSH

Comparison		P-value	
WT	→ Y110F	0.070	ns
WT	→ Y110H	0.009	*
WT	→ Y110A	<0.001	***
Y110F	→ Y110H	0.001	***
Y110F	→ Y110A	<0.001	***
Y110H	→ Y110A	0.012	*

A1.4) $k_{cat}^{app}{}_{(GSSCys)}$ @ 1000 µM GSH

Comparison		P-value	
WT	→ Y110F	0.413	ns
WT	→ Y110H	0.013	*
WT	→ Y110A	0.002	**
Y110F	→ Y110H	0.032	*
Y110F	→ Y110A	0.004	**
Y110H	→ Y110A	0.199	ns

A2.1) $k_{cat}^{app}{}_{(GSH)}$ @ 25 µM GSSCys

Comparison		P-value	
WT	→ Y110F	0.017	*
WT	→ Y110H	<0.001	***
WT	→ Y110A	<0.001	***
Y110F	→ Y110H	<0.001	***
Y110F	→ Y110A	<0.001	***
Y110H	→ Y110A	<0.001	***

A2.2) $k_{cat}^{app}{}_{(GSH)}$ @ 50 µM GSSCys

Comparison		P-value	
WT	→ Y110F	<0.001	***
WT	→ Y110H	<0.001	***
WT	→ Y110A	<0.001	***
Y110F	→ Y110H	<0.001	***
Y110F	→ Y110A	<0.001	***
Y110H	→ Y110A	0.001	***

A2.3) $k_{cat}^{app}{}_{(GSH)}$ @ 100 µM GSSCys

Comparison		P-value	
WT	→ Y110F	0.001	***
WT	→ Y110H	<0.001	***
WT	→ Y110A	<0.001	***
Y110F	→ Y110H	<0.001	***
Y110F	→ Y110A	<0.001	***
Y110H	→ Y110A	0.001	***

A2.4) $k_{cat}^{app}{}_{(GSH)}$ @ 150 µM GSSCys

Comparison		P-value	
WT	→ Y110F	<0.001	***
WT	→ Y110H	<0.001	***
WT	→ Y110A	<0.001	***
Y110F	→ Y110H	<0.001	***
Y110F	→ Y110A	0.004	**
Y110H	→ Y110A	<0.001	***

A3.1) $K_m^{app}{}_{(GSSCys)}$ @ 50 µM GSH

Comparison		P-value	
WT	→ Y110F	0.991	ns
WT	→ Y110H	0.011	*
WT	→ Y110A	0.032	*
Y110F	→ Y110H	0.013	*
Y110F	→ Y110A	0.041	*
Y110H	→ Y110A	0.520	ns

A4.1) $K_m^{app}{}_{(GSH)}$ @ 25 µM GSSCys

Comparison		P-value	
WT	→ Y110F	0.004	**
WT	→ Y110H	<0.001	***
WT	→ Y110A	0.001	***
Y110F	→ Y110H	0.001	***
Y110F	→ Y110A	0.223	ns
Y110H	→ Y110A	0.004	**

A3.2) $K_m^{app}{}_{(GSSCys)}$ @ 100 µM GSH

Comparison		P-value	
WT	→ Y110F	0.807	ns
WT	→ Y110H	<0.001	***
WT	→ Y110A	0.237	ns
Y110F	→ Y110H	<0.001	***
Y110F	→ Y110A	0.236	ns
Y110H	→ Y110A	0.006	**

A4.2) $K_m^{app}{}_{(GSH)}$ @ 50 µM GSSCys

Comparison		P-value	
WT	→ Y110F	0.355	ns
WT	→ Y110H	0.001	***
WT	→ Y110A	0.151	ns
Y110F	→ Y110H	<0.001	***
Y110F	→ Y110A	0.051	ns
Y110H	→ Y110A	0.012	*

A3.3) $K_m^{app}{}_{(GSSCys)}$ @ 200 µM GSH

Comparison		P-value	
WT	→ Y110F	0.747	ns
WT	→ Y110H	0.012	*
WT	→ Y110A	0.283	ns
Y110F	→ Y110H	0.016	*
Y110F	→ Y110A	0.317	ns
Y110H	→ Y110A	0.111	ns

A4.3) $K_m^{app}{}_{(GSH)}$ @ 100 µM GSSCys

Comparison		P-value	
WT	→ Y110F	0.949	ns
WT	→ Y110H	0.003	**
WT	→ Y110A	0.072	ns
Y110F	→ Y110H	0.003	**
Y110F	→ Y110A	0.096	ns
Y110H	→ Y110A	0.071	ns

A3.4) $K_m^{app}{}_{(GSSCys)}$ @ 1000 µM GSH

Comparison		P-value	
WT	→ Y110F	0.114	ns
WT	→ Y110H	0.672	ns
WT	→ Y110A	0.214	ns
Y110F	→ Y110H	0.310	ns
Y110F	→ Y110A	0.667	ns
Y110H	→ Y110A	0.379	ns

A4.4) $K_m^{app}{}_{(GSH)}$ @ 150 µM GSSCys

Comparison		P-value	
WT	→ Y110F	0.608	ns
WT	→ Y110H	<0.001	***
WT	→ Y110A	0.017	*
Y110F	→ Y110H	<0.001	***
Y110F	→ Y110A	0.012	*
Y110H	→ Y110A	0.002	**

B) HEDS assay ScGrx7 Y110X (Supplementary Figs. 4 and 5 and Supplementary Table 3)

B1.1) $k_{cat}^{app}{}_{(HEDS)}$ @ 300 µM GSH

Comparison		P-value	
WT	→ Y110F	0.596	ns
WT	→ Y110H	0.007	**
WT	→ Y110A	0.001	***
Y110F	→ Y110H	0.011	*
Y110F	→ Y110A	0.002	**
Y110H	→ Y110A	0.262	ns

B1.2) $k_{cat}^{app}{}_{(HEDS)}$ @ 500 µM GSH

Comparison		P-value	
WT	→ Y110F	0.223	ns
WT	→ Y110H	<0.001	***
WT	→ Y110A	<0.001	***
Y110F	→ Y110H	<0.001	***
Y110F	→ Y110A	<0.001	***
Y110H	→ Y110A	0.196	ns

B1.3) $k_{cat}^{app}{}_{(HEDS)}$ @ 1000 µM GSH

Comparison		P-value	
WT	→ Y110F	0.298	ns
WT	→ Y110H	0.011	*
WT	→ Y110A	0.004	**
Y110F	→ Y110H	0.003	**
Y110F	→ Y110A	0.001	***
Y110H	→ Y110A	0.325	ns

B1.4) $k_{cat}^{app}{}_{(HEDS)}$ @ 1500 µM GSH

Comparison		P-value	
WT	→ Y110F	0,033	*
WT	→ Y110H	0,012	*
WT	→ Y110A	0.003	**
Y110F	→ Y110H	<0.001	***
Y110F	→ Y110A	<0.001	***
Y110H	→ Y110A	0.218	ns

B2.1) $k_{cat}^{app}{}_{(GSH)}$ @ 0.18 mM HEDS

Comparison		P-value	
WT	→ Y110F	<0.001	***
WT	→ Y110H	<0.001	***
WT	→ Y110A	<0.001	***
Y110F	→ Y110H	<0.001	***
Y110F	→ Y110A	<0.001	***
Y110H	→ Y110A	0.139	ns

B2.2) $k_{cat}^{app}{}_{(GSH)}$ @ 0.37 mM HEDS

Comparison		P-value	
WT	→ Y110F	<0.001	***
WT	→ Y110H	<0.001	***
WT	→ Y110A	<0.001	***
Y110F	→ Y110H	<0.001	***
Y110F	→ Y110A	<0.001	***
Y110H	→ Y110A	0.005	**

B2.3) $k_{cat}^{app}{}_{(GSH)}$ @ 0.55 mM HEDS

Comparison		P-value	
WT	→ Y110F	0.010	**
WT	→ Y110H	<0.001	***
WT	→ Y110A	<0.001	***
Y110F	→ Y110H	<0.001	***
Y110F	→ Y110A	<0.001	***
Y110H	→ Y110A	0.010	**

B2.4) $k_{cat}^{app}{}_{(GSH)}$ @ 0.74 mM HEDS

Comparison		P-value	
WT	→ Y110F	0.057	ns
WT	→ Y110H	<0.001	***
WT	→ Y110A	<0.001	***
Y110F	→ Y110H	<0.001	***
Y110F	→ Y110A	<0.001	***
Y110H	→ Y110A	0.081	ns

B3.1) $K_m^{app}{}_{(HEDS)}$ @ 300 µM GSH

Comparison		P-value	
WT	→ Y110F	0.511	ns
WT	→ Y110H	0.511	ns
WT	→ Y110A	0.511	ns
Y110F	→ Y110H	0.511	ns
Y110F	→ Y110A	0.511	ns
Y110H	→ Y110A	0.511	ns

B3.2) $K_m^{app}{}_{(HEDS)}$ @ 500 µM GSH

Comparison		P-value	
WT	→ Y110F	0.182	ns
WT	→ Y110H	0.182	ns
WT	→ Y110A	0.182	ns
Y110F	→ Y110H	0.182	ns
Y110F	→ Y110A	0.182	ns
Y110H	→ Y110A	0.182	ns

B3.3) $K_m^{app}{}_{(HEDS)}$ @ 1000 µM GSH

Comparison		P-value	
WT	→ Y110F	0.039	*
WT	→ Y110H	0.289	ns
WT	→ Y110A	0.816	ns
Y110F	→ Y110H	0.305	ns
Y110F	→ Y110A	0.033	*
Y110H	→ Y110A	0.293	ns

B3.4) $K_m^{app}{}_{(HEDS)}$ @ 1500 µM GSH

Comparison		P-value	
WT	→ Y110F	0.037	*
WT	→ Y110H	0.250	ns
WT	→ Y110A	0.723	ns
Y110F	→ Y110H	0.261	ns
Y110F	→ Y110A	0.026	*
Y110H	→ Y110A	0.272	ns

B4.1) $K_m^{app}{}_{(GSH)}$ @ 0.18 mM HEDS

Comparison		P-value	
WT	→ Y110F	0.966	ns
WT	→ Y110H	0.966	ns
WT	→ Y110A	0.966	ns
Y110F	→ Y110H	0.966	ns
Y110F	→ Y110A	0.966	ns
Y110H	→ Y110A	0.966	ns

B4.2) $K_m^{app}{}_{(GSH)}$ @ 0.37 mM HEDS

Comparison		P-value	
WT	→ Y110F	0.884	ns
WT	→ Y110H	0.525	ns
WT	→ Y110A	0.159	ns
Y110F	→ Y110H	0.470	ns
Y110F	→ Y110A	0.156	ns
Y110H	→ Y110A	0.022	*

B4.3) $K_m^{app}{}_{(GSH)}$ @ 0.55 mM HEDS

Comparison		P-value	
WT	→ Y110F	0.443	ns
WT	→ Y110H	0.057	ns
WT	→ Y110A	0.621	ns
Y110F	→ Y110H	0.277	ns
Y110F	→ Y110A	0.594	ns
Y110H	→ Y110A	0.084	ns

B4.4) $K_m^{app}{}_{(GSH)}$ @ 0.74 mM HEDS

Comparison		P-value	
WT	→ Y110F	0.146	ns
WT	→ Y110H	0.146	ns
WT	→ Y110A	0.146	ns
Y110F	→ Y110H	0.146	ns
Y110F	→ Y110A	0.146	ns
Y110H	→ Y110A	0.146	ns

C) GSSCys assay ScGrx7 D144X (Fig. 4, Supplementary Fig. 7 and Supplementary Table 5)

C1.1) $k_{cat}^{app}{}_{(GSSCys)}$ @ 50 µM GSH

Comparison		P-value	
WT	→ D144A	0.040	*
WT	→ D144K	<0.001	***
D144A	→ D144K	0.001	***

C1.2) $k_{cat}^{app}{}_{(GSSCys)}$ @ 100 µM GSH

Comparison		P-value	
WT	→ D144A	0.453	ns
WT	→ D144K	0.008	**
D144A	→ D144K	0.013	*

C1.3) $k_{cat}^{app}{}_{(GSSCys)}$ @ 200 µM GSH

Comparison		P-value	
WT	→ D144A	0.576	ns
WT	→ D144K	<0.001	***
D144A	→ D144K	<0.001	***

C1.4) $k_{cat}^{app}{}_{(GSSCys)}$ @ 1000 µM GSH

Comparison		P-value	
WT	→ D144A	0.682	ns
WT	→ D144K	0.038	*
D144A	→ D144K	0.043	*

C3.1) $K_m^{app}{}_{(GSSCys)}$ @ 50 µM GSH

Comparison		P-value	
WT	→ D144A	0.035	*
WT	→ D144K	0.020	*
D144A	→ D144K	0.437	ns

C3.2) $K_m^{app}{}_{(GSSCys)}$ @ 100 µM GSH

Comparison		P-value	
WT	→ D144A	0.034	*
WT	→ D144K	0.005	**
D144A	→ D144K	0.066	ns

C3.3) $K_m^{app}{}_{(GSSCys)}$ @ 200 µM GSH

Comparison		P-value	
WT	→ D144A	0.443	ns
WT	→ D144K	0.002	**
D144A	→ D144K	0.003	**

C3.4) $K_m^{app}{}_{(GSSCys)}$ @ 1000 µM GSH

Comparison		P-value	
WT	→ D144A	0.119	ns
WT	→ D144K	0.018	*
D144A	→ D144K	0.112	ns

C2.1) $k_{cat}^{app}{}_{(GSH)}$ @ 25 µM GSSCys

Comparison		P-value	
WT	→ D144A	<0.001	***
WT	→ D144K	<0.001	***
D144A	→ D144K	<0.001	***

C2.2) $k_{cat}^{app}{}_{(GSH)}$ @ 50 µM GSSCys

Comparison		P-value	
WT	→ D144A	<0.001	***
WT	→ D144K	<0.001	***
D144A	→ D144K	0.012	*

C2.3) $k_{cat}^{app}{}_{(GSH)}$ @ 100 µM GSSCys

Comparison		P-value	
WT	→ D144A	<0.001	***
WT	→ D144K	<0.001	***
D144A	→ D144K	0.131	ns

C2.4) $k_{cat}^{app}{}_{(GSH)}$ @ 150 µM GSSCys

Comparison		P-value	
WT	→ D144A	0.027	*
WT	→ D144K	0.032	*
D144A	→ D144K	0.855	ns

C4.1) $K_m^{app}{}_{(GSH)}$ @ 25 µM GSSCys

Comparison		P-value	
WT	→ D144A	0.813	ns
WT	→ D144K	<0.001	***
D144A	→ D144K	<0.001	***

C4.2) $K_m^{app}{}_{(GSH)}$ @ 50 µM GSSCys

Comparison		P-value	
WT	→ D144A	0.056	ns
WT	→ D144K	<0.001	***
D144A	→ D144K	0.004	**

C4.3) $K_m^{app}{}_{(GSH)}$ @ 100 µM GSSCys

Comparison		P-value	
WT	→ D144A	0.366	ns
WT	→ D144K	<0.001	***
D144A	→ D144K	0.001	***

C4.4) $K_m^{app}{}_{(GSH)}$ @ 150 µM GSSCys

Comparison		P-value	
WT	→ D144A	0.325	ns
WT	→ D144K	0.020	*
D144A	→ D144K	0.009	**

D) GSSCys assay ScGrx7 E147X (Fig. 4, Supplementary Fig. 9 and Supplementary Table 5)

D1.1) $k_{cat}^{app}{}_{(GSSCys)}$ @ 50 µM GSH

Comparison		P-value	
WT	→ E147A	0.999	ns
WT	→ E147K	<0.001	***
E147A	→ E147K	<0.001	***

D1.2) $k_{cat}^{app}{}_{(GSSCys)}$ @ 100 µM GSH

Comparison		P-value	
WT	→ E147A	0.317	ns
WT	→ E147K	<0.001	***
E147A	→ E147K	<0.001	***

D1.3) $k_{cat}^{app}{}_{(GSSCys)}$ @ 200 µM GSH

Comparison		P-value	
WT	→ E147A	0.840	ns
WT	→ E147K	<0.001	***
E147A	→ E147K	<0.001	***

D1.4) $k_{cat}^{app}{}_{(GSSCys)}$ @ 1000 µM GSH

Comparison		P-value	
WT	→ E147A	0.427	ns
WT	→ E147K	<0.001	***
E147A	→ E147K	<0.001	***

D2.1) $k_{cat}^{app}{}_{(GSH)}$ @ 25 µM GSSCys

Comparison		P-value	
WT	→ E147A	0.054	ns
WT	→ E147K	0.006	**
E147A	→ E147K	0.058	ns

D2.2) $k_{cat}^{app}{}_{(GSH)}$ @ 50 µM GSSCys

Comparison		P-value	
WT	→ E147A	0.037	*
WT	→ E147K	0.004	**
E147A	→ E147K	0.042	*

D2.3) $k_{cat}^{app}{}_{(GSH)}$ @ 100 µM GSSCys

Comparison		P-value	
WT	→ E147A	0.003	**
WT	→ E147K	<0.001	***
E147A	→ E147K	0.002	**

D2.4) $k_{cat}^{app}{}_{(GSH)}$ @ 150 µM GSSCys

Comparison		P-value	
WT	→ E147A	0.003	**
WT	→ E147K	0.021	*
E147A	→ E147K	0.063	ns

D3.1) $K_m^{app}{}_{(GSSCys)}$ @ 50 µM GSH

Comparison		P-value	
WT	→ E147A	0.550	ns
WT	→ E147K	0.002	**
E147A	→ E147K	0.002	**

D3.2) $K_m^{app}{}_{(GSSCys)}$ @ 100 µM GSH

Comparison		P-value	
WT	→ E147A	0.641	ns
WT	→ E147K	0.001	***
E147A	→ E147K	0.001	***

D3.3) $K_m^{app}{}_{(GSSCys)}$ @ 200 µM GSH

Comparison		P-value	
WT	→ E147A	0.984	ns
WT	→ E147K	0.012	*
E147A	→ E147K	0.018	*

D3.4) $K_m^{app}{}_{(GSSCys)}$ @ 1000 µM GSH

Comparison		P-value	
WT	→ E147A	0.539	ns
WT	→ E147K	0.002	**
E147A	→ E147K	0.001	***

D4.1) $K_m^{app}{}_{(GSH)}$ @ 25 µM GSSCys

Comparison		P-value	
WT	→ E147A	0.125	ns
WT	→ E147K	0.025	*
E147A	→ E147K	0.005	**

D4.2) $K_m^{app}{}_{(GSH)}$ @ 50 µM GSSCys

Comparison		P-value	
WT	→ E147A	0.042	*
WT	→ E147K	0.025	*
E147A	→ E147K	0.003	**

D4.3) $K_m^{app}{}_{(GSH)}$ @ 100 µM GSSCys

Comparison		P-value	
WT	→ E147A	0.122	ns
WT	→ E147K	0.005	**
E147A	→ E147K	0.002	**

D4.4) $K_m^{app}{}_{(GSH)}$ @ 150 µM GSSCys

Comparison		P-value	
WT	→ E147A	0.008	**
WT	→ E147K	0.016	*
E147A	→ E147K	<0.001	***

E) GSSCys assay ScGrx7 R153X (Fig. 5, Supplementary Fig. 11 and Supplementary Table 6)

E1.1) $k_{cat}^{app}{}_{(GSSCys)}$ @ 50 µM GSH

Comparison		P-value	
WT	→ R153A	0.549	ns
WT	→ R153E	0.013	*
R153A	→ R153E	0.010	**

E1.2) $k_{cat}^{app}{}_{(GSSCys)}$ @ 100 µM GSH

Comparison		P-value	
WT	→ R153A	0.710	ns
WT	→ R153E	0.004	**
R153A	→ R153E	0.004	**

E1.3) $k_{cat}^{app}{}_{(GSSCys)}$ @ 200 µM GSH

Comparison		P-value	
WT	→ R153A	0.033	*
WT	→ R153E	<0.001	***
R153A	→ R153E	0.003	**

E1.4) $k_{cat}^{app}{}_{(GSSCys)}$ @ 1000 µM GSH

Comparison		P-value	
WT	→ R153A	0.125	ns
WT	→ R153E	0.005	**
R153A	→ R153E	0.021	*

E2.1) $k_{cat}^{app}{}_{(GSH)}$ @ 25 µM GSSCys

Comparison		P-value	
WT	→ R153A	<0.001	***
WT	→ R153E	<0.001	***
R153A	→ R153E	<0.001	***

E2.2) $k_{cat}^{app}{}_{(GSH)}$ @ 50 µM GSSCys

Comparison		P-value	
WT	→ R153A	<0.001	***
WT	→ R153E	<0.001	***
R153A	→ R153E	<0.001	***

E2.3) $k_{cat}^{app}{}_{(GSH)}$ @ 100 µM GSSCys

Comparison		P-value	
WT	→ R153A	<0.001	***
WT	→ R153E	<0.001	***
R153A	→ R153E	<0.001	***

E2.4) $k_{cat}^{app}{}_{(GSH)}$ @ 150 µM GSSCys

Comparison		P-value	
WT	→ R153A	<0.001	***
WT	→ R153E	<0.001	***
R153A	→ R153E	<0.001	***

E3.1) $K_{m}^{app}{}_{(GSSCys)}$ @ 50 µM GSH

Comparison		P-value	
WT	→ R153A	0.043	*
WT	→ R153E	0.036	*
R153A	→ R153E	0.857	ns

E3.2) $K_{m}^{app}{}_{(GSSCys)}$ @ 100 µM GSH

Comparison		P-value	
WT	→ R153A	0.039	*
WT	→ R153E	0.043	*
R153A	→ R153E	0.713	ns

E3.3) $K_{m}^{app}{}_{(GSSCys)}$ @ 200 µM GSH

Comparison		P-value	
WT	→ R153A	0.015	*
WT	→ R153E	0.021	*
R153A	→ R153E	0.950	ns

E3.4) $K_{m}^{app}{}_{(GSSCys)}$ @ 1000 µM GSH

Comparison		P-value	
WT	→ R153A	0.008	**
WT	→ R153E	0.011	*
R153A	→ R153E	0.970	ns

E4.1) $K_{m}^{app}{}_{(GSH)}$ @ 25 µM GSSCys

Comparison		P-value	
WT	→ R153A	<0.001	***
WT	→ R153E	<0.001	***
R153A	→ R153E	0.255	ns

E4.2) $K_{m}^{app}{}_{(GSH)}$ @ 50 µM GSSCys

Comparison		P-value	
WT	→ R153A	<0.001	***
WT	→ R153E	<0.001	***
R153A	→ R153E	0.760	ns

E4.3) $K_{m}^{app}{}_{(GSH)}$ @ 100 µM GSSCys

Comparison		P-value	
WT	→ R153A	<0.001	***
WT	→ R153E	<0.001	***
R153A	→ R153E	0.454	ns

E4.4) $K_{m}^{app}{}_{(GSH)}$ @ 150 µM GSSCys

Comparison		P-value	
WT	→ R153A	0.005	**
WT	→ R153E	0.006	**
R153A	→ R153E	0.611	ns

Supplementary Table 12. Statistical analysis of AUC (OxD*sec) values from the indicated measurements of ScGrx7. *P*-values from one way ANOVA analysis followed by a Holm-Sidak test were calculated in SigmaPlot 13 (P > 0.05 : ns; P ≤ 0.05 : *; P ≤ 0.01 : **, P ≤ 0.001 : ***).

A) roGFP2 assay ScGrx7 C108S and unfused roGFP2 (Fig. 7b)

A1) AUC (OxD*sec) @ 20 µM H_2O_2

Comparison		P-value	
WT	→ C108S	0.381	ns
WT	→ roGFP2	0.305	ns
C108S	→ roGFP2	0.888	ns

A2) AUC (OxD*sec) @ 50 µM H_2O_2

Comparison		P-value	
WT	→ C108S	0.235	ns
WT	→ roGFP2	0.177	ns
C108S	→ roGFP2	0.221	ns

A3) AUC (OxD*sec) @ 100 µM H_2O_2

Comparison		P-value	
WT	→ C108S	0.070	ns
WT	→ roGFP2	0.071	ns
C108S	→ roGFP2	0.942	ns

A4) AUC (OxD*sec) @ 200 µM H_2O_2

Comparison		P-value	
WT	→ C108S	0.002	**
WT	→ roGFP2	0.002	**
C108S	→ roGFP2	0.727	ns

A5) AUC (OxD*sec) @ 500 µM H_2O_2

Comparison		P-value	
WT	→ C108S	<0.001	***
WT	→ roGFP2	<0.001	***
C108S	→ roGFP2	0.323	ns

A6) AUC (OxD*sec) @ 1000 µM H_2O_2

Comparison		P-value	
WT	→ C108S	<0.001	***
WT	→ roGFP2	<0.001	***
C108S	→ roGFP2	0.595	ns

B) roGFP2 assay ScGrx7 K105X (Fig. 7c)

B1) AUC (OxD*sec) @ 20 µM H_2O_2

Comparison		P-value	
WT	→ K105R	0.273	ns
WT	→ K105A	0.210	ns
WT	→ K105Y	0.647	ns
WT	→ K105E	0.985	ns
K105R	→ K105A	0.843	ns
K105R	→ K105Y	0.134	ns
K105R	→ K105E	0.400	ns
K105A	→ K105Y	0.092	ns
K105A	→ K105E	0.629	ns
K105Y	→ K105E	0.400	ns

B2) AUC (OxD*sec) @ 50 µM H_2O_2

Comparison		P-value	
WT	→ K105R	0.227	ns
WT	→ K105A	0.184	ns
WT	→ K105Y	0.225	ns
WT	→ K105E	0.214	ns
K105R	→ K105A	0.330	ns
K105R	→ K105Y	0.607	ns
K105R	→ K105E	0.629	ns
K105A	→ K105Y	0.198	ns
K105A	→ K105E	0.400	ns
K105Y	→ K105E	0.629	ns

B3) AUC (OxD*sec) @ 100 µM H_2O_2

Comparison		P-value	
WT	→ K105R	0.069	ns
WT	→ K105A	0.102	ns
WT	→ K105Y	0.213	ns
WT	→ K105E	0.048	*
K105R	→ K105A	0.202	ns
K105R	→ K105Y	0.011	*
K105R	→ K105E	0.393	ns
K105A	→ K105Y	0.074	ns
K105A	→ K105E	0.651	ns
K105Y	→ K105E	0.031	*

B4) AUC (OxD*sec) @ 200 µM H_2O_2

Comparison		P-value	
WT	→ K105R	0.003	**
WT	→ K105A	0.050	*
WT	→ K105Y	0.013	*
WT	→ K105E	<0.001	***
K105R	→ K105A	0.090	ns
K105R	→ K105Y	0.070	ns
K105R	→ K105E	0.043	*
K105A	→ K105Y	0.257	ns
K105A	→ K105E	0.003	**
K105Y	→ K105E	0.007	**

B5) AUC (OxD*sec) @ 500 µM H_2O_2

Comparison		P-value	
WT	→ K105R	0.004	**
WT	→ K105A	0.002	**
WT	→ K105Y	0.024	*
WT	→ K105E	<0.001	***
K105R	→ K105A	0.482	ns
K105R	→ K105Y	0.013	*
K105R	→ K105E	0.002	**
K105A	→ K105Y	<0.001	***
K105A	→ K105E	<0.001	***
K105Y	→ K105E	<0.001	***

B6) AUC (OxD*sec) @ 1000 µM H_2O_2

Comparison		P-value	
WT	→ K105R	0.002	**
WT	→ K105A	0.003	**
WT	→ K105Y	0.004	**
WT	→ K105E	<0.001	***
K105R	→ K105A	0.874	ns
K105R	→ K105Y	0.450	ns
K105R	→ K105E	<0.001	***
K105A	→ K105Y	0.665	ns
K105A	→ K105E	<0.001	***
K105Y	→ K105E	<0.001	***

C) roGFP2 assay ScGrx7 Y110X (Fig. 7d)

C1) AUC (OxD*sec) @ 20 µM H_2O_2

Comparison		P-value	
WT	→ Y110F	0.438	ns
WT	→ Y110H	0.150	ns
WT	→ Y110A	0.299	ns
Y110F	→ Y110H	0.705	ns
Y110F	→ Y110A	0.245	ns
Y110H	→ Y110A	0.200	ns

C2) AUC (OxD*sec) @ 50 µM H_2O_2

Comparison		P-value	
WT	→ Y110F	0.515	ns
WT	→ Y110H	0.423	ns
WT	→ Y110A	0.256	ns
Y110F	→ Y110H	0.205	ns
Y110F	→ Y110A	0.140	ns
Y110H	→ Y110A	0.400	ns

C3) AUC (OxD*sec) @ 100 µM H_2O_2

Comparison		P-value	
WT	→ Y110F	0.466	ns
WT	→ Y110H	0.452	ns
WT	→ Y110A	0.178	ns
Y110F	→ Y110H	0.198	ns
Y110F	→ Y110A	0.080	ns
Y110H	→ Y110A	0.483	ns

C4) AUC (OxD*sec) @ 200 µM H_2O_2

Comparison		P-value	
WT	→ Y110F	0.669	ns
WT	→ Y110H	0.043	*
WT	→ Y110A	0.003	**
Y110F	→ Y110H	0.003	**
Y110F	→ Y110A	<0.001	***
Y110H	→ Y110A	0.003	**

C5) AUC (OxD*sec) @ 500 µM H_2O_2

Comparison		P-value	
WT	→ Y110F	0.857	ns
WT	→ Y110H	0.064	ns
WT	→ Y110A	0.006	**
Y110F	→ Y110H	0.105	ns
Y110F	→ Y110A	0.028	*
Y110H	→ Y110A	0.100	ns

C6) AUC (OxD*sec) @ 1000 µM H_2O_2

Comparison		P-value	
WT	→ Y110F	0.035	*
WT	→ Y110H	0.303	ns
WT	→ Y110A	0.003	**
Y110F	→ Y110H	0.027	*
Y110F	→ Y110A	0.003	**
Y110H	→ Y110A	0.012	*

D) roGFP2 assay ScGrx7 D144X (Fig. 7e)

D1) AUC (OxD*sec) @ 20 µM H_2O_2

Comparison		P-value	
WT	→ D144A	0.411	ns
WT	→ D144K	0.578	ns
D144K	→ D144A	0.285	ns

D2) AUC (OxD*sec) @ 50 µM H_2O_2

Comparison		P-value	
WT	→ D144A	0.626	ns
WT	→ D144K	0.840	ns
D144K	→ D144A	0.441	ns

D3) AUC (OxD*sec) @ 100 µM H_2O_2

Comparison		P-value	
WT	→ D144A	0.689	ns
WT	→ D144K	0.965	ns
D144K	→ D144A	0.757	ns

D4) AUC (OxD*sec) @ 200 µM H_2O_2

Comparison		P-value	
WT	→ D144A	0.086	ns
WT	→ D144K	0.026	*
D144K	→ D144A	0.969	ns

D5) AUC (OxD*sec) @ 500 µM H_2O_2

Comparison		P-value	
WT	→ D144A	0.583	ns
WT	→ D144K	0.800	ns
D144K	→ D144A	0.780	ns

D6) AUC (OxD*sec) @ 1000 µM H_2O_2

Comparison		P-value	
WT	→ D144A	0.106	ns
WT	→ D144K	0.748	ns
D144K	→ D144A	0.302	ns

E) roGFP2 assay ScGrx7 E147X (Fig. 7e)

E1) AUC (OxD*sec) @ 20 µM H_2O_2

Comparison		P-value	
WT	→ E147A	0.624	ns
WT	→ E147K	0.686	ns
E147A	→ E147K	0.807	ns

E2) AUC (OxD*sec) @ 50 µM H_2O_2

Comparison		P-value	
WT	→ E147A	0.443	ns
WT	→ E147K	0.523	ns
E147A	→ E147K	0.748	ns

E3) AUC (OxD*sec) @ 100 µM H_2O_2

Comparison		P-value	
WT	→ E147A	0.392	ns
WT	→ E147K	0.577	ns
E147A	→ E147K	0.154	ns

E4) AUC (OxD*sec) @ 200 µM H_2O_2

Comparison		P-value	
WT	→ E147A	0.517	ns
WT	→ E147K	0.229	ns
E147A	→ E147K	0.997	ns

E5) AUC (OxD*sec) @ 500 µM H_2O_2

Comparison		P-value	
WT	→ E147A	0.069	ns
WT	→ E147K	0.073	ns
E147A	→ E147K	0.491	ns

E6) AUC (OxD*sec) @ 1000 µM H_2O_2

Comparison		P-value	
WT	→ E147A	0.050	*
WT	→ E147K	0.080	ns
E147A	→ E147K	0.886	ns

F) roGFP2 assay ScGrx7 R153X and E170A (Fig. 7f)

F1) AUC (OxD*sec) @ 20 µM H_2O_2

Comparison		P-value	
WT	→ R153A	0.629	ns
WT	→ R153E	0.178	ns
R153A	→ R153E	0.545	ns
WT	→ E170A	0.119	ns

F2) AUC (OxD*sec) @ 50 µM H_2O_2

Comparison		P-value	
WT	→ R153A	0.316	ns
WT	→ R153E	0.207	ns
R153A	→ R153E	0.164	ns
WT	→ E170A	0.182	ns

F3) AUC (OxD*sec) @ 100 µM H_2O_2

Comparison		P-value	
WT	→ R153A	0.200	ns
WT	→ R153E	0.120	ns
R153A	→ R153E	0.242	ns
WT	→ E170A	0.108	ns

F4) AUC (OxD*sec) @ 200 µM H_2O_2

Comparison		P-value	
WT	→ R153A	0.015	*
WT	→ R153E	0.009	**
R153A	→ R153E	0.140	ns
WT	→ E170A	0.008	**

F5) AUC (OxD*sec) @ 500 µM H_2O_2

Comparison		P-value	
WT	→ R153A	0.058	ns
WT	→ R153E	0.113	ns
R153A	→ R153E	0.574	ns
WT	→ E170A	0.010	**

F6) AUC (OxD*sec) @ 1000 µM H_2O_2

Comparison		P-value	
WT	→ R153A	0.287	ns
WT	→ R153E	0.221	ns
R153A	→ R153E	0.525	ns
WT	→ E170A	0.006	**

G) roGFP2 assay ScGrx7 interconversion mutants with DTT pretreatment (Fig. 7g)

G1) AUC (OxD*sec) @ 20 µM H_2O_2

Comparison		P-value	
WT	→ roGFP2	0.083	ns
WT	→ C108S	0.044	*
WT	→ WP	0.229	ns
WT	→ loop	0.198	ns
WT	→ WP+loop	0.037	*
WP	→ roGFP2	0.982	ns
WP	→ C108S	0.985	ns
WP	→ loop	0.887	ns
WP	→ WP+loop	0.599	ns
loop	→ roGFP2	0.862	ns
loop	→ C108S	0.796	ns
loop	→ WP+loop	0.412	ns
WP+loop	→ roGFP2	0.196	ns
WP+loop	→ C108S	0.181	ns
roGFP2	→ C108S	0.863	ns

G2) AUC (OxD*sec) @ 50 µM H_2O_2

Comparison		P-value	
WT	→ roGFP2	0.106	ns
WT	→ C108S	0.043	*
WT	→ WP	0.318	ns
WT	→ loop	0.108	ns
WT	→ WP+oop	0.089	ns
WP	→ roGFP2	0.475	ns
WP	→ C108S	0.273	ns
WP	→ loop	0.602	ns
WP	→ WP+loop	0.383	ns
loop	→ roGFP2	0.793	ns
loop	→ C108S	0.472	ns
loop	→ WP+loop	0.569	ns
WP+loop	→ roGFP2	0.580	ns
WP+loop	→ C108S	0.981	ns
roGFP2	→ C108S	0.318	ns

G3) AUC (OxD*sec) @ 100 µM H_2O_2

Comparison		P-value	
WT	→ roGFP2	0.002	*
WT	→ C108	<0.001	***
WT	→ WP	0.002	**
WT	→ loop	0.001	**
WT	→ WP+loop	0.003	**
WP	→ roGFP2	0.762	ns
WP	→ C108S	0.595	ns
WP	→ loop	0.739	ns
WP	→ WP+loop	0.830	ns
loop	→ roGFP2	0.880	ns
loop	→ C108S	0.998	ns
loop	→ WP+loop	0.613	ns
WP+Loop	→ roGFP2	0.643	ns
WP+Loop	→ C108S	0.500	ns
roGFP2	→ C108S	0.610	ns

G4) AUC (OxD*sec) @ 200 µM H_2O_2

Comparison		P-value	
WT	→ roGFP2	0.007	*
WT	→ C108S	0.002	**
WT	→ WP	0.009	**
WT	→ loop	0.002	**

WT	→ WP+loop	0.007	**
WP	→ roGFP2	0.994	ns
WP	→ C108S	0.844	ns
WP	→ loop	0.592	ns
WP	→ WP+loop	0.691	ns
loop	→ roGFP2	0.512	ns
loop	→ C108S	0.359	ns
loop	→ WP+loop	0.881	ns
WP+loop	→ roGFP2	0.575	ns
WP+loop	→ C108S	0.420	ns
roGFP2	→ C108S	0.717	ns

G5) AUC (OxD*sec) @ 500 µM H_2O_2

Comparison		P-value	
WT	→ roGFP2	0.001	**
WT	→ C108	<0.001	***
WT	→ WP	0.004	**
WT	→ loop	<0.001	***
WT	→ WP+loop	0.036	*
WP	→ roGFP2	0.218	ns
WP	→ C108S	0.221	ns
WP	→ loop	0.125	ns
WP	→ WP+loop	0.070	ns
loop	→ roGFP2	0.502	ns
loop	→ C108S	0.319	ns
loop	→ WP+loop	0.452	ns
WP+loop	→ roGFP2	0.060	ns
WP+loop	→ C108S	0.041	*
roGFP2	→ C108S	0.631	ns

G6) AUC (OxD*sec) @ 1000 µM H_2O_2

Comparison		P-value	
WT	→ roGFP2	<0.001	***
WT	→ C108	<0.001	***
WT	→ WP	0.003	**
WT	→ loop	<0.001	***
WT	→ WP+loop	<0.001	***
WP	→ roGFP2	0.418	ns
WP	→ C108S	0.435	ns
WP	→ loop	0.074	ns
WP	→ WP+loop	0.106	ns
loop	→ roGFP2	0.081	ns
loop	→ C108S	0.013	*
loop	→ WP+loop	0.733	ns
WP+loop	→ roGFP2	0.076	ns
WP+loop	→ C108S	0.011	*
roGFP2	→ C108S	0.636	ns

H) roGFP2 assay ScGrx5 interconversion mutants (Fig. 7h)

H1) AUC (OxD*sec) @ 20 µM H_2O_2

Comparison		P-value	
WT	→ RR	0.990	ns
WT	→ loop	0.725	ns
WT	→ RR+loop	0.223	ns
RR	→ loop	0.700	ns
RR	→ RR+loop	0.057	ns
loop	→ RR+loop	0.629	ns

H2) AUC (OxD*sec) @ 50 µM H_2O_2

Comparison		P-value	
WT	→ RR	0.793	ns
WT	→ loop	0.839	ns
WT	→ RR+loop	0.649	ns
RR	→ loop	0.876	ns
RR	→ RR+loop	0.262	ns
loop	→ RR+loop	0.857	ns

H3) AUC (OxD*sec) @ 100 µM H_2O_2

Comparison		P-value	
WT	→ RR	0.684	ns
WT	→ loop	0.614	ns
WT	→ RR+loop	0.794	ns
RR	→ loop	0.690	ns
RR	→ RR+loop	0.445	ns
loop	→ RR+loop	1.000	ns

H4) AUC (OxD*sec) @ 200 µM H_2O_2

Comparison		P-value	
WT	→ RR	0.414	ns
WT	→ loop	0.700	ns
WT	→ RR+loop	0.606	ns
RR	→ loop	0.530	ns
RR	→ RR+loop	0.986	ns
loop	→ RR+loop	0.493	ns

H5) AUC (OxD*sec) @ 500 µM H_2O_2

Comparison		P-value	
WT	→ RR	0.100	ns
WT	→ loop	0.883	ns
WT	→ RR+loop	0.844	ns
RR	→ loop	0.068	ns
RR	→ RR+loop	0.046	*
loop	→ RR+loop	0.680	ns

H6) AUC (OxD*sec) @ 1000 µM H_2O_2

Comparison		P-value	
WT	→ RR	0.700	ns
WT	→ loop	0.188	ns
WT	→ RR+loop	0.315	ns
RR	→ loop	0.047	*
RR	→ RR+loop	0.036	*
loop	→ RR+loop	0.382	ns

I) roGFP2 assay ScGrx5 interconversion mutants with DTT pretreatment (Fig. 7i)

I1) AUC (OxD*sec) @ 20 µM H_2O_2

Comparison		P-value	
WT	→roGFP2	0.084	ns
WT	→loop	0.362	ns
WT	→loop+G68P	0.044	*
WT	→RR+loop	0.070	ns
WT	→RR+loop+G68P	0.090	ns
WT	→loop+G68P+R97Q	0.035	**
loop	→roGFP2	0.872	ns
loop	→loop+G68P	0.442	ns
loop	→RR+loop	0.776	ns
loop	→RR+loop+G68P	0.676	ns
loop	→loop+G68P+R97Q	0.416	ns
loop+G68P	→roGFP2	0.217	ns
loop+G68P	→RR+loop	0.308	ns
loop+G68P	→RR+loop+G68P	0.564	ns
loop+G68P	→loop+G68P+R97Q	0.963	ns
RR+loop	→roGFP2	0.700	ns
RR+loop	→RR+loop+G68P	0.754	ns
RR+loop	→loop+G68P+R97Q	0.244	ns
RR+loop+G68P	→roGFP2	0.586	ns
RR+loop+G68P	→loop+G68P+R97Q	0.514	ns
loop+G68P+R97Q	→roGFP2	0.162	ns

I2) AUC (OxD*sec) @ 50 µM H_2O_2

Comparison		P-value	
WT	→roGFP2	0.092	ns
WT	→loop	0.524	ns
WT	→loop+G68P	0.157	ns
WT	→RR+loop	0.050	*
WT	→RR+loop+G68P	0.192	ns
WT	→loop+G68P+R97Q	0.044	**
loop	→roGFP2	0.147	ns
loop	→loop+G68P	0.230	ns
loop	→RR+loop	0.597	ns
loop	→RR+loop+G68P	0.327	ns
loop	→loop+G68P+R97Q	0.065	ns
loop+G68P	→roGFP2	0.473	ns
loop+G68P	→RR+loop	0.597	ns
loop+G68P	→RR+loop+G68P	0.657	ns
loop+G68P	→loop+G68P+R97Q	0.764	ns
RR+loop	→roGFP2	0.144	ns
RR+loop	→RR+loop+G68P	0.295	ns
RR+loop	→loop+G68P+R97Q	0.744	ns
RR+loop+G68P	→roGFP2	0.796	ns
RR+loop+G68P	→loop+G68P+R97Q	0.370	ns
loop+G68P+R97Q	→roGFP2	0.142	ns

I3) AUC (OxD*sec) @ 100 µM H_2O_2

Comparison		P-value	
WT	→roGFP2	0.575	ns
WT	→loop	0.889	ns
WT	→loop+G68P	0.107	ns
WT	→RR+loop	0.103	ns
WT	→RR+loop+G68P	0.301	ns
WT	→loop+G68P+R97Q	0.234	ns
loop	→roGFP2	0.544	ns
loop	→loop+G68P	0.123	ns
loop	→RR+loop	0.135	ns
loop	→RR+loop+G68P	0.319	ns
loop	→loop+G68P+R97Q	0.226	ns
loop+G68P	→roGFP2	0.088	ns
loop+G68P	→RR+loop	0.597	ns
loop+G68P	→RR+loop+G68P	0.226	ns
loop+G68P	→loop+G68P+R97Q	0.470	ns
RR+loop	→roGFP2	0.030	*
RR+loop	→RR+loop+G68P	0.226	ns
RR+loop	→loop+G68P+R97Q	0.399	ns
RR+loop+G68P	→roGFP2	0.270	ns
RR+loop+G68P	→loop+G68P+R97Q	0.316	ns
loop+G68P+R97Q	→roGFP2	0.264	ns

I4) AUC (OxD*sec) @ 200 µM H_2O_2

Comparison		P-value	
WT	→roGFP2	0.228	ns
WT	→loop	0.271	ns
WT	→loop+G68P	0.019	*
WT	→RR+loop	0.087	ns
WT	→RR+loop+G68P	0.069	ns
WT	→loop+G68P+R97Q	0.027	*
loop	→roGFP2	0.542	ns
loop	→loop+G68P	0.205	ns
loop	→RR+loop	0.282	ns
loop	→RR+loop+G68P	0.200	ns
loop	→loop+G68P+R97Q	0.049	*
loop+G68P	→roGFP2	0.027	*
loop+G68P	→RR+loop	0.799	ns
loop+G68P	→RR+loop+G68P	0.547	ns
loop+G68P	→loop+G68P+R97Q	0.089	ns
RR+loop	→roGFP2	0.143	ns
RR+loop	→RR+loop+G68P	0.760	ns
RR+loop	→loop+G68P+R97Q	0.135	ns
RR+loop+G68P	→roGFP2	0.101	ns
RR+loop+G68P	→loop+G68P+R97Q	0.189	ns
loop+G68P+R97Q	→roGFP2	0.033	*

I5) AUC (OxD*sec) @ 500 µM H_2O_2

Comparison		P-value	
WT	→roGFP2	0.263	ns
WT	→loop	0.429	ns
WT	→loop+G68P	0.068	ns
WT	→RR+loop	0.004	**
WT	→RR+loop+G68P	0.088	ns
WT	→loop+G68P+R97Q	0.005	**
loop	→roGFP2	0.203	ns
loop	→loop+G68P	0.406	ns
loop	→RR+loop	0.029	*
loop	→RR+loop+G68P	0.165	ns
loop	→loop+G68P+R97Q	0.012	*
loop+G68P	→roGFP2	0.023	*
loop+G68P	→RR+loop	0.045	*
loop+G68P	→RR+loop+G68P	0.269	ns
loop+G68P	→loop+G68P+R97Q	0.015	*
RR+loop	→roGFP2	0.002	ns
RR+loop	→RR+loop+G68P	0.979	ns
RR+loop	→loop+G68P+R97Q	0.079	ns
RR+loop+G68P	→roGFP2	0.065	ns
RR+loop+G68P	→loop+G68P+R97Q	0.191	ns
loop+G68P+R97Q	→roGFP2	0.004	**

I6) AUC (OxD*sec) @ 1000 µM H_2O_2

Comparison		P-value	
WT	→roGFP2	0.010	**
WT	→loop	0.723	ns
WT	→loop+G68P	0.013	*
WT	→RR+loop	0.002	**
WT	→RR+loop+G68P	<0.001	***
WT	→loop+G68P+R97Q	0.032	*
loop	→roGFP2	0.025	*
loop	→loop+G68P	0.014	*
loop	→RR+loop	0.002	**
loop	→RR+loop+G68P	0.001	**
loop	→loop+G68P+R97Q	0.029	*
loop+G68P	→roGFP2	<0.001	***
loop+G68P	→RR+loop	0.004	**
loop+G68P	→RR+loop+G68P	0.002	**
loop+G68P	→loop+G68P+R97Q	0.099	ns
RR+loop	→roGFP2	<0.001	***
RR+loop	→RR+loop+G68P	0.021	*
RR+loop	→loop+G68P+R97Q	0.399	ns
RR+loop+G68P	→roGFP2	<0.001	***
RR+loop+G68P	→loop+G68P+R97Q	0.682	ns
loop+G68P+R97Q	→roGFP2	0.010	**

J) roGFP2 assay ScGrx7 interconversion mutants (Supplementary Fig. 17)

J1) AUC (OxD*sec) @ 20 µM H_2O_2

Comparison		P-value	
WT	→ WP	0.803	ns
WT	→ loop	0.396	ns
WT	→ WP+loop	0.401	ns
WP	→ loop	0.448	ns
WP	→ WP+loop	0.250	ns
loop	→ WP+loop	0.828	ns

J2) AUC (OxD*sec) @ 50 µM H_2O_2

Comparison		P-value	
WT	→ WP	0.230	ns
WT	→ loop	0.189	ns
WT	→ WP+loop	0.172	ns
WP	→ loop	0.555	ns
WP	→ WP+loop	0.427	ns
loop	→ WP+loop	0.700	ns

J3) AUC (OxD*sec) @ 100 µM H_2O_2

Comparison		P-value	
WT	→ WP	<0.001	***
WT	→ loop	<0.001	***
WT	→ WP+loop	0.100	ns
WP	→ loop	0.939	ns
WP	→ WP+loop	0.808	ns
loop	→ WP+loop	0.678	ns

J4) AUC (OxD*sec) @ 200 µM H_2O_2

Comparison		P-value	
WT	→ WP	0.005	**
WT	→ loop	0.035	*
WT	→ WP+loop	0.038	*
WP	→ loop	0.873	ns
WP	→ WP+loop	0.664	ns
loop	→ WP+loop	0.716	ns

J5) AUC (OxD*sec) @ 500 µM H_2O_2

Comparison		P-value	
WT	→ WP	0.036	*
WT	→ loop	0.008	**
WT	→ WP+loop	0.008	**
WP	→ loop	0.034	*
WP	→ WP+loop	0.117	ns
loop	→ WP+loop	0.794	ns

J6) AUC (OxD*sec) @ 1000 µM H_2O_2

Comparison		P-value	
WT	→ WP	<0.001	***
WT	→ loop	0.002	**
WT	→ WP+loop	0.003	**
WP	→ loop	0.051	ns
WP	→ WP+loop	0.102	ns
loop	→ WP+loop	0.828	ns

Supplementary Table 13. Statistical analysis of the fraction of bound states from the MD simulations of ScGrx7.[a]

**Comparison**		***p*-value**	
WT	K105R	0.155	ns
WT	K105E	0.043	*
WT	E147K	0.153	ns
K105R	K105E	0.052	ns
K105R	E147K	0.806	ns
K105E	E147K	0.065	ns

[a] See also Fig. 8a. *P*-values are derived from a two-tailed t-test assuming unequal variances. ($p < 0.05$: *; $p \geq 0.05$: ns)

Supplementary Table 14. Correlation analysis of the S-S-distance vs. S-S-S-angle from the MD simulations of ScGrx7.[a]

**ScGrx7**	R^2	***p*-value**
WT	0.01	0.851
K105R	0.42	< 0.001
K105E	0.07	0.293
E147K	0.08	0.001

[a] See also Fig. 8e.

Supplementary Table 15. ScGrx7 residues forming contacts with GS^{-} during MD simulations.

ScGrx7 Residue[a]		Contact fraction[b]			
		WT	K105R	K105E	E147K
K	105	0.09	0.18	0.04	0.08
T	106	0.26	0.26	0.21	0.28
G	107	0.27	0.31	0.20	0.26
γE	SSG	0.23	0.17	0.26	0.31
C	SSG	0.11	0.20	0.03	0.12
G	SSG	0.19	0.22	0.10	0.29
P	109	0.25	0.29	0.18	0.20
Y	110	0.17	0.14	0.10	0.11
K	112	0.44	0.42	0.38	0.40
K	113	0.40	0.39	0.34	0.39
K	115	0.33	0.42	0.21	0.35
A	116	0.27	0.22	0.24	0.25
T	119	0.15	0.14	0.11	0.13
N	120	0.22	0.19	0.19	0.19
S	125	0.16	0.15	0.13	0.14
V	130	0.22	0.31	0.10	0.24
E	132	0.26	0.35	0.17	0.29
R	135	0.42	0.47	0.30	0.44
K	140	0.16	0.15	0.11	0.18
E	147	0.09	0.05	0.06	0.47
G	151	0.13	0.11	0.20	0.43
R	152	0.17	0.14	0.33	0.46
R	153	0.15	0.11	0.10	0.38
T	154	0.29	0.17	0.28	0.67
T	163	0.12	0.13	0.21	0.19
S	164	0.07	0.06	0.20	0.21
G	166	0.05	0.07	0.12	0.25
T	169	0.17	0.12	0.21	0.19
E	170	0.14	0.10	0.24	0.23
K	176	0.17	0.19	0.14	0.15
K	185	0.15	0.16	0.14	0.16
K	186	0.18	0.26	0.18	0.26
D	189	0.12	0.19	0.13	0.15
T	193	0.18	0.17	0.16	0.17
K	195	0.21	0.22	0.24	0.24

[a] Residue number of the contacting residue. SSG γE, C, and G represent the γ-glutamyl, cysteinyl, and glycyl moiety of the bound disulfide glutathione, respectively. Only residues that have a fraction of 0.15 or higher in at least one of the ScGrx7 systems are shown.

[b] Fraction of the simulation time in which a contact between any GS^{-} molecule and the residue was present.
