# Supplementary material for: Quantitative assessment of the determinant structural differences between redox-active and inactive glutaredoxins
Source: Nat Commun. 2020 Apr 7;11:1725. doi: 10.1038/s41467-020-15441-3 (PMC7138851; doi:10.1038/s41467-020-15441-3)
Supplement: Supplementary file 3 — Reporting Summary [file 41467_2020_15441_MOESM3_ESM.pdf]

nature research

Corresponding author(s): Marcel Deponte, Bruce Morgan, Holger Gohlke

Last updated by author(s): Jan 29, 2020

Reporting Summary

Nature Research wishes to improve the reproducibility of the work that we publish. This form provides structure for consistency and transparency in reporting. For further information on Nature Research policies, see Authors & Referees and the Editorial Policy Checklist .

Statistics

For all statistical analyses, confirm that the following items are present in the figure legend, table legend, main text, or Methods section.

n/a	Confirmed	
☐	☒	The exact sample size (*n*) for each experimental group/condition, given as a discrete number and unit of measurement
☐	☒	A statement on whether measurements were taken from distinct samples or whether the same sample was measured repeatedly
☐	☒	The statistical test(s) used AND whether they are one- or two-sided<br>*Only common tests should be described solely by name; describe more complex techniques in the Methods section.*
☐	☒	A description of all covariates tested
☐	☒	A description of any assumptions or corrections, such as tests of normality and adjustment for multiple comparisons
☐	☒	A full description of the statistical parameters including central tendency (e.g. means) or other basic estimates (e.g. regression coefficient) AND variation (e.g. standard deviation) or associated estimates of uncertainty (e.g. confidence intervals)
☐	☒	For null hypothesis testing, the test statistic (e.g. *F*, *t*, *r*) with confidence intervals, effect sizes, degrees of freedom and *P* value noted<br>*Give P values as exact values whenever suitable.*
☐	☒	For Bayesian analysis, information on the choice of priors and Markov chain Monte Carlo settings
☐	☒	For hierarchical and complex designs, identification of the appropriate level for tests and full reporting of outcomes
☐	☒	Estimates of effect sizes (e.g. Cohen's *d*, Pearson's *r*), indicating how they were calculated

Our web collection on statistics for biologists contains articles on many of the points above.

Software and code

Policy information about availability of computer code

Data collection	See detailed materials and methods section
Data analysis	See detailed materials and methods section and figure legends

For manuscripts utilizing custom algorithms or software that are central to the research but not yet described in published literature, software must be made available to editors/reviewers. We strongly encourage code deposition in a community repository (e.g. GitHub). See the Nature Research guidelines for submitting code & software for further information.

Data

Policy information about availability of data

All manuscripts must include a data availability statement. This statement should provide the following information, where applicable:

- Accession codes, unique identifiers, or web links for publicly available datasets
- A list of figures that have associated raw data
- A description of any restrictions on data availability

All relevant data are included in the manuscript or its supplementary information and are available from the authors upon request.

Field-specific reporting

Please select the one below that is the best fit for your research. If you are not sure, read the appropriate sections before making your selection.

☒ Life sciences ☐ Behavioural & social sciences ☐ Ecological, evolutionary & environmental sciences

For a reference copy of the document with all sections, see nature.com/documents/nr-reporting-summary-flat.pdf

Life sciences study design

All studies must disclose on these points even when the disclosure is negative.

Sample size	All steady-state kinetic data were obtained from at least three independent expression/protein purification experiments. RoGFP2 data was obtained from at least three independent biological replicates. MD simulation data is based on four replicates.
Data exclusions	All data points were averaged from three independent protein purification experiments and subsequently plotted and fitted in SigmaPlot 13 according to Michaelis-Menten, Lineweaver-Burk, Eadie-Hofstee and Hanes theory (the latter two plots are not shown). Calculated kcatapp and Kmapp values from the four different plots usually varied by less than 10%. Data points in brackets were omitted from the regression analysis when the kcatapp or Kmapp values from all four plots varied by more than 10% and converged after removal of the outlier.
Replication	All findings could be replicated. Data comparability between students was confirmed with the help of defined controls.
Randomization	Samples were not randomized. Wild-type ScGrx7 was studied in parallel and served as a control.
Blinding	Blinding was not necessary because of the inclusion of positive and negative controls and was also not possible because of a restricted number of students conducting each of the experiments.

Reporting for specific materials, systems and methods

We require information from authors about some types of materials, experimental systems and methods used in many studies. Here, indicate whether each material, system or method listed is relevant to your study. If you are not sure if a list item applies to your research, read the appropriate section before selecting a response.

Materials & experimental systems

n/a	Involved in the study
☒	☐ Antibodies
☐	☒ Eukaryotic cell lines
☒	☐ Palaeontology
☒	☐ Animals and other organisms
☒	☐ Human research participants
☒	☐ Clinical data

Methods

n/a	Involved in the study
☒	☐ ChIP-seq
☒	☐ Flow cytometry
☒	☐ MRI-based neuroimaging

Eukaryotic cell lines

Policy information about cell lines

Cell line source(s)	All experiments were performed in a commercially available yeast YPH499 strain background (MATa ura3-52 lys2-801_amber ade2-101_ochre trp1-Δ63 his3-Δ200 leu2-Δ1). Selected mutants were generated and validated in this study.
Authentication	Auxotrophic strains were validated on minimial (Hartwell's complete) medium lacking the appropriate amino acids for plasmid selection with 2% glucose as carbon source. Cloned DNA was sequenced.
Mycoplasma contamination	N/A
Commonly misidentified lines (See ICLAC register)	N/A
