# Supplementary material for: Quantitative assessment of the determinant structural differences between redox-active and inactive glutaredoxins
Source: Nat Commun. 2020 Apr 7;11:1725. doi: 10.1038/s41467-020-15441-3 (PMC7138851; doi:10.1038/s41467-020-15441-3)
Supplement: Supplementary file 2 — Peer Review [file 41467_2020_15441_MOESM2_ESM.pdf]

Reviewers' comments:

Reviewer #1 (Remarks to the Author):

This manuscript describes a thorough, systematic analysis of the impact of specific residues/regions on the in vitro and in vivo catalytic activity of the class I Grxs. The authors previously demonstrated that yeast Grx7, their model class I Grx for this study, possesses two distinct glutathione (GSH) interaction sites that play specific roles in oxidation vs. reduction of substrates. Here they follow up that study using additional Grx7 mutants in order to better understand how specific regions affect the catalytic mechanism. Furthermore, they express Grx7 as a fusion with the GSH/GSSG redox sensor roGFP2 in order to assess its catalytic activity in disulfide reduction/oxidation of roGPF2 in live yeast cells, providing a physiological validation for their findings. In addition, they conclude that the extended loop preceding the active site in enzymatically inactive class II Grxs serves as an off switch for catalytic activity in the absence of Fe-S cluster binding. Overall the manuscript is clear, well-written, and meticulously detailed, providing novel insight into the structure/function relationships of specific residues in the Grx7 enzyme. However, a few points of clarification mentioned below will improve the manuscript.

1) Methods, p. 22, 1st line: This should be supplementary Table 10, not Table 7.
2) GSSCys vs. HEDS assay: It would be helpful to show the specific reaction steps catalyzed by Grx in these two assays (e.g., as shown in ref. 12).
3) P. 23, 3rd line from bottom: misspelled "homologous"
4) Fig.7a: For clarity, the $H_2O_2$ concentration corresponding to the symbol shapes in the graph should be stated in the figure caption. The same is true for Supp. Fig. 17a. The WT + DTT data should also be shown in Supp. Fig. 17a for the sake of consistency.
5) P. 11: Why are the WP and loop mutants of roGFP2-ScGrx7 found to be 80-100% oxidized at steady-state in yeast compared to 60% for WT roGFP2-ScGrx7? Do the authors have a hypothesis for this interesting observation? Why would these constructs be more oxidized compared to roGFP2 alone or with the inactive roGFP2-scGrx7(C108S) variant? The authors note that ScGrx7WP may be inactive with roGFP2 due the bulkiness of this substrate (p. 19), but this does not explain why roGFP2 is MORE oxidized with this variant compared to roGFP2 alone.

Reviewer limitations: I am not a computational biochemist so I cannot carefully evaluate the methods used to obtain the results shown in Figs. 8 and 9.

Reviewer #2 (Remarks to the Author):

Here, Liedgens and colleagues investigated the structural and molecular underpinnings of redox active and inactive glutaredoxins. The authors used ScGrx7 as a model protein and identified key residues from four distinct protein regions that are required for glutathione scaffolding. Additionally, the authors were able to induce modest redox activity in the inactive Grx when key portions of this protein were swapped with the same regional sites found in the redox active isoform. Overall, it is my opinion that this study provides important biochemical and structural insights into the glutaredoxin proteins and the proteomic differences between the enzymatically active and Fe-S cluster binding isoforms. This is a timely study given the renewed interest in understand the global effects of glutathionylation on cellular funcitons.
The only drawback in my opinion associated with this study is demonstrating that the authors did not provide any evidence that the site-directed mutants or that the inactive mutants that have been rendered active can induce global changes in the cellular redox glutathionylome. For instance, the authors could use their yeast line deficient in glutaredoxins to further investigate the role of the interconverted active glutaredoxin in reverse protein S-glutathionylation following exposure to extracellular stimuli (e.g. nutrient starvation, growth inducing factors) and then characterizing the

overall impact on the glutathionylome (e.g. immunoblot, mass spec, BioGEE pulldowns). From my perspective, such an experiment would provide value-added to what I believe to an important study and would really drive home the conclusions drawn by the authors.

Reviewer #3 (Remarks to the Author):

I have reviewed the manuscript titled “Quantitative assessment of the determinant structural differences between redox-active and inactive glutaredoxins”. The results and interpretation of the authors appears to be of high quality, and the conclusions are original and important in the understanding of glutaredoxin biochemistry, I recommend its publication with only minor revision.

There are nevertheless three methodological aspects that I’d like to point out and I’d like the authors to comment on.

1) HEDS Assay
Both GSSCys and HEDS assays use glutathione disulfide substrates, in the case of HEDS, the substrate is generated in situ by the reaction of HED with GSH during the incubation time. Surprisingly, the authors refer to the HEDS assay as if hydroxyl ethyl disulfide were the actual substrate, for instance in p. 6. “The hydroxyl group can affect the turnover of non-glutathione disulfide substrates”, when only GSSCys and HEDS assays were performed. Or in Fig. S6 where the x axes are in terms of [HEDS] instead of substrate. This is surprising as the authors have previously published authoritative articles on this assay.
There is an important quantitative limitation to the HEDS assay and is precisely the kinetics of formation of the substrate during the incubation time. The main problem is that the reaction between GSH and HED is very slow and not quantitative. So, not all HED is transformed into βME-SG in equilibrium, much less in the 2.5 minutes the authors use as incubation time. The kinetic study of this reaction and many others involving low molecular weight thiols reacting non enzymatically has been in the literature for the past 40 years, but researchers keep making the same mistake of equating HED with βME-SG. For the record, the rate constant of GSH with HED, according to (Szajewski, R. P., and Whitesides, G. M. (1980) J. Am. Chem. Soc. 102, 2011–2026) is 12 M-1 min-1 at pH = 8, meaning that the reaction would have a t½ ≈ 28 min with 2 mM GSH.
Using the reported kinetic parameters one can calculate the concentration of the substrate GSSEtOH at 2.5 min and see that the advance of reaction is evidently dependent on the initial concentrations. Also, when using different concentrations of GSH, the fraction of HED transformed into substrate is different so the hyperobolic fit to the Michaelis Menten equation would be differentially distorted under different conditions.

| [GSH]0 (mM) | [HED]0 (mM) | [GSSEtOH] @ 2.5 min | HED Transformed |
|---|---|---|---|
| 0.100 | 0.18 | 0.536 μM | 0.29 % |
| 0.100 | 0.74 | 2.18 μM | 0.29 % |
| 2.0 | 0.18 | 9.89 μM | 5.45 % |
| 2.0 | 0.74 | 40.3 μM | 5.39 % |

Finally, the numeric results obtained with the HEDS assay show KM values (for instance in Table S3) well beyond the highest concentration of HEDS used (0.74 mM).

2) Active site Cys pKa
This is a minor point. The results presented in Fig. 3 are remarkably compelling and show clearly a lack of effect of several mutations on the pKa of the active-site cysteine, the authors chose to fit their data to the Hill equation, which is a four parameter equation. Usually the sigmoidal plots of pKa determinations are fitted to the Henderson-Hasselbalch (a three parameter equation), and a Hill exponent ≠ 1 only makes sense if one suspects that more than one proton is exchanged in the acid-

base process.

3) $H_2O_2$ as oxidative insult
Again, in this section the results seem clear cut and solid and as such are unquestioned by this reviewer. The question is more about the choice of $H_2O_2$ as oxidant. The yeast used lack true glutathione peroxidases so the GSH and $H_2O_2$ are in separated kinetic compartments ($H_2O_2$ causes mainly the oxidation of the Trx systems). Then an interesting question would be what is the reaction sequence that produces GSSG or gluthionylated substrates. The authors should include gluthionylated substrates as potential oxidants that are detected by the Grx-roGFP2 probe, not only GSSG as stated. Understanding that sequence of reactions may shed light into potential artifacts causing positive or negative results when using different Grx.

**REVIEWER 1**

This manuscript describes a thorough, systematic analysis of the impact of specific residues/regions on the in vitro and in vivo catalytic activity of the class I Grxs. The authors previously demonstrated that yeast Grx7, their model class I Grx for this study, possesses two distinct glutathione (GSH) interaction sites that play specific roles in oxidation vs. reduction of substrates. Here they follow up that study using additional Grx7 mutants in order to better understand how specific regions affect the catalytic mechanism. Furthermore, they express Grx7 as a fusion with the GSH/GSSG redox sensor roGFP2 in order to assess its catalytic activity in disulfide reduction/oxidation of roGPF2 in live yeast cells, providing a physiological validation for their findings. In addition, they conclude that the extended loop preceding the active site in enzymatically inactive class II Grxs serves as an off switch for catalytic activity in the absence of Fe-S cluster binding. Overall the manuscript is clear, well-written, and meticulously detailed, providing novel insight into the structure/function relationships of specific residues in the Grx7 enzyme. However, a few points of clarification mentioned below will improve the manuscript.

1) Methods, p. 22, 1st line: This should be supplementary Table 10, not Table 7.

Thank you for noticing. We corrected the error.

2) GSSCys vs. HEDS assay: It would be helpful to show the specific reaction steps catalyzed by Grx in these two assays (e.g., as shown in ref. 12).

We added Fig. 1a, Fig. 2f and Supplementary Fig. 4f in order to improve the clarity regarding the specific reaction steps. The figure legends were modified accordingly, and the following sentences were added to the results section: "In this assay, ScGrx7 can directly react with HEDS, yielding 2-mercaptoethanol (2-ME) and a mixed disulfide between GSH and 2-mercaptoethanol (GSSEtOH). GSSEtOH has to change its orientation at the active site before it can be reduced by a second GSH molecule, yielding 2-ME and GSSG[12,25]."

3) P. 23, 3rd line from bottom: misspelled “homologous”

Thank you for noticing. We corrected the error.

4) Fig.7a: For clarity, the H2O2 concentration corresponding to the symbol shapes in the graph should be stated in the figure caption. The same is true for Supp. Fig. 17a. The WT + DTT data should also be shown in Supp. Fig. 17a for the sake of consistency.

We added the information to the figure legends of Fig. 7a and Supp. Fig. 17a ("The results for bolus treatments with 1 mM (circles), 0.2 mM (triangles) and 0.05 mM (squares) $H_2O_2$ are shown") as requested. Furthermore, we added the data for the controls +/- DTT to Supp. Fig. 17a.

5) P. 11: Why are the WP and loop mutants of roGFP2-ScGrx7 found to be 80-100% oxidized at steady-state in yeast compared to 60% for WT roGFP2-ScGrx7? Do the authors have a hypothesis for this interesting observation? Why would these constructs be more oxidized compared to roGFP2 alone or with the inactive roGFP2-scGrx7(C108S) variant? The authors note that ScGrx7WP may be inactive with roGFP2 due the bulkiness of this substrate (p. 19), but this does not explain why roGFP2 is MORE oxidized with this variant compared to roGFP2 alone.

This is indeed an interesting finding that we cannot currently fully explain but which will be the subject of a follow-up study. In particular, as part of an ongoing study, we are investigating the precise molecular mechanism of Grx-roGFP2 fusion constructs. One aspect of this study will focus on

the impact of Grx mutations on the ability of Grx to oxidize and reduce roGFP2 as a substrate. Our current model for the differential steady-state oxidation of the roGFP2 when fused to different Grx7 mutants, postulates that the kinetics of roGFP2 oxidation and reduction are differentially comprised. In other words, the ability of the Grx7 mutants to reduce roGFP2 is more severely comprised than the ability of these mutants to oxidize roGFP2. These differences might be coupled to conformational changes in protein areas iii and iv.

We modified the sentence on p. 19 accordingly: "Substrate- and conformation-dependent altered reduction and oxidation kinetics might explain why $ScGrx7^{WP}$ is active with GSSCys *in vitro* whereas bulky roGFP2 is predominantly oxidized in yeast."

*To further clarify: we are not suggesting that the Grx mutants can alter the thermodynamic equilibrium between 2GSH/GSSG and $roGFP2_{red}$/$roGFP2_{ox}$, clearly this is impossible. Instead, we speculate that other processes in the cell can lead to roGFP2 oxidation, albeit at very low rates. Known examples, would be interaction with thiol peroxidases. Ordinarily, these processes are considered kinetically insignificant in comparison to the Grx mediated oxidation and reduction of roGFP2. However, in a situation where the ability of Grx mutants to reduce roGFP2 is completely comprised we would expect roGFP2 to accumulate in an oxidized form. The assays developed in our study are particularly useful to monitor the ability of Grxs and mutants thereof to oxidize roGFP2. However, we do not yet have detailed information on the ability of these mutants to reduce roGFP2, which will likely require the development of appropriate in vitro assays.*

Reviewer limitations: I am not a computational biochemist so I cannot carefully evaluate the methods used to obtain the results shown in Figs. 8 and 9.

**REVIEWER 2**

Here, Liedgens and colleagues investigated the structural and molecular underpinnings of redox active and inactive glutaredoxins. The authors used ScGrx7 as a model protein and identified key residues from four distinct protein regions that are required for glutathione scaffolding. Additionally, the authors were able to induce modest redox activity in the inactive Grx when key portions of this protein were swapped with the same regional sites found in the redox active isoform. Overall, it is my opinion that this study provides important biochemical and structural insights into the glutaredoxin proteins and the proteomic differences between the enzymatically active and Fe-S cluster binding isoforms. This is a timely study given the renewed interest in understand the global effects of glutathionylation on cellular funcitons.

We thank the reviewer for his/her positive evaluation.

The only drawback in my opinion associated with this study is demonstrating that the authors did not provide any evidence that the site-directed mutants or that the inactive mutants that have been rendered active can induce global changes in the cellular redox glutathionylome. For instance, the authors could use their yeast line deficient in glutaredoxins to further investigate the role of the interconverted active glutaredoxin in reverse protein S-glutathionylation following exposure to extracellular stimuli (e.g. nutrient starvation, growth inducing factors) and then characterizing the overall impact on the glutathionylome (e.g. immunoblot, mass spec, BioGEE pulldowns). From my perspective, such an experiment would provide value-added to what I believe to an important study and would really drive home the conclusions drawn by the authors.

We thank the reviewer for his/her comments and suggestions. In our current study we have demonstrated that the impact of our various glutaredoxin mutations are consistent both *in vitro* and in intact living cells - with a completely different substrate, namely roGFP2. We therefore consider it very unlikely that any of our conclusions regarding (a) the relevance of protein areas (i) - (iv) for the interaction with the glutathione moieties of GSSR and GSH and (b) the role of the active site loop as a conformational OFF switch will be affected by further experiments.

The extension of our structure-function analyses to a differential analysis of redox proteomes/glutathionylomes is, in our opinion, beyond the scope of the current manuscript (i.e., the quantitative assessment of structural differences between active and inactive glutaredoxins). We fully agree that such experiments will be very interesting in the future to test, for example, the relevance of protein areas (i)- (iv) for *specific* Grx-protein interactions. However, this would surely represent a complete follow-up study in itself.

**REVIEWER 3**

I have reviewed the manuscript titled "Quantitative assessment of the determinant structural differences between redox-active and inactive glutaredoxins". The results and interpretation of the authors appears to be of high quality, and the conclusions are original and important in the understanding of glutaredoxin biochemistry, I recommend its publication with only minor revision.

We thank the reviewer for his/her thorough review and positive evaluation.

There are nevertheless three methodological aspects that I'd like to point out and I'd like the authors to comment on.

1) HEDS Assay
Both GSSCys and HEDS assays use glutathione disulfide substrates, in the case of HEDS, the substrate is generated in situ by the reaction of HED with GSH during the incubation time. Surprisingly, the authors refer to the HEDS assay as if hydroxyl ethyl disulfide were the actual substrate, for instance in p. 6. "The hydroxyl group can affect the turnover of non-glutathione disulfide substrates", when only GSSCys and HEDS assays were performed. Or in Fig. S6 where the x axes are in terms of [HEDS] instead of substrate. This is surprising as the authors have previously published authoritative articles on this assay.
There is an important quantitative limitation to the HEDS assay and is precisely the kinetics of formation of the substrate during the incubation time. The main problem is that the reaction between GSH and HED is very slow and not quantitative. So, not all HED is transformed into βME-SG in equilibrium, much less in the 2.5 minutes the authors use as incubation time. The kinetic study of this reaction and many others involving low molecular weight thiols reacting non enzymatically has been in the literature for the past 40 years, but researchers keep making the same mistake of equating HED with βME-SG. For the record, the rate constant of GSH with HED, according to (Szajewski, R. P., and Whitesides, G. M. (1980) J. Am. Chem. Soc. 102, 2011–2026) is 12 M-1 min-1 at pH = 8, meaning that the reaction would have a t½ ≈ 28 min with 2 mM GSH.
Using the reported kinetic parameters one can calculate the concentration of the substrate GSSEtOH at 2.5 min and see that the advance of reaction is evidently dependent on the initial concentrations. Also, when using different concentrations of GSH, the fraction of HED transformed into substrate is different so the hyperobolic fit to the Michaelis Menten equation would be differentially distorted under different conditions.
[GSH]0 (mM) [HED]0 (mM) [GSSEtOH] @ 2.5 min HED Transformed
0.100 0.18 0.536 µM 0.29 %
0.100 0.74 2.18 µM 0.29 %
2.0 0.18 9.89 µM 5.45 %
2.0 0.74 40.3 µM 5.39 %

We fully agree that the reaction between HEDS and GSH is very slow and that the interpretation of the kinetic data from the HEDS assay is complex (which is why we predominantly focused on the GSSCys assay in the current manuscript). Two major models have been proposed and applied during the past 40 years, one model emphasizing the nonenzymatic formation of GSSEtOH (2-ME-SG as pointed out by reviewer 3) and another model emphasizing a direct reaction between Grx and HEDS resulting in the enzyme-catalyzed formation of GSSEtOH. We thoroughly addressed and discussed both models in a previous study (Begas et al 2015 Chem Sci 6 3788-96). Figure S1 of this study shows calculations that are similar to the calculations by reviewer 3 in order to determine whether plotting initial GSH or HEDS concentrations or calculated GSH or HEDS concentrations results in distorted Michaelis-Menten graphs/fits. Best fits for the kinetic data were obtained assuming that GSH did *not* react with HEDS to a significant extent in accordance with a very slow nonenzymatic reaction:

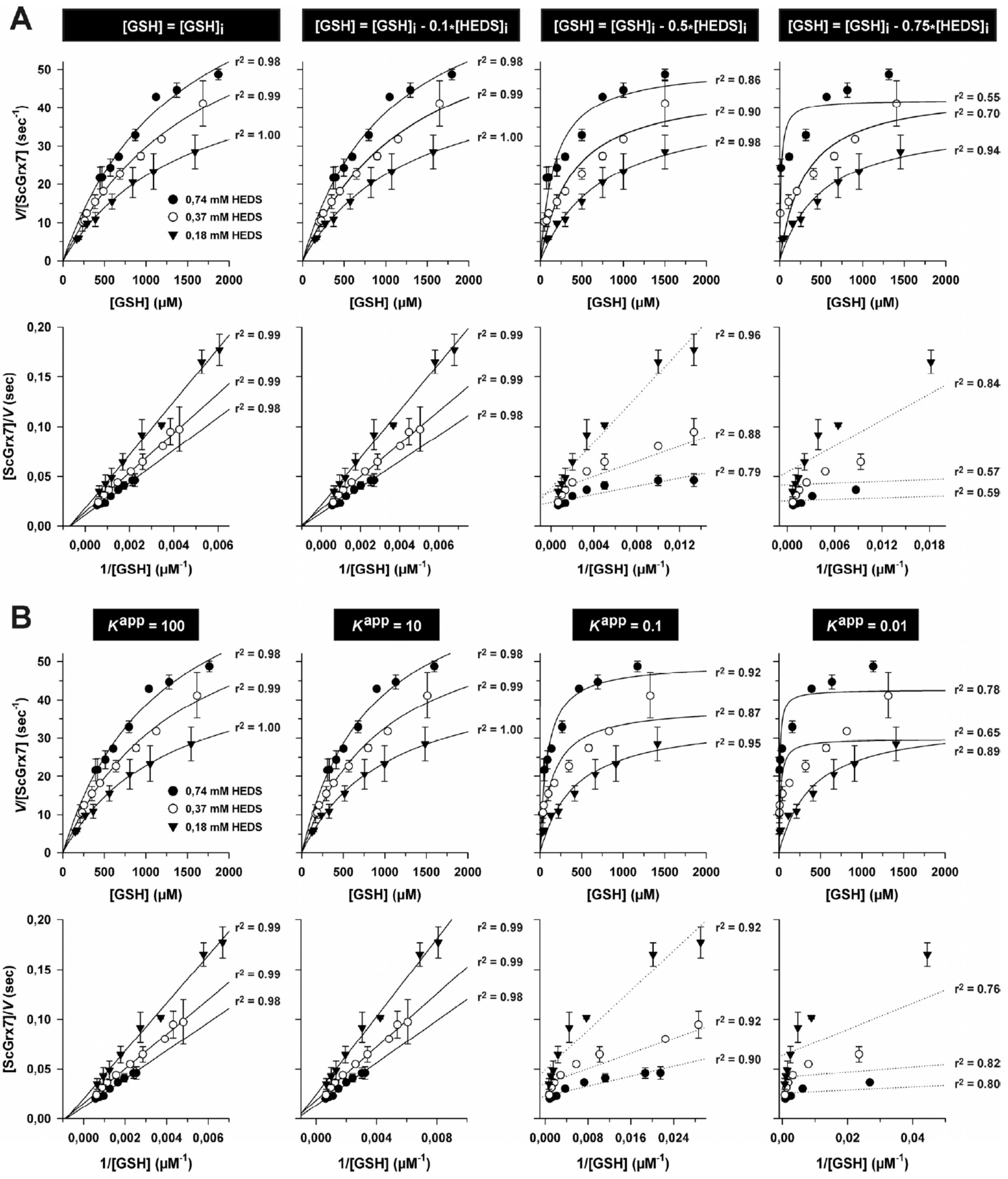


**Figure S1.** Alternative evaluation of the steady-state kinetics obtained for ScGrx7 in the HEDS assay at three different HEDS and variable GSH concentrations. In both panels, direct and Lineweaver-Burk plots are shown in the upper and lower row, respectively. (A) The concentration of GSH plotted on the x-axis was adjusted assuming a conversion of different amounts of HEDS in reaction 1 before the assay was started by the addition of ScGrx7. $[GSH]_i$ and $[HEDS]_i$ indicate the initial concentrations before pre-incubation. Representative plots for a putative conversion of 0, 10, 50 or 75% HEDS are shown from left to right. (B) The concentration of GSH was calculated using equation 1 based on hypothetical equilibrium constants. Representative plots for $K^{app}$ values of 100, 10, 0.1 or 0.01 are shown from left to right. Values for each data point in panels A and B were averaged from two independent experiments.

In the same study, we also estimated the apparent second order rate constant between HEDS and GSH in assay buffer (0.63 $M^{-1}s^{-1}$, which is even slower than the value in the JACS study from 1980) suggesting that almost no HEDS is nonenzymatically converted after 2.5 minutes pre-incubation. Furthermore, we determined in several studies the Grx activity in the HEDS assay after different pre-incubation periods. For example, see Fig. 5 in Begas et al 2015 Chem Sci 6 3788-96:

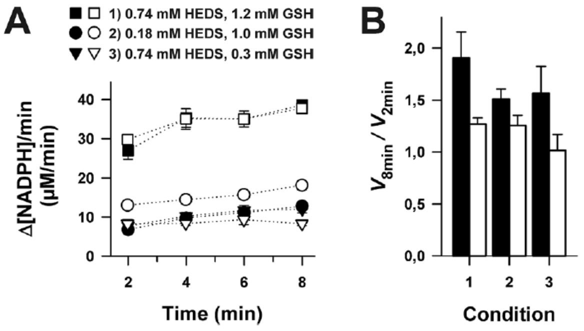


Fig. 5 Relevance of the pre-incubation period for the enzymatic activity in the HEDS assay. (A) Correlation between the reaction velocity and the length of the pre-incubation period in the absence of ScGrx7. All assays were performed with 12.5 nM ScGrx7 (closed symbols) or $PfGrx^{C32S/C88S}$ (open symbols) at the indicated conditions 1–3. (B) Ratio between the measured reaction velocities from panel A after 2 and 8 min pre-incubation. Values for each data point were averaged from three measurements.

All these data taken together, we came to the conclusion that the non-enzymatic reaction can indeed affect the detected reaction velocities after longer incubation periods but is overall much too slow to explain the high Grx activity and rapid turnover of HEDS (in particular, after short pre-incubation periods). Hence, our current interpretation of the kinetic data is (i) that HEDS is indeed a non-glutathione disulfide substrate and that (ii) we predominantly detect a direct reaction between Grx and HEDS after short pre-incubation periods. This interpretation is also supported by the sequential kinetic patterns of the HEDS assay if we consider the two different glutathione interaction sites (Begas et al 2017 Nat Commun 8 14835): GSSEtOH itself is turned over with ping-pong kinetic patterns (Fig. 2 in Begas et al 2015 Chem Sci 6 3788-96). However, direct reduction of HEDS by Grx and a subsequent reaction with GSH should result in enzymatically formed GSSEtOH that has the wrong orientation (with the glutathione moiety positioned at the activator site instead of the scaffold site). Thus, enzymatically formed GSSEtOH cannot be directly reduced by GSH and becomes an inhibitor. A successful reduction by GSH should only occur after re-orientation of GSSEtOH (with the glutathione moiety positioned at the scaffold site). The inhibitory effect of enzymatically formed GSSEtOH presumably causes the sequential kinetic patterns despite the double ping-pong mechanism.

We added Fig. 1a, Fig. 2f and Supplementary Fig. 4f in order to improve the clarity regarding the specific reaction steps for the GSSCys and HEDS assay. The figure legends were modified accordingly and the following sentences were added to the results section: "In this assay, ScGrx7 can directly react with HEDS, yielding 2-mercaptoethanol (2-ME) and a mixed disulfide between GSH and 2-mercaptoethanol (GSSEtOH). GSSEtOH has to change its orientation at the active site before it can be reduced by a second GSH molecule, yielding 2-ME and GSSG[12,25]."

Finally, the numeric results obtained with the HEDS assay show KM values (for instance in Table S3) well beyond the highest concentration of HEDS used (0.74 mM).

The referee is right that the apparent $K_m^{app}$ values for HEDS are above the used substrate concentrations (0.74 vs. 1-3 mM). It would be better to have at least five data points above and below the $K_m^{app}$ value. However, this is not a feasible option for the HEDS assay with ScGrx7 because of a decreased enzyme activity at higher HEDS concentrations (e.g., see Fig. 7A for 0.55 and 0.74 mM HEDS in Mesecke et al. 2008 Biochemistry 47 1452 - 1463):

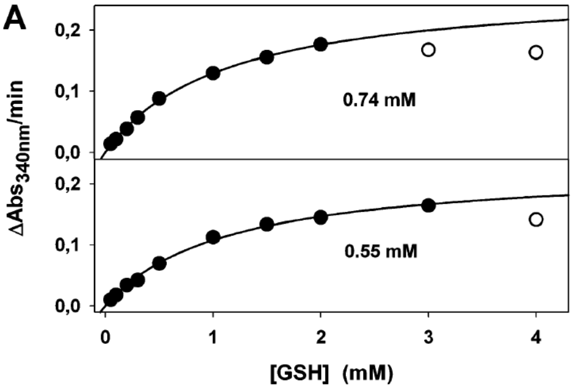


Due to this inherent limitation of the HEDS assay, we emphasized in the current and previous manuscripts that changes of the $K_m^{app}$ values for HEDS have to be interpreted with caution. For example, in Supplementary Figure 6 we stated: "Secondary plots of the $K_m^{app}$ values at different concentrations of HEDS (left panels) and GSH (right panels). Please note that the differences for $K_m^{app}$ values among independent measurements and mutants are mostly not statistically significant (see also Supplementary Table 3)."

2) Active site Cys pKa

This is a minor point. The results presented in Fig. 3 are remarkably compelling and show clearly a lack of effect of several mutations on the pKa of the active-site cysteine, the authors chose to fit their data to the Hill equation, which is a four parameter equation. Usually the sigmoidal plots of pKa determinations are fitted to the Henderson-Hasselbalch (a three parameter equation), and a Hill exponent ≠ 1 only makes sense if one suspects that more than one proton is exchanged in the acid-base process.

Data fits with three parameter equations yielded similar $pK_a$ values but deviated significantly from the data points at higher pH values. We therefore decided to empirically fit the data using a Hill equation. For example, shown on the right is the data for wild-type enzyme that was fitted in Sigmaplot according to (i) a Hill equation (black line) and (ii) the three parameter equation $y = y_0 - y_0 * (10^{pH-pKa}/(1+10^{pH-pKa}))$ from reference 22 (red line) yielding pKa values of 4.33 and 4.48, respectively. At the current stage, we cannot say whether the deviation at higher pH values is due to an additional protonation state of a proximal acid/base or whether another factor (e.g., a less reactive enzyme conformation) causes the residual activity around 10% above pH 6.5.

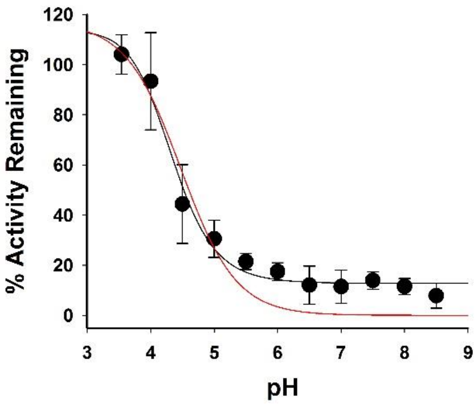

3) H2O2 as oxidative insult
Again, in this section the results seem clear cut and solid and as such are unquestioned by this reviewer. The question is more about the choice of H2O2 as oxidant. The yeast used lack true glutathione peroxidases so the GSH and H2O2 are in separated kinetic compartments (H2O2 causes mainly the oxidation of the Trx systems). Then an interesting question would be what is the reaction sequence that produces GSSG or gluthionylated substrates. The authors should include gluthionylated substrates as potential oxidants that are detected by the Grx-roGFP2 probe, not only GSSG as stated. Understanding that sequence of reactions may shed light into potential artifacts causing positive or negative results when using different Grx.

The referee correctly states that there are no *bona fide* glutathione peroxidases in yeast. Nonetheless, we have demonstrated in several recent studies that yeast readily produce GSSG upon $H_2O_2$ treatment, especially in yeast lacking glutathione reductase, as in this study. See for example the figure below from Morgan et al, Nat. Chem. Biol., 2013. $H_2O_2$ treatment, in a Δ*glr1* background, readily induces the formation of GSSG and thus an increase in the oxidation of the cytosolic glutathione redox potential reporter, Grx1-roGFP2 (left side) and in GSSG levels in a total cell lysate (right side):

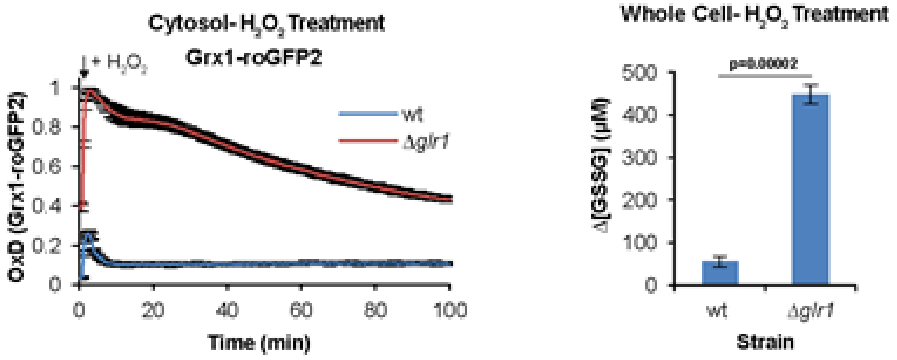


The most likely source of this GSSG are the peroxiredoxin proteins present in both the cytosol and mitochondrial matrix. For example, we recently demonstrated that the 1-Cys peroxiredoxin, Prx1, is the exclusive source of GSSG in the mitochondrial matrix following $H_2O_2$ treatment (see Calabrese et al, EMBO Journal, 2019). Figure 3B below is from this publication and shows the lack of response of a mitochondrial matrix-localized glutathione redox potential sensor (Su9-Grx1-roGFP2) in Δ*prx1* cells in comparison to wild-type cells:

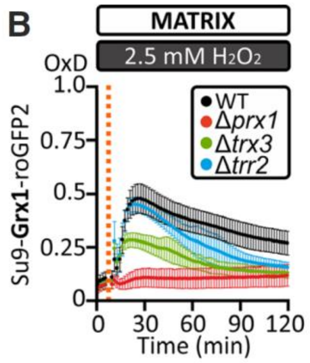

Glutaredoxins/GSH were also recently shown by the group of Christine Winterbourn to be able to reduce typical 2-Cys peroxiredoxins (Peskin et al, J. Biol. Chem., 2016). Tsa1 is the most abundant cytosolic typical 2-Cys peroxiredoxin in yeast. Our preliminary, unpublished data suggest that it is also an important source of GSSG in yeast cells. The response of a cytosolic glutathione redox potential sensor (Grx1-roGFP2), the steady state whole cell GSSG content, and the response of whole cell GSSG to $H_2O_2$ are all considerably decreased in a Δ*glr1*Δ*tsa1* strain compared to a Δ*glr1* strain:

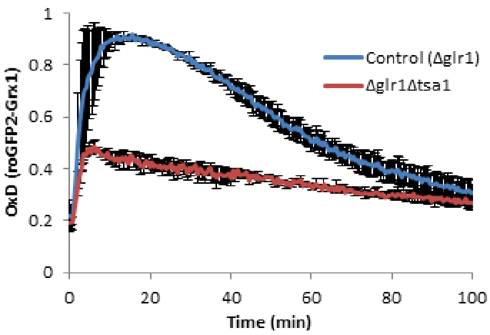


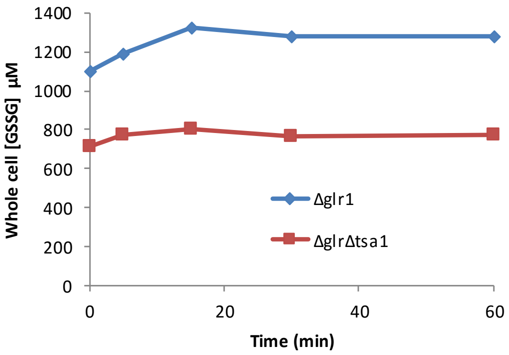


It is thus likely that peroxiredoxins are a (the) major source of GSSG in yeast cells upon $H_2O_2$ treatment. The glutaredoxins and mutants thereof (expressed as roGFP2 fusion constructs in our yeast cells) are therefore possibly also involved in GSSG production, via reduction of peroxiredoxins. These questions are also the subject of an ongoing study in our labs.

It is thus plausible that different Grx mutants influence the amount of GSSG that is produced in our assays in response to $H_2O_2$ treatment. However, given the very close correlation between our *in cellulo* and *in vitro* data we feel justified in our ultimate conclusion, namely, that roGFP2 fusions can be used to gain qualitative insights into Grx activity, mechanism and structure-function relationships. Whilst we could theoretically test alternative glutathione substrates, we do not feel that this would at all change our final conclusion and, importantly, we currently have no obvious way in which we could introduce such alternative substrates into our yeast cells in sufficient quantities.

To clarify these issues, we have added the following paragraph to the discussion:

'In our roGFP2 assays we choose to use $H_2O_2$ to initiate GSSG formation. Whilst yeast do not harbor any *bona fide* glutathione reductase, it was recently shown that GSH and/or glutaredoxins can reduce both 1-Cys and typical 2-Cys peroxiredoxins thereby leading to GSSG production[42,46,47,49,50]. Furthermore, GSSG was shown to readily accumulate in Δglr1 cells following H2O2 treatment[45]. We used Δ*glr1*Δ*grx1*Δ*grx2* cells for our assay, which lack any endogenous cytosolic glutaredoxin activity. Thus, the expressed roGFP2-Grx fusions will likely play a role in cytosolic GSSG production in our assays following $H_2O_2$ treatment. It is therefore possible that Grx mutants with impaired activity will affect GSSG production as well as roGFP2 oxidation. Consequently, with this assay, it is not possible to strictly separate the impact of Grx mutants on the capacity to reduce glutathionylated peroxiredoxins on the one hand and the ability to transfer this oxidation to roGFP2 on the other hand. Nonetheless, we consider that the consistent and robust correlation between our *in vitro* assays and *in cellulo* roGFP2 assays fully supports the conclusion that roGFP2-based assays allow rapid assessment of glutaredoxin activity, mechanism and structure-function relationships in living cells.'

REVIEWERS' COMMENTS:

Reviewer #1 (Remarks to the Author):

The authors have adequately addressed the reviewers'concerns.
